# Cohort‐Scale Spatial Autocorrelation for Tumor Prediction in Mid‐Infrared Pathology and Spatial Biomarker Discovery Using MALDI Imaging Lipidomics

**DOI:** 10.1002/advs.202516847

**Published:** 2026-02-09

**Authors:** Miriam F. Rittel, Nikolas Ebert, Denis Abu Sammour, Sebastian Graf, Björn C. Fröhlich, Emrullah Birgin, Shad A. Mohammed, Nuh N. Rahbari, Axel Wellmann, Oliver Wasenmüller, Cleo‐Aron Weis, Stefan Schmidt, Carsten Hopf

**Affiliations:** ^1^ CeMOS Research and Transfer Center Mass Spectrometry and Optical Spectroscopy Technische Hochschule Mannheim Mannheim Germany; ^2^ Medical Faculty Mannheim Heidelberg University Mannheim Germany; ^3^ CeMOS Research and Transfer Center Intelligent Systems Technische Hochschule Mannheim Mannheim Germany; ^4^ Institute of Pathology Heidelberg University Hospital Heidelberg Germany; ^5^ German Cancer Research Center (DKFZ) Heidelberg Germany; ^6^ Department of Surgery University Medical Centre Mannheim Mannheim Germany; ^7^ Institute of Pathology Celle Germany; ^8^ Interdisciplinary Center for Scientific Computing (IWR) Heidelberg University Heidelberg Germany; ^9^ Medical Faculty Heidelberg Heidelberg University Heidelberg Germany

**Keywords:** colorectal cancer liver metastasis, MALDI imaging, mass spectrometry imaging, mid‐infrared imaging, multimodal correlative imaging, spatial autocorrelation

## Abstract

Mid‐infrared (MIR) imaging is an emerging label‐free modality for classifying tissue types, including viable tumor in highly heterogeneous cancers, by assessing spatial differences in chemical composition. However, common data analysis neglects spatial vicinity and relies on time‐consuming pathological insight for hotspot prediction of viable tumor areas and computational tissue type annotation. Here, we present a method that uses spatial autocorrelation on MIR projection images computed from data of selected wavenumbers found by random forest ranking for computational tissue type annotation: Interdependent data processing enabled high accuracy annotations, whereas referencing of sequentially added new samples to a hyperspectral tissue database ensured computational efficiency and scalability for larger cohorts. Applied to clinical colorectal cancer liver metastasis samples, the method matched manual pathology assessment in a double‐blind study. As an option, MIR‐based hotspots can be correlated with mass spectrometry imaging. This multimodal approach identified sphingomyelin isoforms as lipidomic tumor marker candidates by imaging parallel reaction monitoring‐parallel accumulation serial fragmentation (iprm‐PASEF) directly on tissue. Taken together, spatial autocorrelation analysis on MIR imaging data could improve automated accurate annotation of tissue morphologies of heterogeneous cancer specimens and support the discovery of spatial cancer biomarkers.

## Graphical abstract

Computational annotation of various tissue types in heterogeneous samples such as colorectal cancer liver metastasis (CRLM) using spatial autocorrelation analysis on non‐destructive mid‐infrared (MIR) imaging data enabled correlative multimodal mass spectrometry imaging (MSI) for spatial investigation of lipid tumor marker candidates. The method can be implemented with minimal histopathology information, which renders it suitable for generation of computationally labeled tissue type datasets for sizeable clinical cohorts.

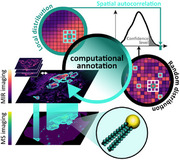

## 1 Introduction

Spatially resolved tissue analysis using omics technologies (“spatial omics”) have recently advanced the fundamental understanding of heterogeneous tissues such as many cancers at unprecedented speed [1]. Spatial omics can assess patterns of biomolecules such as lipids, peptides, mRNAs, small metabolites, N‐glycans and more at high spatial resolution and increasingly high speed. However, to utilize the spatial molecular information offered by these analysis techniques, molecular findings must be linked to biomedically relevant tissue morphologies, and this typically requires expert pathologists’ annotations. Although histopathological assessment is still the gold standard, accurate tissue annotation remains a manual and time‐consuming process. Therefore, it is often unsuitable for detailed pixel‐by‐pixel annotations of larger clinical cohorts. As a consequence, various attempts have been made to automate the complex assessment of many features such as cell size and shape, staining intensity, or position of nuclei [2]. However, due to the complexity of this assessment and shortage of trained pathologists and despite the advent of general‐purpose foundation models for computational pathology [3, 4, 5], the long‐term feasibility and utility of non‐molecular, entirely morphology‐based tissue annotation is presently unclear.

On the other hand, vibrational spectroscopy, including mid‐infrared (MIR), quantum cascade laser (QCL)‐based MIR, or Raman imaging, have emerged as powerful imaging modalities that record spatial overviews of biomolecular classes, e.g., nucleic acid‐rich, protein‐rich or lipid‐rich, that can be used for molecular pattern‐based tissue segmentation or co‐registration with histopathology [6, 7, 8, 9, 10, 11, 12]. Vibrational spectroscopy imaging technologies are non‐destructive and label‐free techniques that can complement classic histopathology for accurate tissue type segmentation and annotation based on spatial differences in chemical composition. In addition, they can be combined with downstream mass spectrometry imaging (MSI) for biomolecular identification [13, 14, 15, 16, 17, 18, 19, 20, 21].

Clustering algorithms are among the most commonly used data analysis tools for high dimensional MIR imaging data. However, they rely on a priori knowledge, such as the number of clusters or the assignment of clusters to a specific tissue type. These are often unavailable for heterogeneous clinical tissue samples [9, 15, 16, 17, 19, 20, 22, 23]. Therefore, clustering analyses require the definition of analysis parameters such as the number of clusters, and, for new samples, continuous assignment of tissue types to these determined clusters by expert pathologists. Machine learning (ML)‐based classification methods can operate more independently once trained, but require extensive ground truth training sets [24, 25]. Both methods commonly neglect the spatial context within a sample and work under the assumption that any region can be assigned to one of the tissue types provided during algorithm setup. An easy‐to‐use and easy‐to‐automate algorithm for assigning tissue types to segments, i.e., for defining ROIs, and for overcoming these limitations is spatial autocorrelation (SA) analysis. SA has already been implemented in other biomedical imaging fields [26], including MSI [27, 28], spatial transcriptomics [29], or histopathology [30]. However, SA has not been applied to mid‐infrared imaging data for computational tissue type annotation.

Over the past decades, spatial lipidomics has gained increasing biomedical interest, since various lipid classes have been linked to many essential biological processes as well as various diseases. Particularly in cancer, lipidomic alterations affect many basic biomedical functions including membrane structure, signaling, and bioenergetics [31, 32, 33]. Metabolic pathways involved in lipid dysregulation have been proposed as avenues for therapeutic intervention, diagnostic biomarker discovery and improved patient care in colorectal carcinoma (CRC) [27, 34, 35]. The multilevel heterogeneity In colorectal cancer liver metastasis (CRLM) in particular has not been investigated by spatial lipidomics, yet [36]. It has been noted that metabolite differences might be suitable predictors for postoperative disease recurrence in CRLM [36, 37]. Matrix‐assisted laser desorption/ ionization (MALDI) MSI has become a mainstay of spatial lipidomics [38, 39, 40, 41, 42]. Recently, laboratory workflows and dataset‐dependent acquisition strategies like spatial ion mobility‐scheduled exhaustive fragmentation (SIMSEF) and imaging parallel reaction monitoring—parallel accumulation serial fragmentation (iprm‐PASEF) have been presented that enable high‐performance liquid chromatography (HPLC)‐free, ion mobility spectrometry‐supported on‐tissue fragmentation analysis for high confidence lipid annotations directly on tissue [17, 43].

In this work, we developed a computational workflow for tissue type classification using MIR imaging data based on SA analysis of MIR projection images obtained by combination of multiple MIR images for distinct spectral features, i.e., informative wavenumbers, selected by permutation importance applied to a random forest classifier. This was followed by a specificity test. SA analysis was subsequently conducted to highlight regions associated with specific histological features. In variant 1 of this methodology, cohort‐wide processing of multiple samples in parallel further increased this workflow's annotation accuracy. To mitigate computational costs of cohort‐wide processing, an additional reference‐based SA analysis approach was implemented as variant 2. It allows fast analysis of cumulative samples against a previously calculated database, thus making it suitable for ongoing clinical studies and larger cohorts. Tissue type annotations were also validated in a double‐blind study against histopathological annotations. The SA analysis workflows on MIR imaging data can (but do not have to) also be combined with MSI for correlative MIR‐assisted MSI‐based candidate lipid biomarker discovery with iprm‐PASEF in clinical colorectal cancer liver metastasis (CRLM) [17].

## 2 Results and Discussion

### 2.1 Spatial Autocorrelation Analysis on MIR Imaging Data for Whole Section Computational Tissue Type Annotation in Cancer Specimens

In spatial multi‐omics approaches, labels specifying tissue types are required for complex and heterogeneous tissue specimen assessment. Ultimately, they are necessary for the identification of candidate spatial biomarkers, i.e., molecules that consistently co‐localize with a given tissue type of interest (TTOI) such as viable tumor (Figure S1). While such labels are typically provided via manual annotation by an expert pathologist, computational annotation based on MIR imaging in combination with clustering algorithms can be considered [22, 23]. However, the latter requires prior knowledge about the number of clusters, and the assignment of clustering results to TTOIs in heterogeneous cancer patient samples. While other methods such as machine learning approaches allow more independent annotation once trained, they are computationally costly, as they typically require high‐dimensional feature spaces, extensive training datasets, GPU‐accelerated computation, and long training times, as well as comprehensive ground truth training data for implementation. Both don't consider spatial context or the annotation of uncertainties regarding unknown or mixed tissue type regions. For a more detailed overview of these requirements including literature, see Table S1.

**FIGURE 1:**
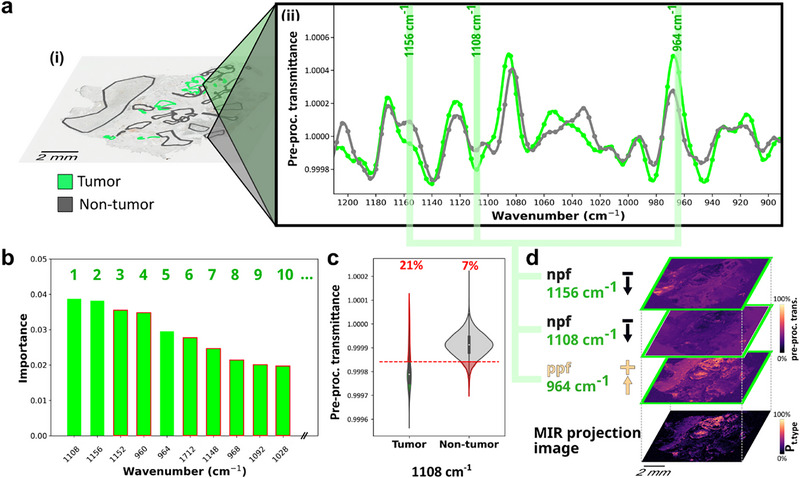
Random forest classifier‐based selection of wavenumber features to obtain tissue type specific single dimension image required for SA analysis. (a) (i) Tissue type annotations by a pathologist (on adjacent H&E‐stained section) were co‐registered, transferred, and used to assign (ii) MIR spectra (pre‐processed transmittance (normalized 2^nd^ derivative), cubic interpolation between data points of average spectrum (n = 500 random pixel subset per TTOI and non‐TTOI)) of corresponding tissue types of interest (TTOI). Discriminant feature analysis used a (b) random forest classifier to rank the importance of any feature to the classification of TTOIs. Subsequently, wavenumbers specific to any tissue type (exemplified here for tumor) were selected by (c) an error test with a cut‐off of less than 25% of pixels wrongly assigned for any tissue type (exemplified for tumor and non‐tumor false assignments (shaded red) for wavenumber feature 1108 cm^−1^). The remaining features passing the error test (red outline in (iii) failing error test) were (d) combined into a single‐plane projection image of the corresponding tissue type (P_t.type_) by pixel‐wise intensity value summation of positive predictive features (ppf, higher pre‐processed transmittance in tumorous regions) and subtraction of negative predictive features (npf, lower pre‐processed transmittance in tumorous regions) to create a 1D image for subsequent univariate SA analysis.

**FIGURE 2:**
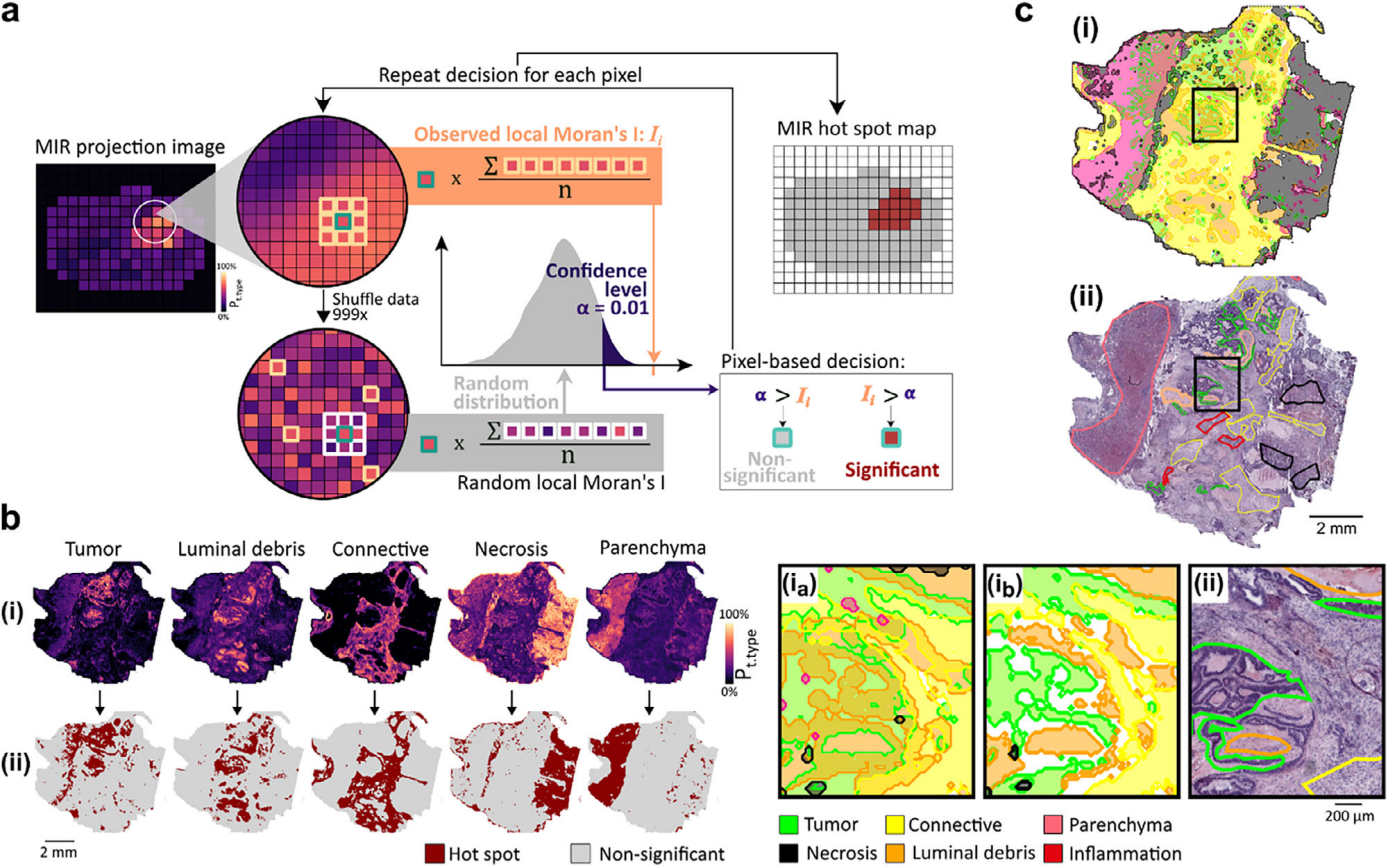
Spatial autocorrelation (SA) analysis on mid‐infrared (MIR) imaging data for computational annotation of tissue types in cancer specimens. (a) SA analysis of MIR imaging data: a subset of wavenumbers specific for a given tissue type of interest (TTOI) was selected from hyperspectral data (range 750 – 4000 cm^−1^) by discriminant analysis (random forest classifier and error test). MIR images of all wavenumbers from this subset were subsequently combined into a single‐plane projection image *P_t.type_* (Figure 1). To determine patterns corresponding to local SA of high intensity values, the observed local Moran's *I* at position *i* (*I_i_*) was compared to a random distribution and tested for significance at the corresponding pixel (confidence level α = 0.01, gray = non‐significant, red = significant). This process is exemplified for one pixel (green outline). The observed Moran's *I_i_* (orange) was calculated from the neighboring pixels (queen contiguity including eight neighboring pixels with orange outline), whereas the random local Moran's *I_i_* distribution (gray) were calculated from the shuffled neighboring pixels (white outline, shuffled 999‐times, green pixel retained). Repetition of this procedure for every pixel resulted in a MIR hotspot map of the entire section. (b) Masked projection images specific for TTOIs tumor, luminal debris (LD), connective tissue, necrosis, and parenchyma: discriminant feature‐based MIR projection images (i, top) and resulting MIR hotspot maps (ii, bottom). (c) Superimposed MIR hotspot maps color‐coded according to tissue type (i, top) in comparison to expert pathologist's annotation on H&E‐stained adjacent section (ii, bottom) with magnified example (rectangles). (i_a_) Multiple class assignments reflect the uncertainty at the boarders between tissue types, which were included in the comparison to pathological annotation and are a result of the analysis resolution using SA (i, 75 µm) as compared to microscopic resolution of histopathology (ii, 0.5 µm). (i_b_) Pixels which could not be specifically assigned to only one tissue type were omitted from later correlation with MSI to increase precision of the discriminant analysis. Inflammation was not computationally annotated due to the lack of specific wavenumber features.

To address these challenges, we developed a computational workflow for SA analysis on MIR imaging data as a tool for accurate, automated annotation of tissue types in cancer specimen. This workflow offers the annotation of tissue samples without any training that would be required for machine learning approaches. It is instead based on discriminant wavenumber features. We showcase in this work that these features can be selected either via literature research or be experimentally deduced from a single, annotated tissue section. They are robust enough to be transferred to the remaining samples of the study without the requirement for any further adjustment. This offers the possibility to generate a ground truth dataset, thus circumventing tedious and time‐consuming manual annotation, which could later be used for in‐depth molecular analysis and the implementation of more advanced computational methods such as deep learning approaches. The proposed workflow includes (i) automated wavenumber feature selection, (ii) the generation of a projection image from individual MIR spectral images to obtain a tissue type specific single dimension image (Figure 1), (iii) tissue type specific SA analysis, and (iv) addressing the limitation of assigning uncertainties of tissue type assignments to regions, i.e., pixels belonging to none of the proposed tissue types or to multiple ones (Figure 2a–c). This methodology is showcased for tumor tissue, and results obtained for other TTOIs present in the tissue samples are summarized in the supporting information (Figure S2–S7 and Table S2). To use different annotated tissue types as regions of interest (ROI) in spatial multi‐omics research, the procedure comprises additional steps including image co‐registration and transfer of whole‐tissue histological assignments to another imaging modality, here MALDI‐MSI.

Implementation of SA on MIR imaging data required the identification of spectral bands, i.e., wavenumber features associated with the TTOIs. For computational assessment of those features, tissue types were first annotated by an expert pathologist on a single H&E‐stained tissue section obtained from a CRLM patient. Subsequently, these annotations were co‐registered to the MIR imaged section to assign tissue type labels to the corresponding pre‐processed mean spectra (Figure 1a). Random forest (RF) algorithms have been used as tissue type classifiers in the MIR imaging field for years providing high robustness and excellent performance [44, 45]. Since RF, in contrast to more complex models, offers fast training, and resistance to overfitting, this type of algorithm is particularly suitable for identifying discriminative wavenumbers on a low number of training data sets [46]. Here, we employed a RF classifier to extract a small set of wavenumber features with the highest importance for the differentiation between the TTOI and all other tissue types (Figure 1b). The permutation importance applied to the RF classifier to rank features was repeated ten times, to account for sampling variance introduced via tissue type balancing (n = 500 pixels per tissue type based on the maximum availability of the least prevalent tissue type). The top10 ranked wavenumber features were further evaluated for their specificity in determining the number of pixels correctly assigned to the TTOI and any other tissue type by using a cut‐off (Figure 1c): Defined by the maximum acceptable error (set to 25%), wavenumber features passing this criterion and thus determined to be specific for a given TTOI. In most cases, only the top5 ranking features passed this specificity criterion. However, for some tissue types more features were sufficiently discriminating up to the top10 features (Table S2). Nevertheless, due to the drastic decrease in the number of relevant features in the second set of five, analyzing more features at an even lower rank was not reasonable. The wavenumber features discriminant for any specific tissue type were selected for projection into a single plane suitable for subsequent SA analysis (Figure 1d).

This procedure of wavenumber selection was repeated for all other TTOIs including luminal debris, necrosis, connective tissue, liver parenchyma, and inflammation (Figures S2 and S3 and Table S2). The process for computational wavenumber selection was conducted on a single patient sample that contained all TTOIs. Resulting wavenumber features were then utilized for all tissue specimens, i.e., samples from twelve patients diagnosed with CRLM. Pre‐processed transmittance images of the resulting discriminant spectral features were assessed visually for their spatial co‐localization with corresponding tissue types (Figure S4). An overview of the interconnection between data acquisition and analyses is provided as flow chart (Figure S5).

Additionally, these resulting wavenumber features were assigned to commonly described vibrational bands [47] (Figure S4). Here, wavenumber features specific to colorectal cancer cells (1156, 1108, 964 cm^−1^) could be allocated with the PO_2_^−^ (symmetric) vibrational band associated with nucleic acid‐rich tissues, as well as spectral regions associated with carbohydrates which might indicate increased metabolic activity of tumorous cells. Luminal debris‐associated wavenumbers (2872, 1172, 1168, 1132, 1128, 1040 cm^−1^) were found in close proximity within the same spectral range, which could hint at a functional connection of these two tissue types, since lumen are formed by the colorectal cells based on their inherent genetic programming. Wavenumbers specific to the surrounding connective tissue (1300, 1296, 1220, 1216, 1564, 1280, 1232, 1200 cm^−1^) were mostly present around the Amide III band and a close nucleic acid‐associated PO_2_^−^ (asymmetric) vibration, while liver parenchyma (1760, 1756, 1136, 1744, 1740 cm^−1^) showed strong differences to other tissue types in the lipid‐associated C‐O‐double bond (symmetric) vibration. Discriminant features of necrotic regions (1664, 1628, 1512, 1468, 1452, 1240, 1084, 1612, 1608, 1604, 1600, 1596, 1504, 1476 cm^−1^) were mostly allocated to the Amide I and Amide II vibrational bands associated with proteins. No wavenumber features were specific for inflamed regions. Especially an overlap with other nucleic‐rich tissue types such as tumor was also observed in another study [48]. However, since inflammation was not the main objective of this study, differentiation of this tissue type was not further pursued here. While these wavenumber features were determined independently in this study, several of them were described before to correlate with TTOIs investigated in this study of CRLM [23, 49, 50]. Specifically, wavenumbers associated with colorectal cancer cells (1156 cm^−1^), connective tissue including fibrotic, cirrhotic, and tumor stroma tissue (1280, 1234, 1202, 1216, 1220 cm^−1^), tumor‐associated necrosis often observed within the glands as luminal debris (1121, 1044 cm^−1^), as well as healthy liver parenchyma (1745 cm^−1^) have been described in the literature.

Whereas SA is most commonly implemented as univariate analysis, i.e., taking into account only one intensity value per pixel, projections of multiple features into a single plane can be used to increase the discriminant power beyond that of a single feature. This has been proposed for mass spectrometry imaging (MSI) applications without tissue‐specific differentiation of the feature's predictive direction (increased or decreased intensity values in the TTOI as compared to all other tissue types) [27, 51]. In analogy, we projected selective wavenumber features into a single plane (Figure 1d). To ensure that discriminant power between the TTOI and all other tissue types increased with each added feature, positive predictive features (ppf) with higher intensities in the TTOI were projected using summation, while negative predictive features (npf) with lower intensities in the TTOI vs. all other tissue types were projected using subtraction. This increased the discriminant power of the projection image beyond that of a single feature image (Figure S6).

The resulting MIR projection images were used for subsequent SA analysis of the clinical cohort of CRLM specimens (n = 12; Figure S7). SA was used to locate statistically significant hotspots of regions with local correlation of high‐intensity values. To this end, local Moran's I using a randomization (shuffling) method was used to estimate the random distribution determining the significant regions (Figure 2a). Significance of the observed spatial relationship between a single pixel and its eight neighboring pixels (queen contiguity) was evaluated against a given confidence level α of 0.01. The process was conducted for all TTOIs to obtain whole‐section MIR tissue type annotations (Figure 2b). Overlay of all individual MIR hotspot maps specific to the corresponding tissue types resulted in the automatic annotation of the entire section (Figure 2c). Cross‐validation against the expert pathology annotation indicated consistent results. While precise manual annotation is often a compromise between time, area annotated, and precision of the annotation, SA based on automatically selected specific wavenumber features can be automated and performed on entire sections.

Clustering algorithms that have been applied in MIR imaging for computational tissue annotation require ground truth knowledge for the assignment of TTOIs to the delineated ROIs and for selection of the most suitable number of clusters used by the algorithm. Moreover, most commonly used clustering methods assign each pixel to the nearest cluster and cannot recognize if pixels cannot be assigned to any of the provided tissue types. Furthermore, in most commonly used clustering algorithms each pixel is only assigned to a single tissue type, thus neglecting possible mixed composition. In contrast, SA mitigates these uncertainties by enabling the assignment of multiple tissue types to a single pixel or withholding assignment altogether when pixels do not correspond to significant hotspots (Figure 2c,i_a_). Awareness of these uncertainties allows users to select only specific pixels and to exclude unknown or mixed regions from subsequent analysis (Figure 2c,i_b_), thus potentially increasing precision. Although SA demonstrated accurate tissue type annotation based on MIR data, it remained susceptible to false positives in single homogeneous samples, thus necessitating the implementation of interdependent processing approaches.

### 2.2 Cohort‐Wide SA Analysis of Multiple Colorectal Cancer Liver Metastases (CRLM) Samples Improves Annotation Accuracy Compared to Individual SA Analysis

While our results in the previous section indicated that SA analysis on MIR imaging data was suitable for computational, i.e., automated, annotation of different tissue types present in CRLM samples, susceptibility to false annotation in rather homogeneous samples was also noted. To mitigate this effect, interdependent processing of multiple samples in correlation to each other was evaluated. SA analysis identifies any pattern of relative increased intensity as compared to a randomized intensity distribution of the same sample. For this reason, fairly homogenous samples that are, for instance, often observed in small sections like biopsies or tissue micro array (TMA) cores, can be prone to false annotations. Therefore, a cohort‐wide processing procedure for SA was developed and implemented that considers all samples of a cohort simultaneously during SA analysis.

**FIGURE 3:**
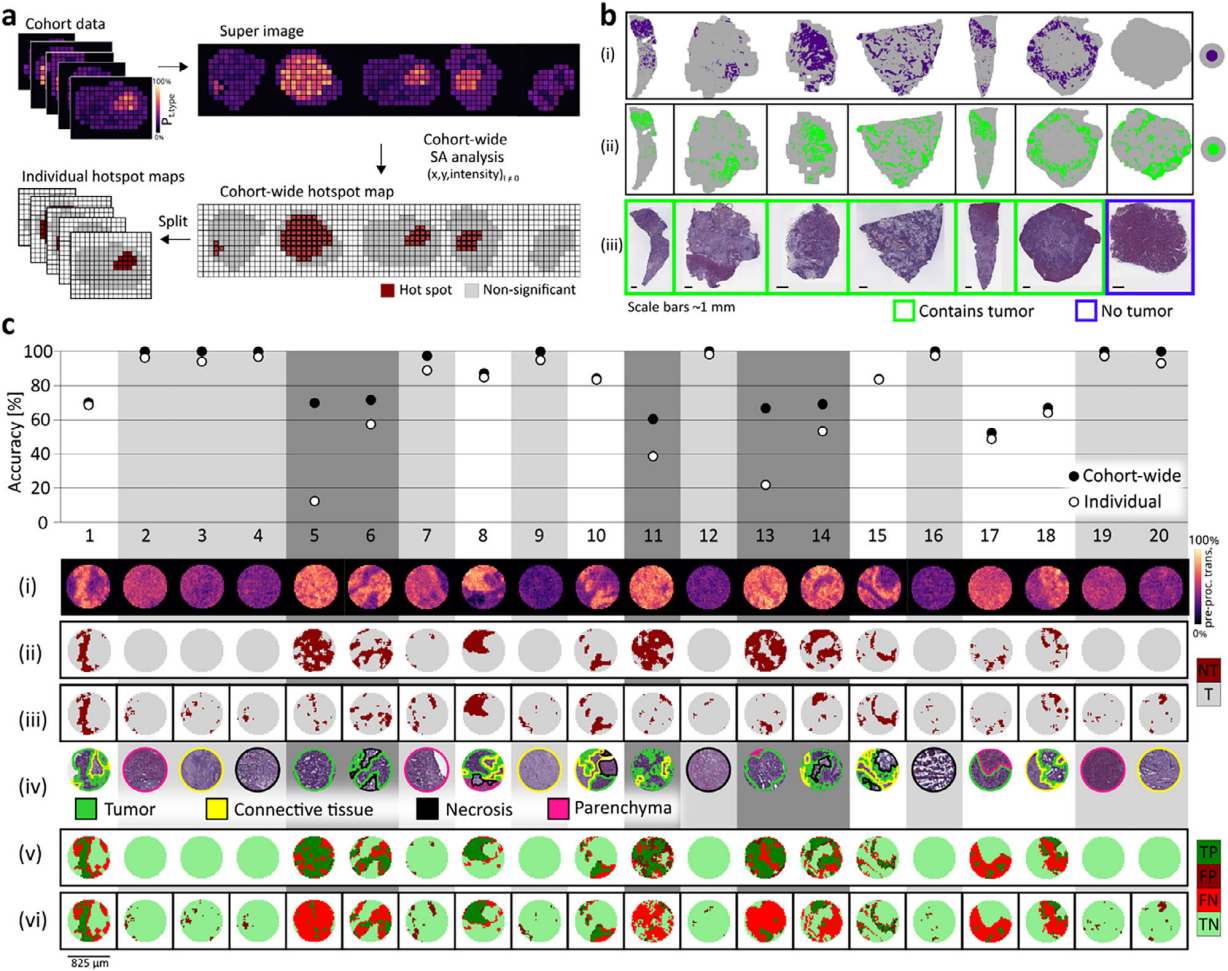
Cohort‐wide SA analysis enhances accuracy of tissue type annotations. (a) Schematic overview: All individual tissue samples from a patient cohort were combined into a super image via zero‐padding to achieve the same size for each image necessary for side‐by‐side stitching. This super image was then subjected to SA where all pixels from each tissue except the one currently under investigation were included in the shuffling process that generates the random distribution used for significance testing. This resulted in a single MIR hotspot map of the entire cohort, which was then divided into individual samples. (b) Cohort‐wide SA enabled correct tissue type assignment even for homogenous samples: Seven colorectal cancer liver metastasis (CRLM) tissue sections (six cancer specimen, green outline; one non‐tumor liver parenchyma specimen, blue outline). Cohort‐wide processing of SA (i) but no single sample SA processing (ii) correctly assigned no “tumor” regions to the homogenous, non‐tumor liver parenchyma. H&E‐staining reference (iii). Calculations were performed on projection images of normalized 2^nd^ derivative of transmittance at 964 cm^−1^, 1156, 1108 cm^−1^. (c) Twenty tissue micro arrays cores were digitally punched from MIR images of resected CRLM tissues using a common biopsy needle diameter (G18, scale bar 825 µm). Tumor‐specific pre‐processed transmittance at 964 cm^−1^ indicated tumor‐containing areas (i). MIR hotspot maps were calculated by cohort‐wide SA (ii) or individual SA (iii) for each core and compared to pathological annotations (iv). Accuracies for cohort‐wide SA (v) and individually processed SA (vi) (plotted on top for each core). true positive (TP, dark green), false positive (FP, dark red), false negative (FN, light red), true negative (TN, light green).

To this end, MIR projection images of individual samples of a cohort were combined into a “super image” so that a hotspot map was calculated for the entire cohort as a batch and thus in relation of each other (Figure 3a). While off‐tissue pixels were omitted from the analysis, every on‐tissue pixel was submitted to SA analysis and included in the randomization process to estimate the super image's random distribution. As a result, all samples of the cohort were used simultaneously when any single pixel in accordance with its neighborhood was tested for significance. As a key advantage of cohort‐wide processing, homogenous samples were analyzed in the context of other samples instead of being analyzed within their own narrow intensity distribution. This renders the analysis more robust and avoids the assignment of false positives and false negatives in homogenous samples. While cohort‐wide analysis of multiple samples has not been used for SA analysis on MIR data, it is known for clustering approaches [22, 49] and is regularly applied in advanced ML algorithms [14, 24, 49]. However, much more prior knowledge is needed for their implementation, as extensive ground truth training data is necessary for ML approaches, and the correct number of classes and the assignment of TTOIs to any class needs to be determined for clustering algorithms (Figure S8).

To test cohort‐wide processing of SA, two scenarios were investigated. First, analysis was performed on the entire section of resected CRLM tissue samples, which are commonly heterogeneous (Figure 3b). Tumor specimen heterogeneity implies that each sample likely contains not only tumorous but also non‐tumorous tissue types. In this scenario, benefits of cohort‐wide processing of SA were most pronounced for control samples like tumor‐surrounding liver parenchyma. Here, when SA was calculated individually for such a negative control sample more than 20% of the section were falsely annotated as tumor. In contrast, following cohort‐wide SA calculation, the entire sample was correctly recognized as being entirely non‐tumorous (Figure 3b). For the second testing scenario, biopsy cores the size of a standard biopsy needle were digitally punched from spectral images of resected CRLM tumor tissues, for which pathological annotations were available (Figure 3c). Due to the small sample size, homogenous composition of samples is more likely to appear even for inherently heterogeneous CRLM. The individual SA analysis of each core was compared to the cohort‐wide processed SA of all cores combined. This comparison revealed that especially in samples where >50% of the total area was tumorous, the extent of tumor was vastly underestimated and false negatively annotated as being Non‐tumor (Figure 3c). Also, in cores that only contained non‐tumorous tissue types, more false positive assignments were observed when SA was calculated individually instead of via cohort‐wide processing. Since SA analysis identifies significant co‐localization of high values (hotspots) within a given dataset, calculations performed on single, unbalanced cores (majority containing tumor or non‐tumor pixels) leads to the annotation of the most significant subregions within that individual core resulting in false positive or false negative annotations.

In contrast, when performing SA analysis on all cores in cohort‐wide processing, the average distribution of intensity values of all samples places such extremes into context. In this study, cohort‐wide processing of SA improved the accuracy of tissue type annotations. Especially, for homogenous samples or small samples where a homogenous composition is more likely, cohort‐wide processing can substantially impact the quality of the results. Furthermore, even datasets recorded at vastly different time points, i.e., 2.5 years apart, could be calculated via the cohort‐wide approach without any significant decrease in annotation accuracy (Figure S9). This robustness was observed despite variance caused by batch‐effects, different sectioning planes, environmental as well as operational changes were not only expected, which could clearly be observed in pre‐processed raw data between the two cohort datasets (Figure S10). However, this emphasizes the strength of the proposed MIR imaging workflow using multiple discriminant wavenumbers fused to a single projection image, and the analysis via spatial autocorrelation (SA). This suggested that analysis of individual samples could be performed even years after the original cohort was measured and still result in high accuracy annotations when calculated via a cohort‐wide SA approach.

### 2.3 Reference‐Based SA Analysis for Fast Annotation of Single New Tissue Specimens

Despite the apparent benefits of cohort‐wide processing over computation of individual samples, SA analysis remains a memory‐ and time‐extensive analysis procedure. For example, cohort‐wide SA analysis of 23 tissue samples required about 45 min computation time on a high‐performance computing system, while a normal desktop computer could not compute more than 11 of the samples due to limited computing power, thus limiting cohort‐wide processing to smaller cohorts of resected tissues (Figure S11).

**FIGURE 4:**
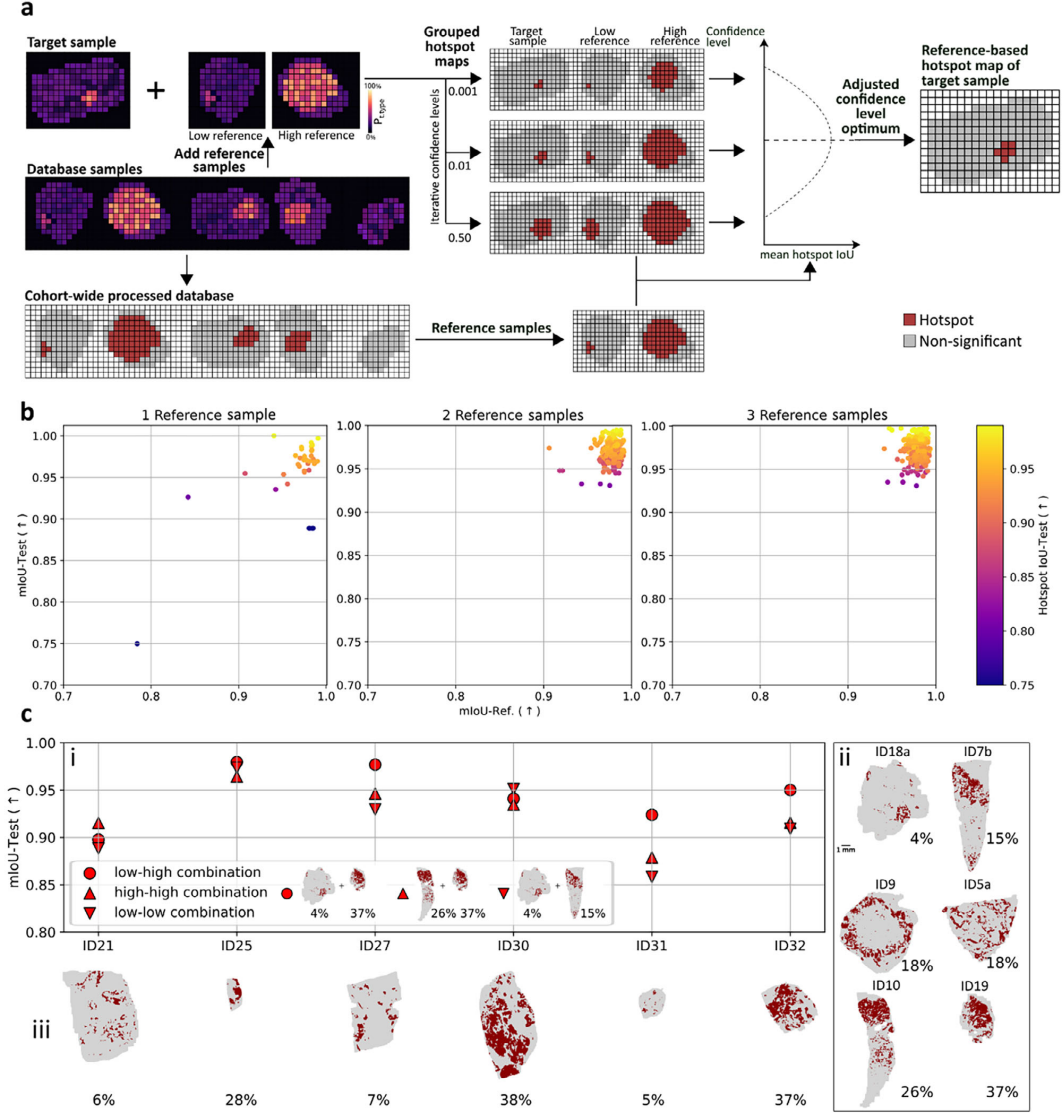
Reference‐based SA for fast annotation of cumulatively added specimen. (a) Schematic overview of reference‐based SA: A new sample is combined with two reference samples taken from a previously computed database, i.e., a SA cohort‐wide processed initial sample cohort (for example, from a clinical study). SA is calculated for this group of three. Subsequently, for 1000 different confidence levels (0.0005–0.5000; steps of 0.0005) hotspot maps of the two reference samples are compared to their original composition in the database to determine the confidence level providing the best fit, using mean intersection over union (mIoU) of hotspots of both reference samples. This level is utilized to automatically adjust the hotspot map of the single newly added sample and thereby reference it against the original database. (b) Two was determined to be the optimal number of reference samples. Note that performance increased from 1 to 2 reference samples, i.e., values accumulated toward the upper right, but no further with a third reference sample. x‐axis: fit of the reference samples; y‐axis: fit of hotspot the single newly added test sample both considering the hotspot as well as the non‐significant area; color code: fit of the hotspot of the newly added test sample; procedure compared test samples between cohort‐wide processed SA as reference model and reference‐based processed SA excluding the test sample from the database. (c) (i) Selecting the two tissues with the lowest and highest (circle; low‐high combination) hotspot‐to‐total tissue area ratio typically led to higher performance than other choices of reference samples such as the two lowest (downward triangle; low‐low) or two highest (upward triangle; high‐high) hotspot‐to‐total tissue area references. (ii) Six samples were used as database, which was calculated using the cohort‐wide SA approach. (iii) Six different samples were individually added, combined with two reference samples from the database (representing the according hotspot‐to‐total tissue area ratio combination, high‐low, low‐low, high‐high) and underwent reference‐based SA analysis. Percentages are hotspot‐to‐total tissue area.

For this reason, a second computational approach, henceforth called reference‐based SA analysis, was developed and implemented as a potentially more suitable option for incrementally added samples in long‐term clinical studies. In this method, single new samples are referenced against the original cohort of *n* samples by using only a subset *m* of the cohort, instead of recalculating SA for the entire expanded *n+1* cohort using cohort‐wide processing (Figure 4a). To do so, reference samples *m* were chosen from the original cohort and combined with the (1) single new sample into a set of three samples that were then cohort‐wide processed m+1. To reference the result $\hat{R}$ to the results of *R* representing the entire original sample cohort *n*, the confidence level *α* was iteratively adjusted in a way that the reference samples fit their original hotspot map composition as calculated in the database. The optimal adjusted confidence level α*, at which the areas of the hotspots of the two reference samples match those in the original database was employed to define the hotspot map of the single newly added sample. The similarity between the database and the references is calculated using the Intersection over Union (IoU), which was maximized. By means of this adjustment with an optimized confidence level, the added sample is indirectly referenced against the entire original cohort (Figure 4a).

To determine the optimal number of reference samples, six out of twelve CRLM samples were used as a reference database, and each of the remaining six samples used as individually added samples in the reference‐based approach. As a model for performance evaluation, the six samples of the reference database plus the single newly added sample were computed via cohort‐wide processing (Figure S12). This was repeated for each of the six samples used as “single newly added samples”. Permutations using one, two, or three samples of the reference database were used to calculate the newly added sample via reference‐based SA. While there was a significant performance increase from one to two samples used as a reference, adding a third reference sample did not enhance performance, but increased analysis time (Figure 4b). Therefore, henceforth two samples were used as reference for this methodology.

Next, definition of which two samples from the database would perform the best as references was required. It was noticed in the previous permutations that using two reference samples with the lowest and highest ratio of hotspot‐to‐total tissue area may consistently provide good results (low‐high‐combination). To test this hypothesis, controls with the two lowest (low‐low) or to highest (high‐high) ratio of hotspot‐to‐total tissue area were picked from the reference database. It was observed that the low‐high references consistently resulted in high performance, whereas the low‐low and high‐high references led to highly variable performance (Figure 4c). Performance was evaluated via (i) the fit between results of the reference‐based SA approach and the reference model (database plus newly added sample calculated via cohort‐wide SA), for the (iii) six single newly added samples as compared to (ii) the database. To determine whether the confidence level adjustment could be estimated via a simpler correlation, the relation between the new, adjusted confidence level in the reference‐based approach and the hotspot‐to‐total tissue area was investigated using two reference samples (low‐high‐combination) for the six test samples. These results showed that hotspot calculation not only depend on the size of the hotspot, but also the shape and intensity value distribution (Figure S13). This renders the iterative process of confidence level adjustment important for the reference‐based SA calculation. Reference‐based SA was also tested for homogenous control samples (Figure S14). Here, the same high performance as for cohort‐wide SA was observed, whereas individual processing of SA falsely assigned tumorous regions to negative control sample that only contained liver parenchyma excised from the proximity of the CRLM nodule.

In summary, the implementation of the reference‐based approach enabled the SA analysis of larger cohorts on a normal desktop computer reaching comparable performance quality as cohort‐wide processing. Also, individual samples incrementally added to the cohort throughout the period of a study could be calculated in about 6 min, in contrast to cohort‐wide processing, which becomes increasingly time‐consuming as the cohort grows. SA depends on local intensity patterns and their intensity differences, but these spatial patterns inherently vary between tissue compositions (percent of cross‐section belonging to the tissue type of interest), which cannot be equalized by intensity normalization alone. Selecting one reference sample with a low hotspot‐to‐area ratio and one with a high ratio provides an approximation of the cohort's distributional range, which lets the reference‐based approach reproduce cohort‐wide results without recalculating SA on the full dataset. Examination of the underlying processes suggested that higher annotation accuracy using SA analysis could only be achieved via interdependent processing, either by increasing the sample number and thus compositional variance (cohort‐wide SA approach) or by adjusting the confidence level in relation to previously calculated reference samples (reference‐based SA approach), respectively (Figure S15).

### 2.4 Double‐Blind Validation of Automated MIR Imaging‐Based Tissue Type Annotations Versus Manual Histopathological Annotations

**FIGURE 5:**
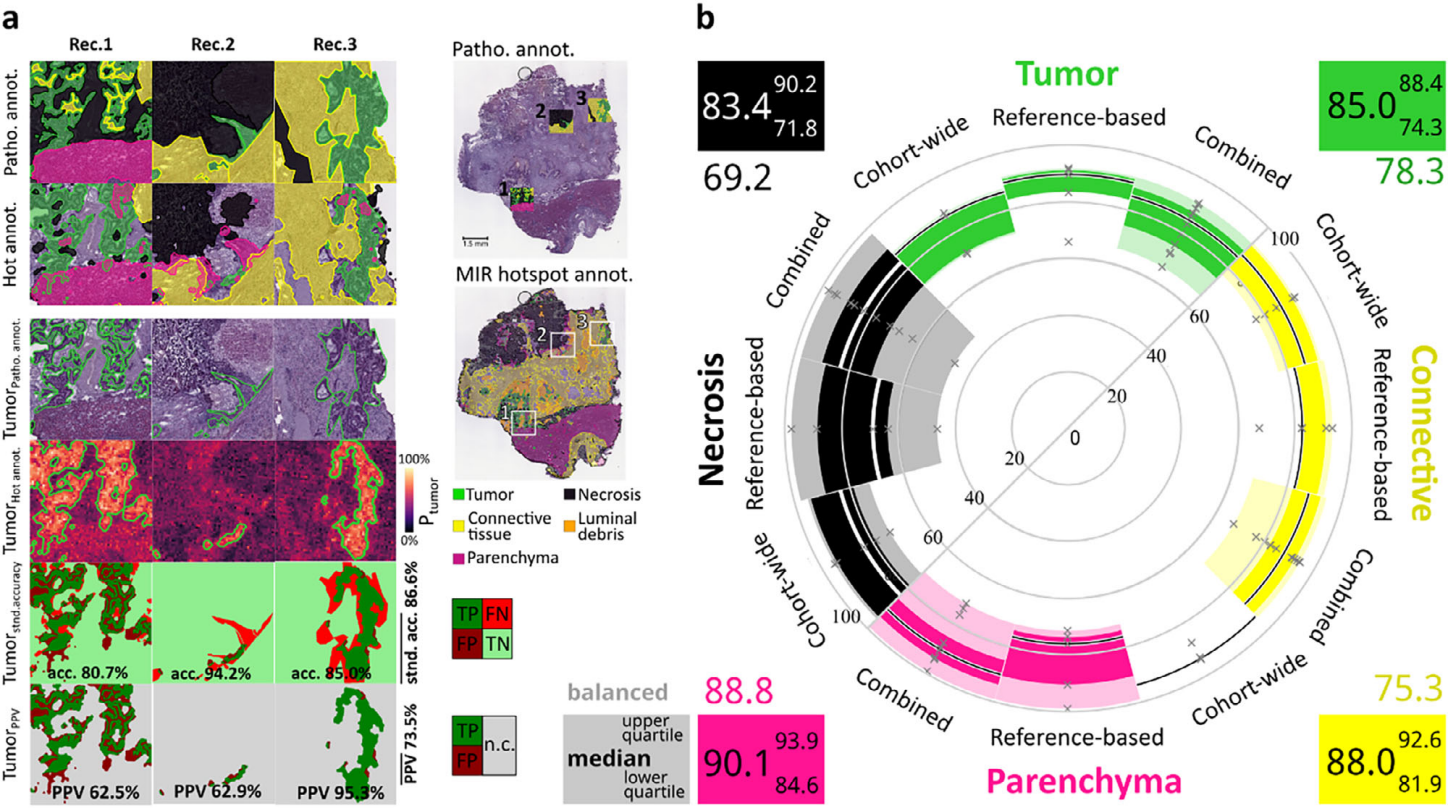
Double‐blind validation of MIR imaging‐based tissue type annotations vs. histopathological annotations. (a) For quality assessment, three rectangles (Rec.) per n = 12 patient samples (specified in Table S4) were annotated by a pathologist and compared to tissue type annotations based on MIR imaging and SA‐based hotspot analysis performed either by cohort‐wide processing (6 samples) or reference‐based processing (6 samples, double‐blind evaluation). Example shown for patient ID18a, (true positive (TP, dark green), false positive (FP, dark red), false negative (FN, light red), true negative (TN, light green), not considered (n.c., gray), positive predictive value (PPV), standard accuracy (stnd. acc.). (b)  Polar box‐plots for accuracies of MIR imaging‐based tissue type annotations. Each tissue type was analyzed separately, and mean standard accuracies were obtained from the three rectangles per patient and plotted for both groups (gray x), cohort‐wide processed (6 samples) and reference‐based processed (6 samples), as well as both groups combined regardless of the processing method (12 samples). Interquartile range (IQR) surrounding the median accuracy (full hue‐colored shapes with thick line), and the largest value within the 1.5xIQR (transparent shapes) are provided to judge the variance of annotations for individual samples. Median standard accuracies were above 80% for all tissue types. Numbers in the corners are colored by tissue type, median standard accuracy (large number) and lower/upper quartile (lower/upper small number); balanced accuracy values to judge the overall performance of SA as a classifier equalizing tissue type prevalence were calculated from all annotations of all specimen (colored numbers in corners). All accuracy values provided in percent.

To investigate the accuracy of computational tissue type annotations based on cohort‐wide or reference‐based SA analysis performed on MIR imaging data, these were compared to ground truth pathological annotations performed on adjacent, H&E‐stained tissue sections. Here, the twelve resected tissue samples of the CRLM cohort were split into two datasets of six samples each, one for SA analysis with cohort‐wide processing, the other one for reference‐based SA processing. Both sample sets were compared against precise pathological annotations on three rectangles per patient sample that contained all tissue types present in that particular sample (Figure 5a and Figures S16–S27). Annotation of the second set of six samples was performed in a double‐blind fashion.

Accuracy was chosen as an evaluation metric, accounting for the correct assignment of both the TTOI and non‐TTOI regions. Each tissue type was evaluated independently, since annotation of pixels is dependent on the choice of TTOIs constituting multiple tissue type assignments per pixel. Due to the natural co‐localization of tissue types, rectangles did not necessarily contain a representative percentage of the tissue type prevalence as observed in the entire section. Standard accuracy, which measures how many pixels are correctly assigned out of all pixels neglecting class imbalance, allows judgment even if the TTOI is not contained at all in the sample, and is less subjective to sampling effects (Figure S28). Therefore, it was selected for detailed analysis of individual samples to determine deviations between pathology‐ and MIR imaging‐based annotation. Balanced accuracy (*BA = (sensitivity+specificity)/2*) was used to judge the overall classifier performance of all samples combined, considering overall different prevalence of classes e.g., tissue types. Here, correct assignment of TTOI and non‐TTOI (sensitivity and specificity) are balanced, thus equalizing potentially differing occurrences of both. Additionally, the precision (positive predictive value) of the tumor annotations was determined for every sample individually. The standard accuracy was investigated for both SA methods separately (cohort‐wide and reference‐based, six samples each) as well as for all twelve samples combined regardless of the computation procedure (Figure 5b). Information on the samples (like disease stage, gender, and age of the CRLM patients) used for the evaluation of the SA method is provided in Table S4.

Overall, the accuracy of computational annotations did not significantly vary between cohort‐wide processing and reference‐based processing. Consequently, performing the analysis of the first set of six samples without a double‐blinded approach apparently had no influence on the results. Therefore, the double‐blind analysis of the second sample set suggested that SA could be performed independently from a pathological ground truth, once wavenumbers specific to the TTOIs are selected. Observed variances were likely a result of the small sample numbers and variations in tissue type composition. Results of individual sample analysis of both SA methods combined showed a median standard accuracy well above 80% for all tissue types. For instance, median accuracy for “tumor” was 85.0% with lower and upper quartiles of 74.3% and 88.4%, respectively. Here, outliers were investigated for the cause of deviations between automated MIR‐based vs. manual histopathological annotation techniques. These could be attributed to the limited spatial resolution of the MIR imaging measurement (25 µm, or, accounting for neighborhood incorporation in SA, 75 µm analysis resolution) (Figure S29). While other studies reported performance values between 90 – 100% (78.8% necrosis) they were conducted using more complex machine learning approaches on higher resolution MIR imaging data (6.25 µm/2.7 µm, 1.1 µm), which was shown in this study to have an influence on the performance result. In addition, in some cases chemical compositions of different cell types may not be sufficiently different in MIR‐based annotation. For instance, higher content of nucleic acids has been described for inflammatory as well as tumorous regions [49]. Also, necrotic degradation may rather represent a tissue (transition) state than a tissue type, where molecular content might still resemble the original intact tissue type, especially in early stages (“pre‐necrosis”). Finally, variation was also introduced due to analysis on adjacent sections, which is accompanied by naturally changing tissue morphology, sectioning and slide mounting artefacts, as well as required co‐registration. The balanced accuracies, calculated for all samples combined, and also subject to all above mentioned deviations, to judge the overall performance of SA as a tissue type classifier including consideration of the prevalence of different TTOIs as compared to non‐TTOI regions, ranged from about 69% to 89% between all tissue types. Arguably, the number of fresh‐frozen CRLM samples with a short and controlled post‐surgery interval required for spatial lipidomics/metabolomics available for this study was limited. This needs to be taken into consideration for this proof‐of‐concept study. Results may differ, but may actually also be more robust in a mandatory full clinical follow‐up investigation. All in all, labels provided via computational SA analysis performed on MIR data were sufficiently accurate for subsequent correlative analysis of MSI data at spatial resolution, 40 µm obtained from the same tissue sections as MIR data to determine potential lipid marker candidates. This SA analysis on MIR imaging might be useful in disease areas other than cancer. For instance, other studies reported the successful MIR imaging‐based differentiation of ROI in multiple sclerosis patients or models [17, 18]. Here, either a clustering algorithm was used, which required a ground truth staining to select a suitable wavenumber range for the analysis and pathological input to assign clusters to tissue types of interest and define a suitable number of classes. Alternatively, even though wavenumber features specific to the tissue types of interest were reported, no analysis algorithm was used for the automatic annotation of these regions of interest. In both cases, the SA‐based analysis workflow proposed here could overcome these limitations. It should be noted that this SA‐based analysis may be useful even beyond diagnostic tissue classification, as reported spectral profiling methods suggest additional utility in pharmacology and drug research [52].

### 2.5 Correlated Multimodal MIR‐MS Imaging Reveals M/Z Features for Identification of Candidate CRLM Lipid Markers by on‐Tissue Iprm‐PASEF

**FIGURE 6:**
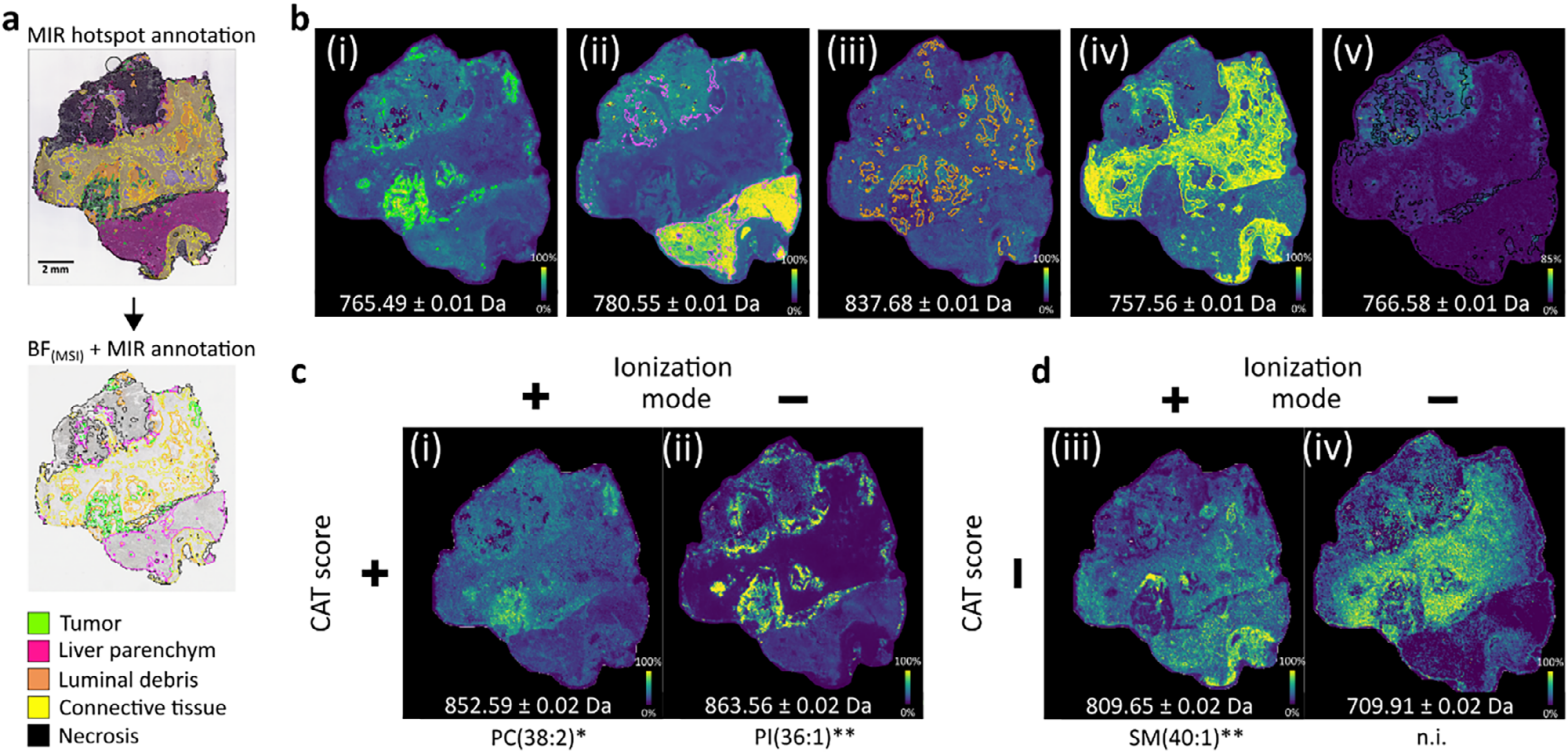
Correlative multimodal MIR and MALDI imaging for selection of cancer lesion‐specific *m/z* features, followed by their identification by iprm‐PASEF. (a) Tissue type annotations obtained by MIR imaging‐based SA analysis of all resected CRLM tissues (first set of six samples via cohort‐wide and second set of six samples reference‐based SA analysis) were co‐registered with the bright field image initially used for coordinate teaching during the MSI experiment (BF_(MSI)_). (b) Correlative multimodal MIR (defining ROIs outline in corresponding colors) and MALDI imaging revealed specific co‐localizing *m/z* features for all tissue types: (i) *m/z* 765.49 was characteristic for tumor, (ii) *m/z* 780.55 for liver parenchyma, (iii) *m/z* 837.68 for luminal debris, (iv) *m/z* 757.56 for connective tissue, and (v) *m/z* 766.58 for necrosis. c) & d) Discriminant analysis: Correlation‐adjusted t (CAT)‐scores utilizing MIR‐correlated BF_(MSI)_ annotations as labels were used to extract *m/z* features (positive or negative ionization mode) that distinguished the tumor regions from all other tissue types by displaying either increased (c) or decreased (d) ion intensity and corresponding positive or negative CAT scores, respectively: (i) *m/z* 852.59 ([PC(38:2)+K]^+^; Figure S31), (ii) *m/z* 863.56 ([PI(36:1)‐H]^−^; Figure S43), (iii) *m/z* 809.65 ([SM(40:1)+Na]^+^; Figure S33), (iv) *m/z* 709.91 (n.i. not identified). Iprm‐PASEF fragmentation analysis revealed *head group fragment, sum composition annotated via elimination of possible annotation candidates via LipidMaps database comparison, **head group fragment, both fatty acid side chains, and database comparison.

Tissue type labels computationally defined by SA analysis on MIR imaging data were utilized to annotate regions in all heterogeneous CRLM samples of twelve patients as tumorous, connective tissue, luminal debris, necrotic, or liver parenchyma. These annotations enabled spatially resolved discriminant analysis and the determination of *m/z* features via correlative MIR and MS imaging analysis. For this purpose, MIR and MSI datasets were co‐registered, aligning tissue type annotations across modalities (Figure 6a). In this study, MSI analysis was always conducted directly after completion of MIR imaging. However, sections may also be refrozen and measured later to meet potential requirements in clinical scenarios. While freezing is not known to change the molecular composition of samples, morphological changes through ice crystal formation can be caused upon slow freezing. Therefore, it is recommended that sections be dried in a desiccator and be stored in vacuum‐sealed bags to prepare for refreezing. After MSI measurements in both ionization modes and data pre‐processing, about 6,000 peaks were picked for further analysis (Figure S30). *m/z* features with ion intensities that were selectively increased in only one tissue type were observed for all annotated tissue types (Figure 6b). Given the much higher biomedical interest in tumor markers than in markers of any other tissue type, discriminant analysis using correlation‐adjusted t (CAT) scoring was then utilized to select discriminant features specifically for the tumorous regions [53]. 257 *m/z* features remained after individual analysis of all samples. This analysis was performed for each sample individually to retain features in this first step only observed in individual patient samples, while reducing the list of thousands of measured *m/z* features to the most promising features so that significance testing could be performed. Here, significance testing was conducted to judge the relevance of determined features for the entire cohort, which are therefore arguably more likely to have biomedical meaning. Here, Wilcoxon‐rank sum test was used to compare the mean spectra of patients (n = 12) of any non‐tumor tissue type with tumor individually. Features which passed a 5% confidence level in every comparison, resulted in a list of 15 *m/z* features with significant discriminant values, which could be subdivided into four groups by ionization mode and positive or negative correlative value (Figure 6c,d). 10 additional *m/z* features were annotated on the MS1 level in at least half of the patients (database comparison using MetaSpace). They were manually added, in order to expand the *m/z* feature list intended for molecular annotation by on‐tissue MS2 fragmentation analysis by iprm‐PASEF for spatial lipidomics [17].

**FIGURE 7:**
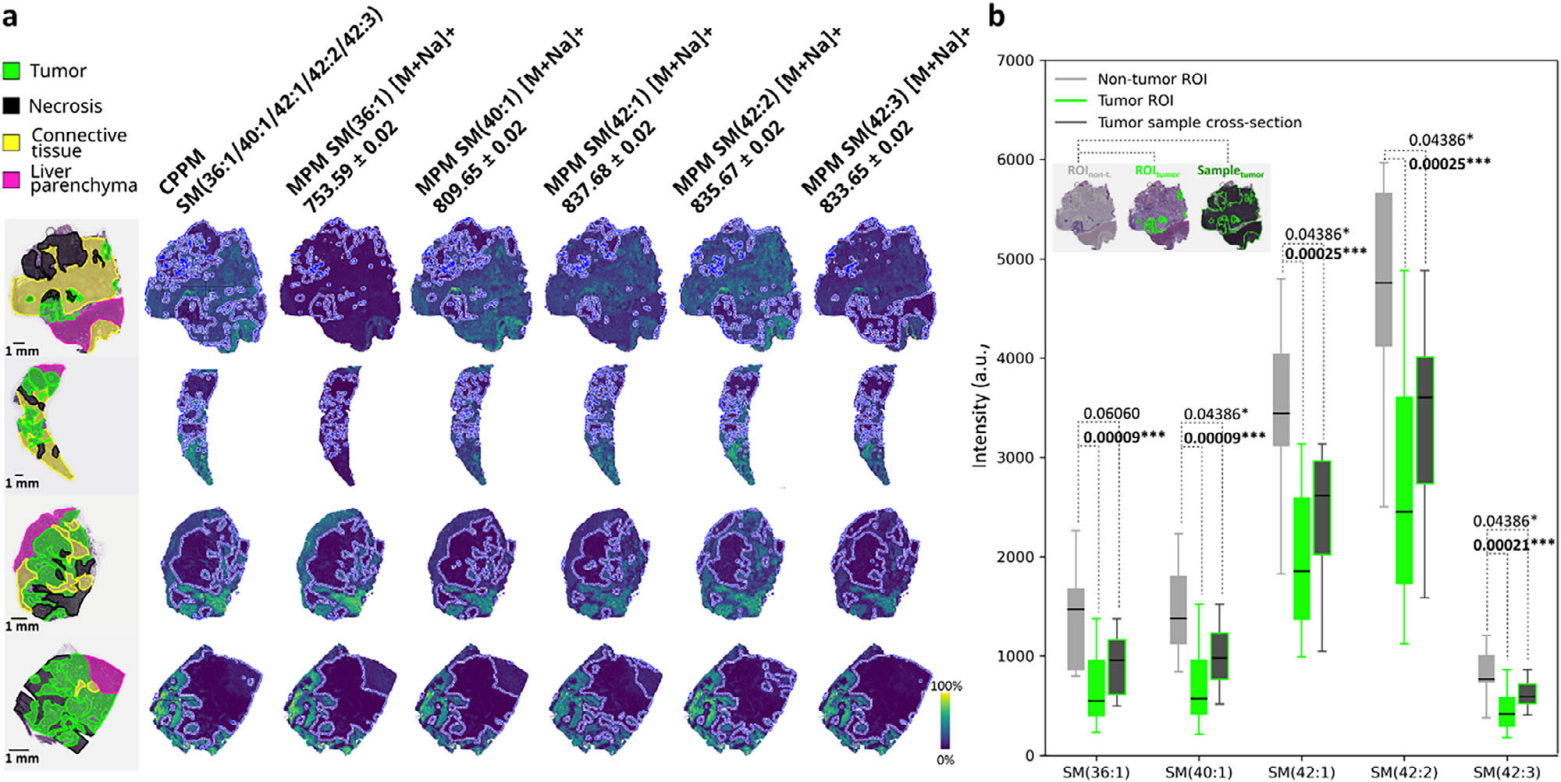
Tumor‐specific decrease of long chain sphingomyelin species in CRLM. (a) Molecular probabilistic maps (MPMs) and their collective projection probability map (CPPM) indicated statistically significant lower presence of five long chain sphingomyelin species (SM(36:1), *m/z* 753.59; SM(40:1), *m/z* 809.65; SM(42:1), *m/z* 837.68; SM(42:2), *m/z* 835.67; SM(42:3), *m/z* 833.65; all depicted here as [M+Na]^+^) in tumor regions than in other tissue types. For iprm‐PASEF MS2 spectra see Figures S33–S38 and Table S3. Scale bars 1 mm. (b) Ion intensities (RMS normalized) of these five SM lipids were significantly different between tumor (green) and non‐tumor (gray) ROIs, whereas low to no significant differences were observed between non‐tumor ROIs and the full tumor tissue section (black with green outline). For significance testing a Wilcoxon‐rank‐sum test (Benjamini‐Hochberg corrected) was performed with n = 12 patient means of the classes (exemplified below the legend), significance levels *0.05, **0.01, ***0.001 are indicated, boxplots were based on the mean intensities of n = 12 patients, box ranges the lower to upper quartile with the median represented as black line, whiskers extend to the furthest data point within the 1.5x inter‐quartile‐range.

Trapped ion mobility spectrometry (tims) analysis revealed two isobaric molecules at *m/z* 281.2486 (1/K_0_ = 0.8 Vs/cm^2^ and 1.0 Vs/cm^2^). Therefore, the final list for iprm‐PASEF analysis contained 26 *m/z* features (Table S3). 13 of these were sufficiently abundant and free of proximal interfering peaks in the isolation window to be analyzed by iprm‐PASEF directly on tissue (Table S3m/z and Figures S31–S43). Several identified lipids such as the phosphatidylcholine PC(34:1) have been frequently described in different cancer types like hepatocellular carcinoma [54]. Moreover, relative spatial abundance of five long chain sphingomyelin (SM) species (SM(36:1), SM(40:1), SM(42:1), SM(42:2), and SM(42:3)) was significantly decreased in tumorous vs. non‐tumor regions, as indicated by molecular probabilistic mapping (Figure 7a) [27]. Three of these, (SM(36:1), SM(40:1), and SM(42:1) out of these five), had previously been found to be significantly altered in homogenates from colorectal cancer tissue samples, as assessed by LC‐MS [55]. Since colorectal cancers often are exceptionally heterogeneous, which is neglected in tissue homogenates, significance in LC‐MS experiments likely depends on the amount of tumor cells present in each sample, which can vary drastically. As a point in case, the comparison between purely non‐tumorous ROIs vs. tumorous ROIs (defined by MIR hotspot annotation) as well as non‐tumorous ROIs vs. the entire tumor tissue sample (whole cross‐section disregarding tissue type composition and thus tumor sample heterogeneity) showcased the decreased level of significance in the latter (Figure 7b). SM isoforms are part of cell membranes, often serving as anchor for proteins connected to signaling or membrane transfer processes, that are commonly altered in tumorous tissues [31, 32]. These findings highlight lipid alterations in the membrane bilayer as a promising area for further investigation, with the potential to uncover key metabolic interactions and their roles in disease development and progression. It has been known for some time that colorectal cancer pathogenesis depends on the sphingolipid balance in colon epithelial cells, in particular on ceramides and their derivatives like SM, and that colon cancer may be mitigated by therapeutic intervention with sphingolipid metabolism [56, 57]. Interestingly, industrial pharmaceutical research using 3D tumor cell models under hypoxic conditions for screening, identified the marketed phenothiazine antipsychotic Fluphenazine as a functional acid sphingomyelinase inhibitor that caused cellular sphingomyelin accumulation and induced cancer cell death specifically in hypoxic tumor spheroids [58]. Consequently, repurposing of phenothiazines may be an option for future cancer treatment [59].

It should be noted that it was not surprising that lipids were the main tissue type‐defining feature identified in this study. In fact, lipid signatures and corresponding libraries are already used for in‐line tumor margin assessment in real time or near real time during surgical procedures using technologies like laser desorption ionization‐(LDI‐)MS, Rapid Evaporative Ionization Mass Spectrometry (REIMS)/“iKnife”, SpiderMass or MasSpec Pen technologies [60, 61, 62, 63].

While this full correlative MIR‐MSI‐iprm‐PASEF workflow (phases 1 to 6, Figure S5) showcased the successful selection of tumor‐specific marker candidates, it should be pointed out very clearly that the MIR‐only part of this procedure (phases 1 to 3, Figure S5; Figures 1, 2, 3, 4, 5) has merit and potential clinical utility on its own, since advanced MSI facilities will not be available everywhere: Interdependent SA mapping on MIR data—based on either cohort‐wide or reference‐based mapping—holds promise as an innovative platform for future use in clinical studies, since it requires little sample preparation, is label‐free and non‐destructive and uses easier‐to‐use equipment. This is even more true for recent QCL‐based MIR imaging microscopy that is up to 200‐fold faster than older FTIR imaging instrumentation used in this study [17]. As showcased in this study, MIR imaging‐based tissue typing models can be implemented already on a small number of samples (one patient sample used in this study) with good robustness against intra‐ and inter‐tumor heterogeneity, providing annotations for a larger number of samples. The code for this analysis is provided together with this study.

For larger patient cohorts it can be envisioned that in a clinical setting only some samples may eventually be analyzed by MSI using the full work flow, as they may have to be send to an external facility, whereas the MIR imaging could be on‐site. For MSI, iprm‐PASEF offers a recent option for ion mobility‐informed MS2 fragmentation analysis and candidate biomarker discovery directly on tissue. Beyond the correlative MIR‐MSI approach described in this study, tissue typing based on SA analysis in phases 1 to 3, could also be used in a QCL‐MIR imaging‐guided TIMS‐MSI approach, where MSI data acquisition is confined to distinct ROIs identified by MIR imaging [17].

Taken together, SA analysis was introduced for MIR imaging data to delineate and assign TTOIs based on pre‐selected wavenumber features, thus reducing the amount of ground truth data required for computational annotation. Furthermore, interdependent calculation of multiple samples using SA considerably improved the accuracy of annotation. Interdependent processing was implemented on the one hand as cohort‐wide SA, which was suitable for smaller cohorts and improved the annotation performance for homogenous samples. On the other hand, reference‐based SA analysis based on a previously calculated database offered interdependent calculation of larger cohorts without the requirement for advanced computing systems. It also allowed faster assessment of continuously added samples to a cohort over a longer period of time, which may be useful for‐ and perhaps even help reduce costs in some clinical trials. Both processing types could be employed in other imaging fields to improve performance of the analysis of homogenous samples. Using the computationally obtained MIR imaging‐based tissue type labels, specificity of correlative MALDI MSI was improved. Thus, correlative MIR imaging that delineates TTOIs based on their molecular composition could also be utilized for the analysis of other disease tissues to improve subsequent biomarker candidate analysis without the need for comprehensive and time‐consuming manual annotation requiring pathology expertise. In this study, correlative MIR‐assisted MSI imaging revealed several long chain sphingomyelin isoforms that were significantly decreased specifically in the tumorous regions, which could be further investigated for their biological contribution to the disease's progression, or their potential diagnostic or therapeutic suitability.

## 3 Conclusion

With this computational methodology we enable SA analysis on MIR imaging data for automated tissue type annotation of complete clinical tissue specimens based on wavenumber features selected using the permutation importance applied to a RF classifier. We introduce two procedures for interdependent SA analysis, one for analysis of entire patient cohorts, the other for incremental addition of new tissue samples to a reference tissue database. For a cohort of twelve CRLM patient samples, interdependent SA analysis performed equal to or better than manual pathology annotation—in part in a double‐blind study. Automated MIR imaging‐based tissue type labels can be integrated with MALDI‐MSI data to aid discovery of spatial biomarker candidates such as sphingomyelin isoforms by correlative multimodal imaging.

## 4 Experimental Section

### 4.1 Materials

2,5‐dihydroxy¬acetophenon (2,5‐DHAP, Thermo Fisher GmbH, Kandel, Germany), ACN (VWR Chemicals, Radnor, USA), dH2O was prepared in‐house using a MilliQ Refernace A+ water purification system (Merck KGaA, Darmstadt, Germany), Eosin Y‐solution 0.5 % aqueous (80 mL, Merck KGaA, Darmstadt, Germany), ESI‐L low‐concentration tuning mix (Agilent Technologies, Waldbronn, Germany), ethanol (VWR International GmbH, Darmstadt, Germany), Eukitt (Merck KGaA, Darmstadt, Germany), glass cover slip (VWR International GmbH, Darmstadt, Germany), HCl (Tritripur (1 mol/L), Merck KGaA, Darmstadt, Germany), hematoxylin (Merck KGaA, Darmstadt, Germany), ITO slides (8 – 12 Ohm resistance, Diamond Coatings Ltd., Brierley Hill, UK), MgSO4 (VWR Chemicals, Radnor, USA), NaHCO3 (Merck KGaA, Darmstadt, Germany), red phosphor (Merck KGaA, Darmstadt, Germany), SuperFrost Plus adhesion slides (VWR International GmbH, Darmstadt, Germany), TFA (Merck KGaA, Darmstadt, Germany), xylene (Thermo Fisher Scientific, Waltham, USA).

### 4.2 Cancer Tissue Specimens

Surgical tissue specimens were collected with informed consent of all patients at the Mannheim University Hospital in accordance to the Declaration of Helsinki with approval by the Medical Ethics Committee II of the Medical Faculty Mannheim of the Heidelberg University (2012‐293N‐MA, 20 June 2012). Tissue samples of twelve patients diagnosed with colorectal cancer liver metastases (CRLM), as well as control samples obtained from the proximity outside of the tumor nodule (obtained whenever possible) were retrieved (details in Table S4) and fresh‐frozen using dry ice (IDs 5, 7, 9, 10), or liquid nitrogen (IDs 18, 19, 21, 25, 27, 30, 31, 32) during surgery. When frozen, the samples were stored at ‐80 °C until further use. For cryo‐sectioning, samples were equilibration at ‐ 16 °C for at least 20 min in the cryostat (CM1860 UV, Leica Biosystems, Nussloch, Germany), after which cryo‐sections (10 µm section thickness) were obtained and thaw‐mounted on SuperFrost Plus adhesion slides for histological staining and ITO slides for MIR imaging, MSI, and iprm‐PASEF analysis (conducted subsequently on the same slide).

### 4.3 Histological Reference Staining and Pathological Annotation

Hematoxylin and eosin (H&E) staining was performed as follows: 2 min hematoxylin, 3 min tap water, 1 min acidic alcohol (350 mL ethanol + 150 mL dH_2_O + 1.5 mL HCl, 3 dips in dH_2_O, 2 min blueing solution (2 g NaHCO_3_ + 20 g MgSO_4_ in 1 L dH_2_O), 3 dips in dH_2_O, 2 min eosin (acidified, 80 mL with 0.2 mL HCl (1 mol/L)), 3 dips in dH_2_O, 1 min 80% ethanol, 2 min 96% ethanol, 1 min 100% ethanol, 1 min 100% ethanol, 2 min xylene, and covered with Eukitt and a glass cover slip. Optical images were recorded with an Aperio CS2 Scanner (objective: 20 × /0.75 NA Plan Apo, Leica Biosystems) and visualized using Aperio Image Scope (v. 12.4.6.5003, Leica Biosystems). Pathological annotations used for discriminant analysis on MIR imaging data for selection of wavenumbers specific to the corresponding tissue types were performed in SmartZoom (Smart In Media AG, Cologne, Germany) by expert pathologists and extracted via screenshot and subsequent color recognition. Pathological annotations used as accuracy measure to evaluate annotation performance of MIR tissue typing were performed in the open microscopy environment OMERO (v. 5.6.6) [64], where annotations could be exported as polygonal chains in .csv format.

### 4.4 Mid‐Infrared (MIR) Imaging and Pre‐Processing

Sections on ITO slides were equilibrated at room temperature for about 15 min, and MIR imaging was performed on an FT‐IR imaging system (Spotlight 400, Perkin Elmer LAS, Rodgau, Germany) as described elsewhere [20]. Briefly, MIR images were recorded with 25 µm lateral step size within a wavenumber range of 750 – 4000 cm^−1^ at 4 cm^−1^ spectral sampling interval, with 2 scans per pixel and 2.2 cm s^−1^ mirror speed. MIR data pre‐processing for later discriminant analysis was performed by common standards [65], including background normalization, second derivative and standard normal variate (SNV) normalization using an *in‐house* written Python (v. 3.8) script based on the libraries *numpy* (v. 1.22.3) [66], *matplotlib* (v. 3.5.1), *specio*, SciPy (v. 1.8.0) [67], and *os*. For simplicity, we report data obtained from spectral differentiation, usually given in units of 1/cm^−2^ (2^nd^ derivative), as unit‐less. MIR measurements were conducted on the same tissue sections directly before mass spectrometry imaging (MSI) analysis, since it had been previously established that no lipidomic changes could be observed in such a case [17]. Moreover, it was recently found in a correlative multimodal imaging study that stimulated Raman scattering (SRS) did not have an observable impact on subsequent MS images [68].

### 4.5 Mass Spectrometry Imaging (MSI) and Pre‐Processing

MSI measurements were conducted on a timsTOF flex mass spectrometer (Bruker Daltonics, Bremen, Germany) workflow as described elsewhere [17]. Briefly, slides were coated with a 2,5‐dihydroxyacetophenon (2,5‐DHAP) matrix (10 mg/mL) in 70% ACN/H_2_0 (v/v) with 0.1% TFA using a TM5‐Sprayer (HTX Technologies, Chapel Hill, USA) as follows: 10 layers at 100 µL/min flow; spray‐head velocity 1200 mm/min; 3 mm distance between the sprayed lines (HH pattern); spray nozzle height 40 mm; needle temperature 60 °C; table temperature 35 °C; pressure 10 psi; and gas flow rate 2 L min^−1^. For calibration, red phosphor was added as a standard after matrix application. MSI measurements were performed in positive and negative ionization mode on the same tissue sections in the range of *m/z* 100 – 2000 at a lateral step‐size of 40 µm, a spot size of 20 µm and a lateral offset between ionization modes of 20 µm in x‐ and y‐direction. Online calibration was performed using the ubiquitous endogenous ions phosphatidylcholine PC(36:4) (m/z 782.5694; [M+H]^+^) and phosphatidylinositol PI(38:4) (*m/z* 885.5499; [M‐H]^−^) in positive and negative ion modes, respectively. For each mass spectrum, ions from 200 shots at 10 kHz were accumulated at a laser power of 40%. Prior to analysis, the system was checked using the ESI source with the ESI‐L low‐concentration tuning mix and calibrated using the red phosphor standard in imaging mode. The *m/z* value search window was inferred from the mass resolution specific to a given measurement and device based on full width at the half‐maximum (FWHM; resolving power of 40k at *m/z* 1000) of the corresponding mass peak [27]. Pre‐processing of MSI data was performed using the R (v. 4.0.2) packages *MALDIquant* (v. 1.21) [69] and *moleculaR* (v. 0.9.1) [27] including spectrum‐wise intensity rescaling, where peak intensities were divided by the 95th percentile observed peak intensity, missing values were imputed to 0, mean intensity for each spectrum is taken as an intensity threshold to remove random noise peaks, peak binning (15 ppm), and a per‐group (tissue type) peak filtration was applied such that peaks that show up in less than 1% of the pixels for each tissue type (inferred from MIR tissue type annotation) are filtered out provided that they don't show up in more than 1% of pixels in any other tissue type. The resulting unified intensity matrix was saved as a .csv‐file for subsequent feature selection. For calculation of CPPM and MPMs, off‐tissue pixels were removed before the analysis in SCiLS Lab (2023a Pro, Bruker Daltonics) using a bisecting clustering algorithm judged against the co‐registered brightfield image, and subsequently exported as imzML for coldspot mapping using *moleculaR* [27].

### 4.6 Fragmentation Analysis by iprm‐PASEF

For molecular annotation of lipid species corresponding to *m/z* features, MS2 fragmentation analysis based on imaging parallel accumulation serial fragmentation (iprm‐PASEF) was performed [17]. Here, slides used for correlative MIR‐MSI analysis had been stored vacuum‐sealed at ‐80 °C until fragmentation analysis. First, a trapped ion mobility spectrometry (tims) MSI run (MS1) was conducted for both, positive and negative ionization mode to determine the isolation windows. Prior to analysis, the system was calibrated using the ESI source with the ESI‐L low‐concentration tuning mix (tims cell at *m/z* 622; 132 V). Analysis was performed on samples IDs 19, 21, 18a, which had shown the highest amounts of all features of the fragmentation list (Table S3, Supporting Information) in sub‐regions of the tissue with the highest intensities, using a laser energy of 55% or 45%, at a laser frequency of 10 kHz, with 200 shots, at laser application and lateral step‐sizes of 50 or 20 µm, respectively, in an *m/z* range 50 – 1350 or 250 – 1350, with a ion mobility range of 0.7 – 1.8 or 0.5 – 1.7 Vs cm^−2^, a ramp time of 400 ms, a quadrupole low mass of 50 or 200 Da, a quadrupole ion energy of 5 eV, at a collision energy of 10 eV, and collision RF of 350 or 400 Vpp, with a transfer time of 65 µs, and a pre‐pulse storage time of 5 µs for both positive and negative ionization mode, respectively. The mass resolving power based on FWHM was about 40k at *m/z* 1000. From this data, ion mobility values were retrieved for *m/z* features for the subsequent iprm‐PASEF run (MS^2^). Here, not all *m/z* features could be fragmented due to insufficient isolation/separation from dominant proximal peaks or due to insufficient abundance for fragmentation analysis. Measurement parameters used for the remaining *m/z* features (*m/z* value, mobility) were for positive ionization mode in‐tumor (852.5868, 1.499 / 739.4698, 1.374), for positive ionization mode in non‐tumor (809.6537, 1.507 / 753.5911, 1.457 / 837.6828, 1.532 / 835.6690, 1.516 / 833.6542, 1.503 / 776.5962, 1.470 / 849.6279, 1.509), and for negative ionization mode in‐tumor (281.2486, 0.8296 / 716.5237, 1.3052 / 835.5335, 1.413 / 863.5641, 1.4461). Otherwise, iprm‐PASEF parameters were used as specified for the MS^1^ run, except that the *m/z* range was 50 – 1350, with a quadrupole low mass of 50 Da, fragmentation energies extrapolated between 0.8 – 1.56 Vs cm^−2^ and 31.92 – 55.00 eV (45.00 eV for *m/z* features 695.45, 822.53, and 754.52) or 60 00 eV, with *m/z* isolation windows of ±1.5 Da (0.75 Da for *m/z* features 695.45, 822.53, and 754.52), and mobility isolation windows of ±0.005 Vs cm^−2^ for both positive or negative ionization mode.

### 4.7 Discriminant Wavenumber Feature Analysis and Calculation of Projection Images

For discriminant analysis and selection of suitable wavenumbers used for subsequent SA analysis, annotations of various tissue types (tumor, connective tissue, luminal debris, necrosis, inflammation, and liver parenchyma) were provided by expert pathologists on an adjacent H&E‐stained section of one sample (ID18) that contained all tissue types of interest. Annotations of different tissue types were extracted via color recognition from the image, and MIR imaging data was co‐registered with H&E as described before [16]: In short, a python script using the Python libraries SimpleITK and simpleElastix was implemented using advanced Mattes mutual information as a metric for the linear interpolator and b‐spline (2000 iterations) transformations to transform the H&E‐stained section and according pathological annotations (moving modality) to fit the MIR imaging data set (fixed modality, raw data image of wavenumber 1656 cm^−1^). Pathological annotations were used as labels for discriminant analysis performed using a random forest (RF) classifier as has been done in other studies on MIR imaging data [45]. Here, a subset of randomly picked 500 pixels per tissue type were investigated which were drawn and investigated by the RF classifier (*scikit‐learn* library (v. 1.0.2)) in ten replicates. Any feature ranking among the top ten at least once was investigated further via error exclusion test. Here, only features remained where at most 25% of pixels were falsely assigned to any tissue type as compared to the TTOI when divided by an optimal cut‐off (determined via minimum estimation by iterative cut‐off adaption). Remaining wavenumbers specific to each tissue type (tumor: (964, 1108, 1156) cm^−1^, connective tissue: (1296, 1200, 1280, 1220, 1216, 1564, 1232, 1300) cm^−1^, necrosis (1604, 1468, 1628, 1512, 1612, 1240, 1452, 1664, 1476, 1504, 1600, 1596, 1608, 1084) cm^−1^, luminal debris (1168, 1132, 2872, 1172, 1040, 1128, 1132) cm^−1^, and liver parenchyma (1740, 1744, 1136, 1756, 1760) cm^−1^) were used further. To increase discriminant power of the later univariate SA analysis, intensity values of these wavenumbers for each tissue type were projected into a single image, following published literature [27]. We discriminated between positive predictive features (ppf, where intensities were higher in the TTOI as compared to all other tissue types), which were summed up pixel‐wise, and negative predictive features (npf, i.e., intensities in the TTOI were lower than in all other tissue types) which were subtracted pixel‐wise, starting at a baseline offset of +10 to avoid negative results. Here, the intensity value of the projection image *P* at pixel position *k* can be expressed as

(1) $$P_{k}=10+\sum T_{k}^{i}-\;\sum T_{k}^{j}\;$$

where *T* represents the normalized second derivative of transmittance at pixel position *k* of all images representing positive predictive features *i*, and *j* representing negative predictive features. Thus, the entire projection image consisting of all pixels *k = 1, …, n* with *n* being the total number of pixels of an image is defined as *P*. This analysis performed with an in‐house Python script relies also on the libraries *numpy* (v. 1.22.3) [66], *matplotlib* (v. 3.5.1), *pillow* (v. 9.1.0), *csv* (v. 1.0), *scikit‐image* (v. 0.19.2) [70], *random*, *cv2* (v. 4.5.5), and *SciPy* (v. 1.8.0) [67].

### 4.8 Implementation of Spatial Autocorrelation (SA) Analysis

Background pixels obtained from off‐tissue regions of MIR images or projection images were masked (multiplying MIR images with binary mask image (off‐tissue = 0, on‐tissue = 1) produced via k‐means classifier (*k = 2*) utilizing scikit‐learn (v2.2.0) on raw MIR imaging data at wavenumber 1552 cm^−1^) and omitted from SA analysis. The implementation of local spatial autocorrelation was based on the local Moran's I statistic described by the indicator for spatial correlation *I* at position *i*

(2) $$I_{i}=\frac{\sum_{j=1}^{n}W_{ij}\left(x_{i}-\;\bar{x}\right)\;\left(x_{j}-\;\bar{x}\right)\;}{\frac{1}{n}\;\sum_{j=1}^{n}\left(x_{j}-\;\bar{x}\right)}$$

where the weight matrix *W(i,j)* defines the contiguity between observations (at site *i* and neighborhood *j*, *x_i_* represents the intensity value at position *i*, $\bar{x}$ the mean intensity value of all observations *n*, and *x_j_* the mean intensity value of the neighborhood (queen contiguity, taking into account the eight neighboring pixels of any pixel).

For any given observation (pixel) the observed Moran's I local spatial autocorrelation calculated from the value at that position (intensity) and its neighborhood is compared to the local autocorrelation at that position calculated for a series of random distributions (999 permutations) obtained from randomly shuffling all other observations except the observation currently investigated. A user‐defined confidence level (1%) is used to decide whether the observed local autocorrelation could be explained by a random distribution or is very unlikely to occur as such. Thus, the decision for each pixel is made specifically whether it belongs to a significant pattern of high intensity values or not, which results in a significance (hotspot) map for the image representing a binary image (1 belonging to a hotspot, 0 non‐significant). This workflow was implemented via an in‐house Python script utilizing the libraries *GeoPandas* (v. 0.9.0), and *PySAL esda* (v. 2.4.1) for local spatial autocorrelation. Furthermore, the work flow relies on the libraries *os*, *math*, *numpy* (v. 1.22.3) [66], *seaborn* (v. 0.11.2), *matplotlib* (v. 3.5.1), *pandas* (v. 1.4.2) and *PySAL* (v. 4.6.2). For MIR tissue typing, SA analysis was performed to determine the corresponding hotspot maps of each projection image for all CRLM samples calculated from wavenumbers specific to each tissue type (tumor, connective tissue, luminal debris, necrosis, and liver parenchyma). The cohort (n = 12) of patients diagnosed with CRLM was split into two groups of six samples each, where the first was assessed using cohort‐wide SA analysis and the second using reference‐based SA analysis (as detailed below).

### 4.9 Cohort‐Wide Processing of SA

To enable combined SA analysis of all samples of a cohort in context of each other, individual MIR images (masked projection images or wavenumber images as specified) of each sample were equalized in size by zero‐padding and subsequent computational stitching to a “super image” consisting of all individual images. Thus, during estimation of the random distribution of intensity values, pixels from any tissue were included. Moreover, zero‐values were omitted from the analysis. This resulted in a hotspot map super image consisting of all samples, which could subsequently be disassembled to the individual images of each sample for further correlative analysis. Samples (IDs 18a, 5a, 7b, 9, 10, 19) were calculated using this workflow.

### 4.10 Reference‐Based Processing of SA

To enable processing of larger cohorts or faster processing times, reference‐based SA was implemented and performed on individual MIR images (masked projection images or wavenumber images as specified). Here, a newly added sample was calculated alongside a subset (tested for one, two, and three references, two used for clinical cohort) of reference samples (multiple combination tested for the performance, highest and lowest hotspot‐to‐total tissue area used for clinical cohort) retrieved from the original cohort (used as database, calculated via cohort‐wide processing) to circumvent re‐calculating of the entire cohort including the new sample. To reference the new sample to the entire original cohort and not only the two reference samples, the original confidence level (*α*) was adapted in a way that the two reference samples resembled their original, cohort‐wide processed hotspot map. The best fit of the references was iteratively determined by their overlap determined using the intersection over union (IoU) and the mean adjusted confidence level (α*) of both was used for the calculation of the subset (newly added sample and two reference samples) adjusting the newly added sample automatically in accordance to the original cohort. The adjusted confidence level (α*) was determined as

(3) $$\alpha^{*}=\arg\max_{\alpha \in \left[\alpha_{min},\alpha_{max}\right]}\frac{1}{m}\sum_{i=1}^{m}\mathrm{IoU}\left(R_{i},{\hat{R}}_{i}\left(\alpha \right)\right)$$

where the original confidence level (*α*) used for calculation of the database (cohort‐wide SA) was adjusted in a way that the fit between the hotspot map of the reference samples calculated in a reference‐based approach (${\hat{R}}_{i}$, consisting of the newly added sample and two reference samples (*m*) entailing the highest and lowest hotspot‐to‐total tissue area) and the hotspot map calculated for the database (*R_i_*) were maximized for each reference sample (i=1,…,m) as judged by the intersection over union (*IoU*). The mean of adjusted confidence levels obtained via the reference samples (*m = 2*) was used for definition of the adjusted confidence level applied to the newly added sample. The adjustment process was conducted iteratively from 0.0005 (α_*min*_) to 0.5000 (α_*max*_) in steps of 0.0005. Samples (IDs 21, 25, 27, 30, 31, 32) were assessed using this workflow.

### 4.11 Performance Evaluation for MIR Tissue Type Annotations

Tissue type annotations (tumor, connective tissue, necrosis, and liver parenchyma) provided by expert pathological annotation on H&E‐stained adjacent sections (fixed modality) were co‐registered with MIR data (moving modality) using the above‐described workflow to benchmark the quality of MIR‐based tissue type annotations calculated via SA analysis. Three equally sized rectangles per section were pathologically annotated, which were then compared to the same region extracted from MIR‐tissue typing results. The quality assessment of annotations was determined for each tissue type individually and was based on the accuracy providing information on the overall correctness of annotations including positive as well as negative annotations. The standard accuracy (stnd. acc.) described as

(4) $$stnd.\;acc.=\frac{TP+TN}{TP+TN+FP+FN}$$

where the percentage of correctly assigned pixels independent from the TTOI prevalence was calculated based on the assignment of true positive (TP), true negative (TN), false positive (FP), and false negative (FN) values as compared to pathological annotation, which was used to determine discrepancies between tissue type annotations of both modalities.

The Balanced Accuracy (BA) Determined as

(5) $$BA=\frac{TPR+TNR}{2}=\frac{\frac{TP}{TP+FN}+\frac{TN}{TN+FP}}{2}$$

where the percentage of correctly assigned classes dependent on the TTOI prevalence was calculated based on the assignment of true positive (TP), true negative (TN), false positive (FP), and false negative (FN) values as compared to pathological annotation, which determined the true positive rate (TPR, sensitivity), as well as the true negative rate (TNR, specificity). This metric was calculated from all samples for each tissue type and provided as an overall performance metric for SA analysis used for tissue type annotation based on MIR imaging data.

Furthermore, the precision (positive predictive value, PPV) calculated as

(6) $$PPV=\frac{TP}{TP+FP}$$

where the percentage of correctly assigned TTOI pixels, describing the correctness of all positive annotations (only calculated for tumor due to presence in all rectangles) based on the assignment of true positive (TP), true negative (TN), false positive (FP), and false negative (FN) values as compared to pathological annotation was provided as an additional metric.

The samples analyzed via reference‐based SA were additionally double‐blinded during the annotation process and only revealed once all annotations, MIR‐based as well as histopathological, were completed independently (ensured by an independent contributor).

### 4.12 Multimodal Data Integration (MIR‐MSI) and *m/z* Feature Selection Workflow

Tissue type annotation provided by MIR‐based SA analysis were used as labels for correlated MSI discriminant analysis to determine *m/z* features specific to the tumorous region. First, co‐registration between both modalities was conducted as detailed before. Here, MIR‐based tissue type annotations (moving modality, raw data image of wavenumber 1656 cm^−1^) were correlated with MSI results via the bright field (BF) image (fixed modality) also used for teaching during the MSI data acquisition. Subsequently, the co‐registered MIR‐based tissue type annotation images were transferred to the total size of the original teaching image (size of ITO slide holding multiple patient samples) by zero‐padding, performed for each map and each section individually. Implementation of this workflow was conducted via a python script relying on the libraries *numpy* (v. 1.22.3) [66], *matplotlib* (v. 3.5.1), *seaborn* (v. 0.11.2), *pillow* (v. 9.1.0), *scikit‐image* (v. 0.19.2) [70], *SciPy* (v. 1.8.0) [67], *tiffile* (v. 2022.4.8), *SimpleITK* and *simpleElastix*. In the next step, these binary reference maps in the size of the original teaching image were incorporated in the MSI data files for subsequent molecular analysis. This process was implemented as an in‐house R script. Here, reference maps, exported as tiff‐files, were imported into R (v4.0.2) using the package *EBImage* (v. 4.23.0) [71]. The SCiLSLabClient (v. 6.2.114) was used to import the spatial transformation matrix that links the spatial coordinates of each pixel of the MSI measurement to the pixels of the BF image teaching file. Thus, the retrieved spatial transformation matrix was used to link MSI pixel coordinates to the spatial coordinates of the reference maps. Spectra, according to their pixel IDs, were identified using the package *spatstat* (v. 2.3.4). For subsequent discriminant analysis using the tissue type annotations (tumor, connective tissue, luminal debris, necrosis, and liver parenchyma) the identified MSI spectra were sampled (n = 500) per reference map and tissue section and exported into csv‐files. Additionally, the reference maps were also imported into the correspondding SCiLS files as labels using the SCiLSLabClient (v. 6.2.114) for visualization and statistical testing performed directly in SCiLS Lab. Correlative multimodal MIR‐MSI analysis investigating *m/z* features which are specific to tumorous regions as compared to all others was performed by reloading the pre‐processed MSI data into R (v. 4.0.2) and ranking the predictor *m/z* values by computing CAT scores (correlation‐adjusted t‐scores) [53] on a binary comparison basis between the tumor and every other tissue type individually via R package *sda* (v. 1.3.8). A cut‐off of local FDR (lfdr) < 0.2 was used to retain significant non‐null *m/z* values [72]. This procedure was performed for every patient sample individually to incorporate features which showed discrimination for only one patient and not disregard any features at an early stage. After filtering for monoisotopic masses, this resulted in four feature lists holding discriminant *m/z* values including positive ion mode and positive (45) and negative (65) predictive CAT values, as well as negative ion mode and positive (111) and negative (36) predictive CAT values which are in total 257 mass features. Subsequently, a funnel process was introduced to determine the most promising features representative of the entire cohort. Here, statistical significance testing was performed to determine significant and cohort‐wide trends of discriminant feature distribution. First, an Anderson‐Darling‐Test (Benjamini‐Hochberg‐corrected, 5% significance level) was conducted to test whether features were normally distributed. To be able and still include features where normal distribution could not be assumed, a Wilcoxon‐Rank‐Sum‐Test (Benjamini‐Hochberg corrected, 5% significance level) was applied with the number of samples being equal to the number of patients (n = 12) using the mean intensity per tissue type for the binary comparison (tumor against any other tissue type individually), all of which was performed in SCiLS Lab (2023a Pro, Bruker Daltonics). Remaining *m/z* features (total of 15 *m/z* features) were added to a fragmentation list for molecular annotation. Additionally, this list was appended by features which showed an initial discriminant distribution in the previously performed CAT scoring as well as could be annotated as a specific biological entity in at least half of the patients, thus being more promising for successful molecular annotation. Here, molecular annotation was performed using Metaspace (https://metaspace2020.eu/) and features which could be annotated within a *m/z* window of 0.1 Da using the SwissLipids database, which were found in the majority of samples, and spatially correlating with tumor regions in all samples were selected for fragmentation (total of 10 m/z features). All in all, this resulted in a list of 26 *m/z* features (two isobaric molecules at *m/z* 281.2486 (1/K_0_ = 0.8 and 1.0 Vs/cm^2^), chosen for subsequent fragmentation (Table S3, Supporting Information).

### 4.13 Molecular Annotation of Fragmentation Data

Recorded fragmentation data was loaded into Data Analysis (v. 6.1, Bruker Daltonics GmbH), where individual fragment spectra of each *m/z* feature were isolated from the iprm‐PASEF run and exported in mgf‐format. Subsequently, MS1 tims‐on data was loaded into Metaboscape (v. 2024, Bruker Daltonics) via the default lipidomics workflow. Afterward, MS^2^ spectra were added to the according *m/z* features and submitted to the rule‐based lipid annotation tool, as well as spectral library comparison for a first indication of the lipid class annotation. Ultimately, all fragment spectra were manually annotated based on chemical structure elucidation using ChemSketch (v. 2024.1.0, ACD/Labs Advanced Chemistry Development, Inc.), neutral loss analysis, and database comparison (LipidMaps).

### 4.14 Statistical Analysis

Hypothesis testing was performed to determine cohort‐wide significance of relative lipid abundance between tumorous and non‐tumorous tissue regions. Here, mass spectrometry imaging (MSI) data was initially pre‐processed using total ion count (TIC) normalization. Anderson‐Darling‐Testing (Benjamini‐Hochberg‐corrected, 5% significance level) showed that a normal distribution cannot be assumed for all m/z features. Thus, a Wilcoxon‐Rank‐Sum‐Test (two‐sided, Benjamini‐Hochberg correction for multiple testing, 5% significance level) was applied with the number of samples being equal to the number of patients (n = 12) using the mean intensity per tissue type for the multiple binary comparisons (tumor against any other tissue type individually). All of this was performed in SCiLS Lab (2023a Pro, Bruker Daltonics). Results of five significant *m/z* features exemplifying the comparison between tumor and non‐tumor regions of interest (ROIs), as well as the entire tumor sample cross‐section and non‐tumorous ROIs are presented as box plots based on the mean intensities of n = 12 patients with the boxes ranging from the lower to the upper quartile with the median represented as black line, and whiskers extending to the furthest data point within the 1.5x inter‐quartile‐range. Alpha values were displayed on top and resulting p‐values to judge significance levels are indicated by *0.05, **0.01, ***0.001.

### 4.15 Code Availability

Code and test dataset can be accessed on GitHub: https://github.com/CeMOS‐Mannheim/InSpIRe.

## Author Contributions

The study was conceived by M.F.R., S.S., and C.H. M.F.R. performed sample preparation, and FT‐IR and MSI measurements, designed experiments, implemented spatial autocorrelation analysis, analyzed data, prepared Figures, wrote python code and a first draft of the manuscript. M.F.R. and S.S. coordinated the work. N.E. implemented the reference‐based spatial autocorrelation analysis and performed data analysis. D.A.S. performed data analysis of MSI data using in‐house written R scripts; M.F.R. and B.F. performed fragmentation analysis via iprm‐PASEF; S.A.M. performed spatial clustering analysis and calculation of probabilistic maps; expertise in pathology, annotated tissue sections, and clinical tissue evaluation was provided by S.G. and C.‐A.W. for evaluation of MIR‐based tissue type annotation performance, and A.W. as ground truth labels for discriminant wavenumber feature selection; O.W. provided expertise and infrastructure for advanced analysis workflow implementation; S.S. provided code for co‐registration workflows and data import, and revised the manuscript; E.B. and N.N.R. performed surgical resections and provided human samples, in addition to assisting with clinical information and ethics approval; C.H. supervised and coordinated the overall work, evaluated the results, provided infrastructure, and wrote the final manuscript with input from all co‐authors. All authors have read and agreed to the published version of the manuscript.

## Conflicts of Interest

The authors declare no conflicts of interest.

## Supporting information




**Supporting File**: advs74306‐sup‐0001‐SuppMat.pdf

## Data Availability

All raw data supporting the findings of this study, including mid‐infrared (MIR) imaging data, mass spectrometry imaging data, and hematoxylin and eosin (H&E) staining data, are publicly available on https://doi.org/10.5281/zenodo.18495815.
