# Supplementary material for: Cohort‐Scale Spatial Autocorrelation for Tumor Prediction in Mid‐Infrared Pathology and Spatial Biomarker Discovery Using MALDI Imaging Lipidomics
Source: Adv Sci (Weinh). 2026 Feb 9;13(24):e16847. doi: 10.1002/advs.202516847 (PMC13115961; doi:10.1002/advs.202516847)
Supplement: Supplementary file 1 — Supporting File: advs74306‐sup‐0001‐SuppMat.pdf [file ADVS-13-e16847-s001.pdf]

Supporting Information

Cohort-scale Spatial Autocorrelation for Tumor Prediction in Mid-infrared Pathology and Spatial Biomarker Discovery using MALDI Imaging Lipidomics

Miriam F. Rittel, Nikolas Ebert, Denis Abu Sammour, Sebastian Graf, Björn C. Fröhlich, Emrullah Birgin, Shad A. Mohammed, Nuh N. Rahbari, Axel Wellmann, Oliver Wasenmüller, Cleo-Aron Weis, Stefan Schmidt, Carsten Hopf

---

Table of Contents

Supplementary Figures 1

Figure S1. Definition of spatially restricted ROIs in MALDI MS imaging (MSI) improves selection of *m/z* features that differentiate between heterogeneous colorectal cancer liver metastasis (CRLM) and non-cancerous tissue types. 1

Figure S2. Selection process for mid-infrared (MIR) imaging wavenumbers specific for tissue types of interest such as “tumor”. 2

Figure S3. Selection process showcased for subset of wavenumber features discriminative for tumorous tissue type 3

Figure S4. Discriminant wavenumbers for each tissue type in CRLM. 4

Figure S5. Overview of sample and data flow between different analyses. 5

Figure S6. Multiple wavenumber features projected into a single plane exhibit increased discriminant power of distinguishing tumor (T) and non-tumor (NT) as compared to individual features. 6

Figure S7. Projection images of discriminant wavenumbers for all tissue types and patients in the clinical cohort of CRLM. 6

Figure S8. Spatial autocorrelation predicts tissue type annotations in less biased fashion than spatially aware clustering. 7

Figure S9. Spatial autocorrelation analysis performed on projection images of discriminant wavenumbers compensated for variance between datasets measured at different time points. 8

Figure S10. Variance in pre-processed MIR imaging data measured at different time points. 9

Figure S11. Cohort-wide processing of SA is limited by memory usage and computation time. 10

Figure S12. Setup of evaluation process for reference-based processing of SA. 11

Figure S13. Adjusted confidence levels in reference-based processing versus initially set confidence level for calculation of the database. 12

Figure S14. Performance of reference-based processing for negative control samples matches the cohort-wide processing and both outperform individual processing. 13

Figure S15. Only interdependent SA processing can relate multiple samples to each other and increase annotation accuracy.......... 14

Figure S16. Comparison between mid-infrared imaging-based hotspot annotation and pathological annotations for patient ID5a.......... 15

Figure S17. Comparison between mid-infrared imaging-based hotspot annotation and pathological annotations for patient ID7b.......... 16

Figure S18. Comparison between mid-infrared imaging-based hotspot annotation and pathological annotations for patient ID9.......... 17

Figure S19. Comparison between mid-infrared imaging-based hotspot annotation and pathological annotations for patient ID10.......... 18

Figure S20. Comparison between mid-infrared imaging-based hotspot annotation and pathological annotations for patient ID18a.......... 19

Figure S21. Comparison between mid-infrared imaging-based hotspot annotation and pathological annotations for patient ID19.......... 20

Figure S22. Comparison between mid-infrared imaging-based hotspot annotation and pathological annotations for patient ID21.......... 21

Figure S23. Comparison between mid-infrared imaging-based hotspot annotation and pathological annotations for patient ID25.......... 22

Figure S24. Comparison between mid-infrared imaging-based hotspot annotation and pathological annotations for patient ID27.......... 23

Figure S25. Comparison between mid-infrared imaging-based hotspot annotation and pathological annotations for patient ID30.......... 24

Figure S26. Comparison between mid-infrared imaging-based hotspot annotation and pathological annotations for patient ID31.......... 25

Figure 27. Comparison between mid-infrared imaging-based hotspot annotation and pathological annotations for patient ID32.......... 26

Figure S28. Choice of suitable performance metrics. 27

Figure S29. Common deviations of MIR based hotspot annotations from expert pathologist's annotations. 28

Figure S30. Schematic overview of the *m/z* feature selection process. 29

Figure S31. Fragmentation spectrum obtained via iprm-PASEF of *m/z* 852.58, annotated as PC(38:2).......... 27

Figure S32. Fragmentation spectrum obtained via iprm-PASEF of *m/z* 739.47, annotated as PC(34:1).......... 28

Figure S33. Fragmentation spectrum obtained via iprm-PASEF of *m/z* 809.64, annotated as SM(40:1).......... 29

Figure S34. Fragmentation spectrum obtained via iprm-PASEF of *m/z* 735.59, annotated as SM(36:1).......... 30

Figure S35. Fragmentation spectrum obtained via iprm-PASEF of *m/z* 837.68, annotated as SM(42:1).......... 31

Figure S36. Fragmentation spectrum obtained via iprm-PASEF of *m/z* 835.66, annotated as SM(42:2). 32

Figure S37. Fragmentation spectrum obtained via iprm-PASEF of *m/z* 833.65, annotated as SM(42:3). 33

Figure S38. Fragmentation spectrum obtained via iprm-PASEF of *m/z* 776.59, annotated as SM(42:2)*. 34

Figure S39. Fragmentation spectrum obtained via iprm-PASEF of *m/z* 849.62, annotated as SM(42:3). 35

Figure S40. Fragmentation spectrum obtained via iprm-PASEF of *m/z* 281.25 annotated as oleic acid. 36

Figure S41. Fragmentation spectrum obtained via iprm-PASEF of *m/z* 716.52, annotated as PE(34:1). 37

Figure S42. Fragmentation spectrum obtained via iprm-PASEF of *m/z* 835.54, annotated as PI(34:1). 38

Figure S43. Fragmentation spectrum obtained via iprm-PASEF of *m/z* 863.57, annotated as PI(36:1). 39

Supplementary Tables 40

Table S1: Comparison of different algorithms used for tissue morphology annotation revealing best compromise between performance and computational implementation requirements for spatial autocorrelation analysis. 40

Table S2. Random forest ranking and error testing for selection of wavenumbers that differentiate between tissue types 41

Table S3. Overview of molecular annotations assigned via on-tissue fragmentation analysis using iprm-PASEF. 42

Table S4. Human samples and metadata. Each sample was labelled with an internal pseudonymized ID that was used throughout this study. 43

Supplementary References 43

Supplementary Figures

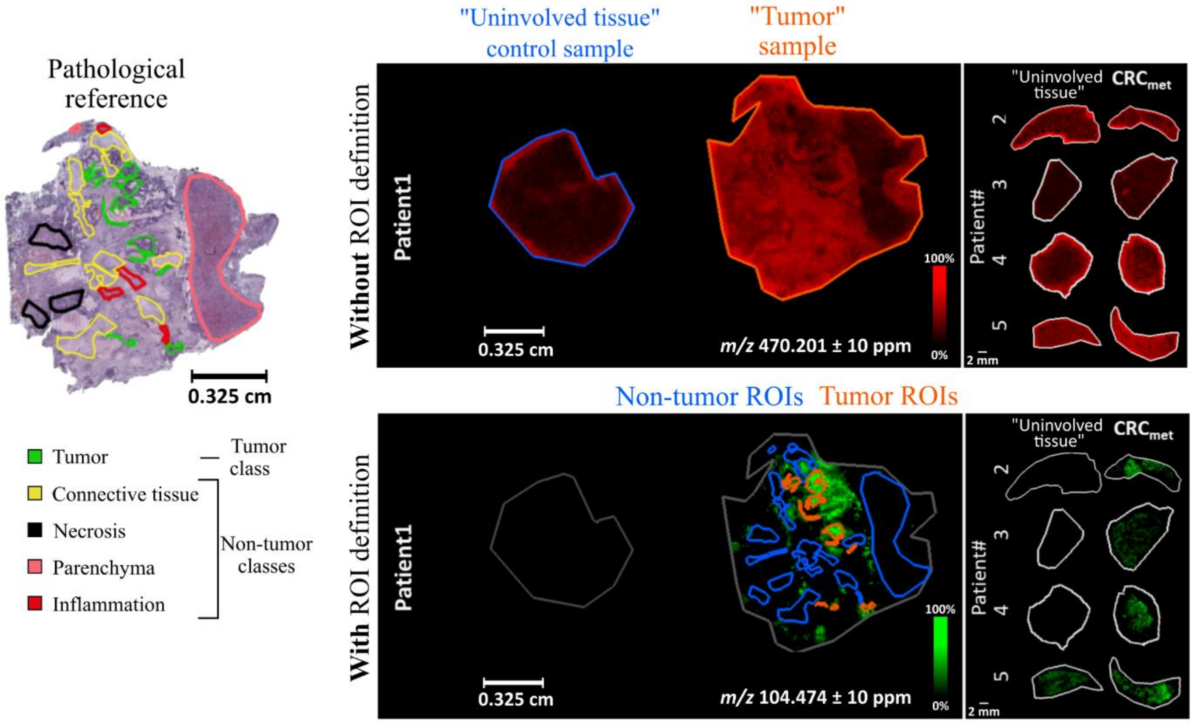

Figure S1. Definition of spatially restricted ROIs in MALDI MS imaging (MSI) improves selection of *m/z* features that differentiate between heterogeneous colorectal cancer liver metastasis (CRLM) and non-cancerous tissue types. Left: Reference regions of interest (ROIs) defined by an expert pathologist. Top right: FT-ICR MSI (obtained in positive ionization mode) and receiver operating characteristic (ROC) analysis of a full section of a human CRLM sample ("tumor") vs. full section of a control liver sample ("uninvolved tissue") of patient 1 revealed the MSI feature *m/z* 470.201, which did not translate well to resected tissues from patients 2 to 5. Bottom right: ROC analysis of tumor-specific versus non-tumor ROIs of the same patient 1 sample revealed *m/z* 104.474, which translated better to samples from patients 2 to 5. Note that "uninvolved tissue" control samples, usually retrieved from close proximity of the diseased tissue, might contain tumorous tissue (example right bottom, patient 5), thus potentially failing to capture relevant *m/z* features when comparing entire samples instead of ROIs.

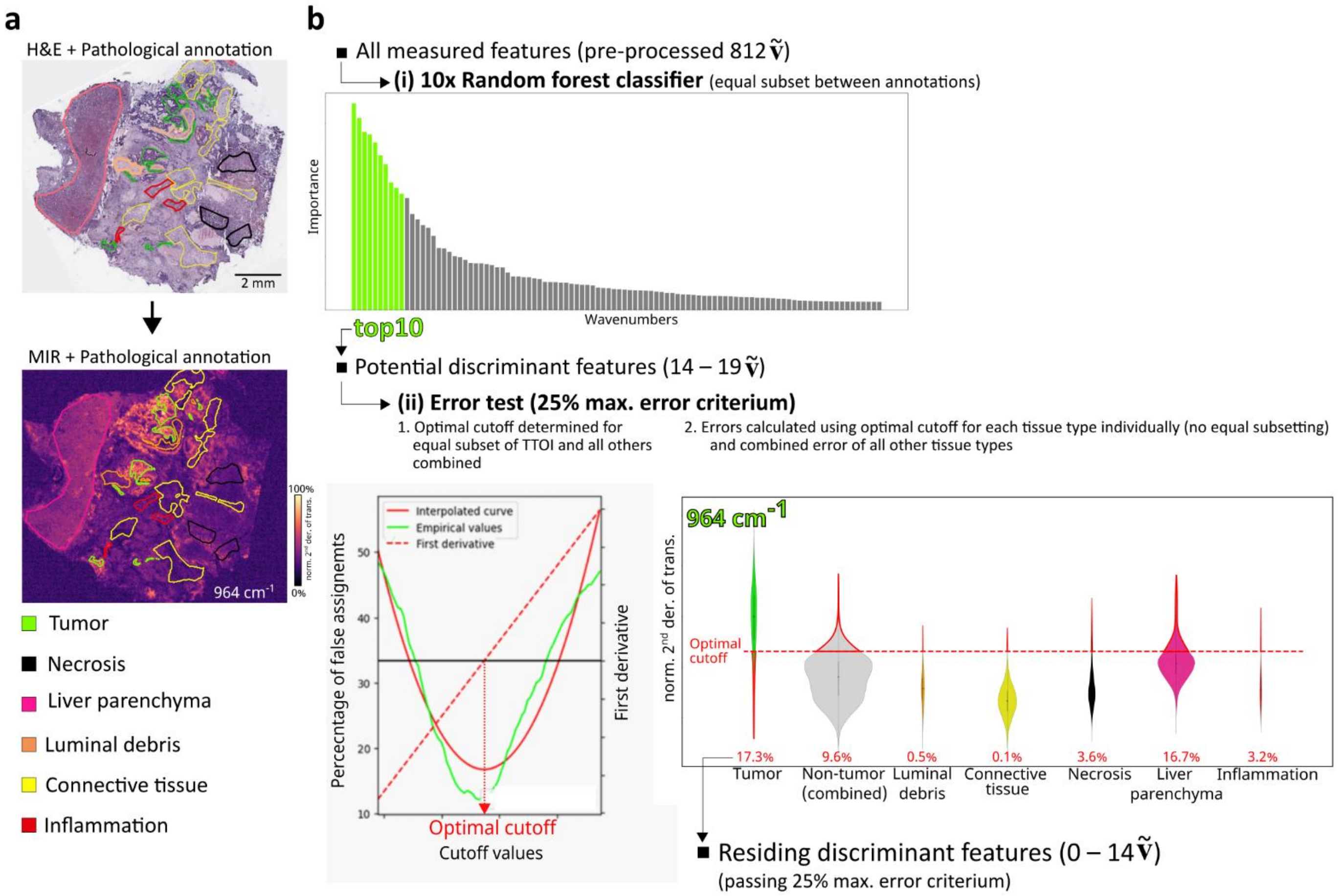

Figure S2. Selection process for mid-infrared (MIR) imaging wavenumbers specific for tissue types of interest such as "tumor". **a)** Pathological annotations for a single reference tumor tissue section were co-registered and transferred to MIR imaging data for pixel-by-pixel "ground truth" tissue class annotation used for wavenumber selection required for subsequent spatial autocorrelation (SA) analysis. **b)** Funnel process to determine wavenumbers that differentiate between tissue types: **(i)** All wavenumbers (812) were subjected to a **random forest ranking** based on a randomly selected subset of 500 pixels each per tissue type of interest (TTOI) and all other tissue types to determine their contribution to diagnostic differentiation. This ranking was repeated ten times, because of ranking variations due to randomly chosen pixels. Any wavenumber that ranked at least once (within the ten repetitions) among the top10 wavenumber features with the highest importance for differentiating between TTOI and all others combined were further investigated by **(ii)** an **error test**. An optimal cut-off between the TTOI and the other tissue types combined was determined via minimum determination of the total number of falsely annotated pixels (TTOI and others) plotted over iterative cut-off values (**1,** left). This cutoff value was then used to calculate the percentage of falsely annotated pixels for each tissue type individually (all pixels of that tissue type) as well as the total error (percentile of all falsely annotated pixels of all tissue types), and the combined error of all other tissue types (percentile of falsely annotated pixels of all tissue types combined except the one under investigation) (**2,** right). 25% was set as max. acceptable error and only wavenumbers passing this criterion for any tissue type or combination tested resided for subsequent SA analysis.

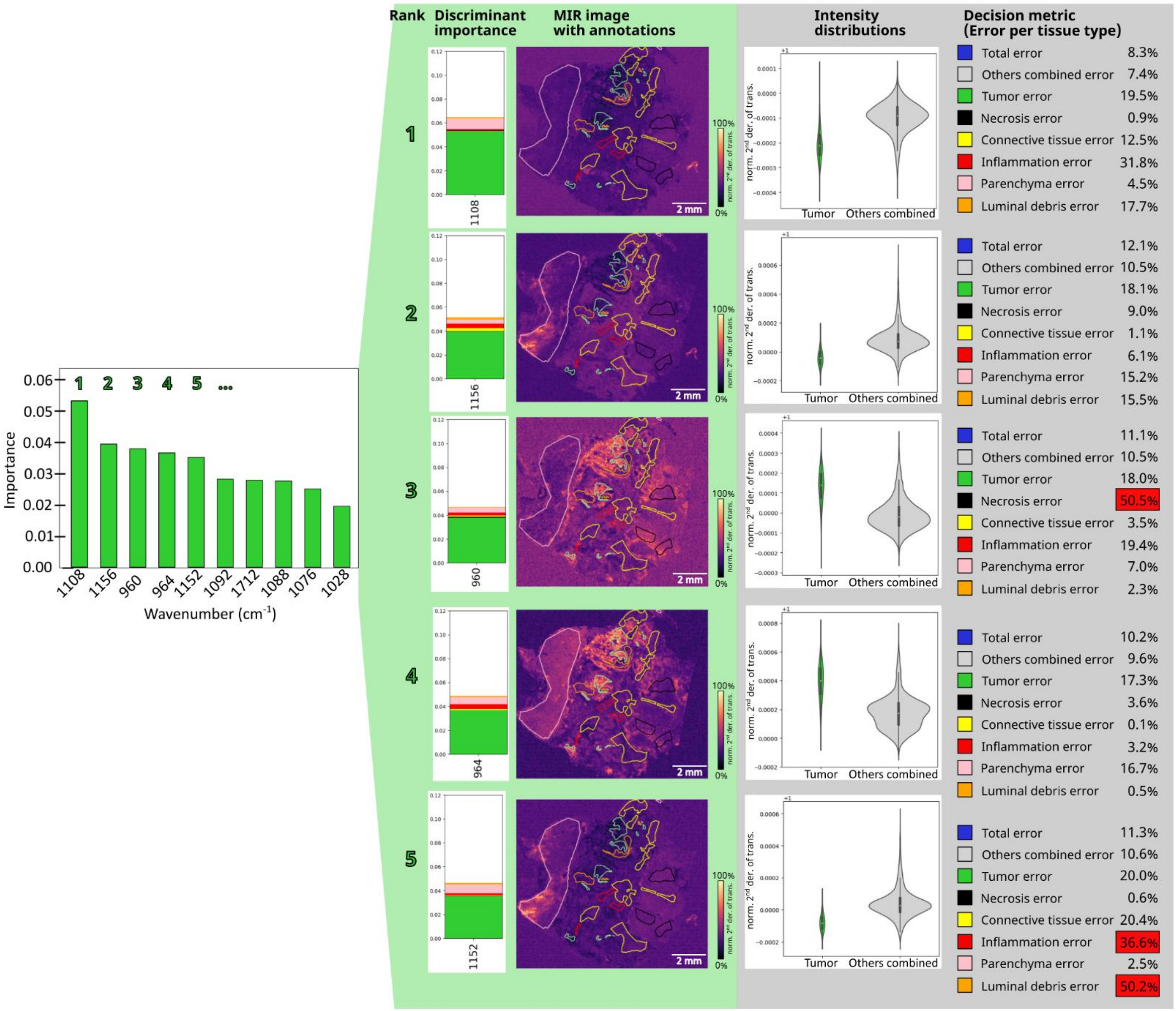

Figure S3. Selection process showcased for subset of wavenumber features discriminative for tumorous tissue type. Example for top10 wavenumber features for "tumor" in one iteration (out of 10) of random forest classifier. "Importance" is the metric of the Mean Decrease Impurity used by the random forest algorithm to describe the contribution of any feature to the discrimination between the classes or in other words by how much including a feature increases the accuracy. These values are unit-less and are normalized so that the sum of all equals 1. Example for error test of the top5 wavenumber features differentiating "tumor" from all other tissue types (red label indicated failing of the 25% criterion). This procedure was performed for all tissue types.

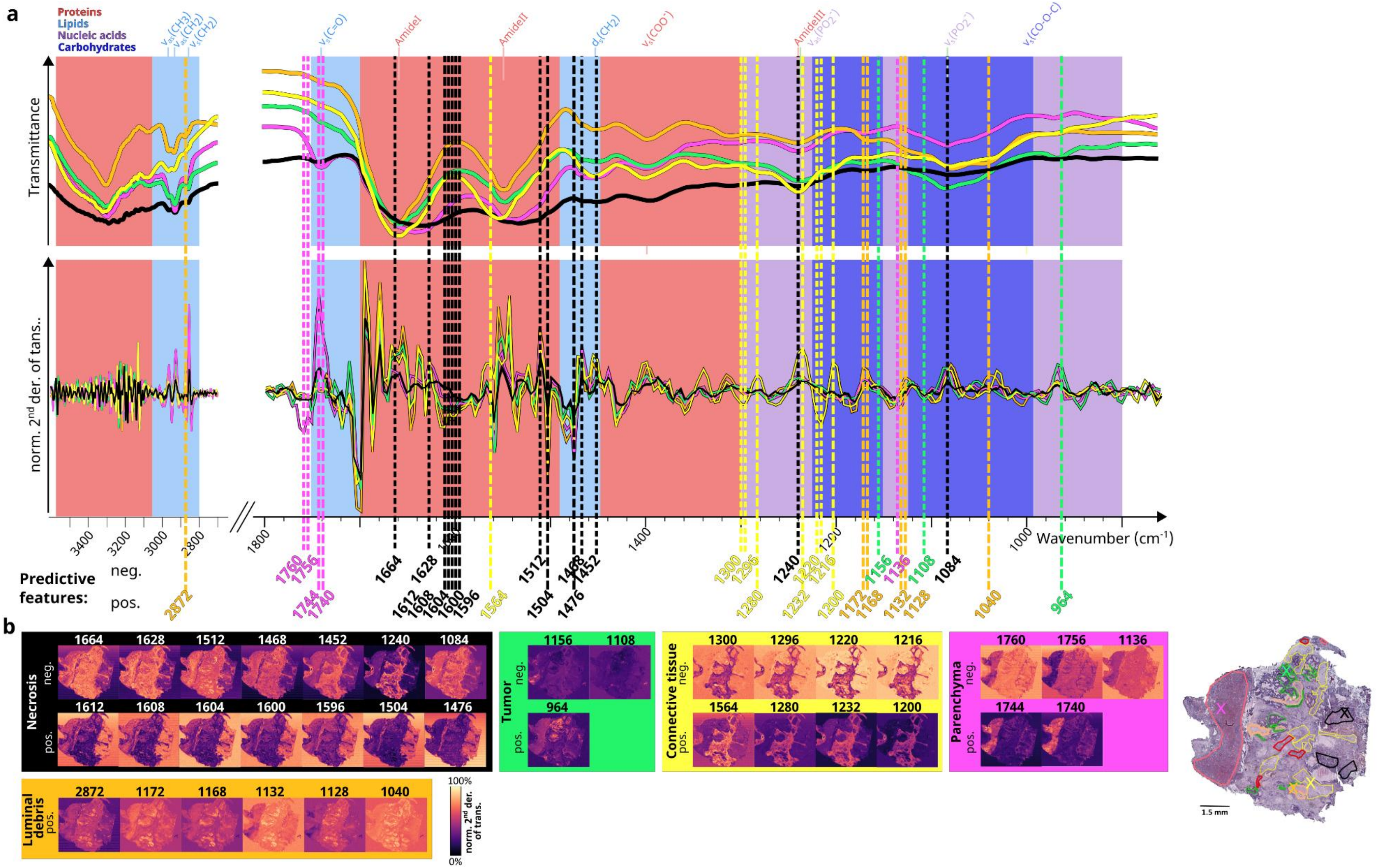

Figure S4. Discriminant wavenumbers for each tissue type in CRLM. **a)** Representative raw (top row) and pre-processed (bottom row) MIR spectra for each tissue type referenced against the four major biomolecular classes (proteins (red), lipids (blue), nucleic acids (green), carbohydrates (yellow)) based on Lovergne et al.[1]. **b)** MIR images of each selected wavenumber and their predictive value for the corresponding tissue type in accordance to pathologically annotation (the x in the tissue insert mark the approximate position of the extracted spectra presented in a).

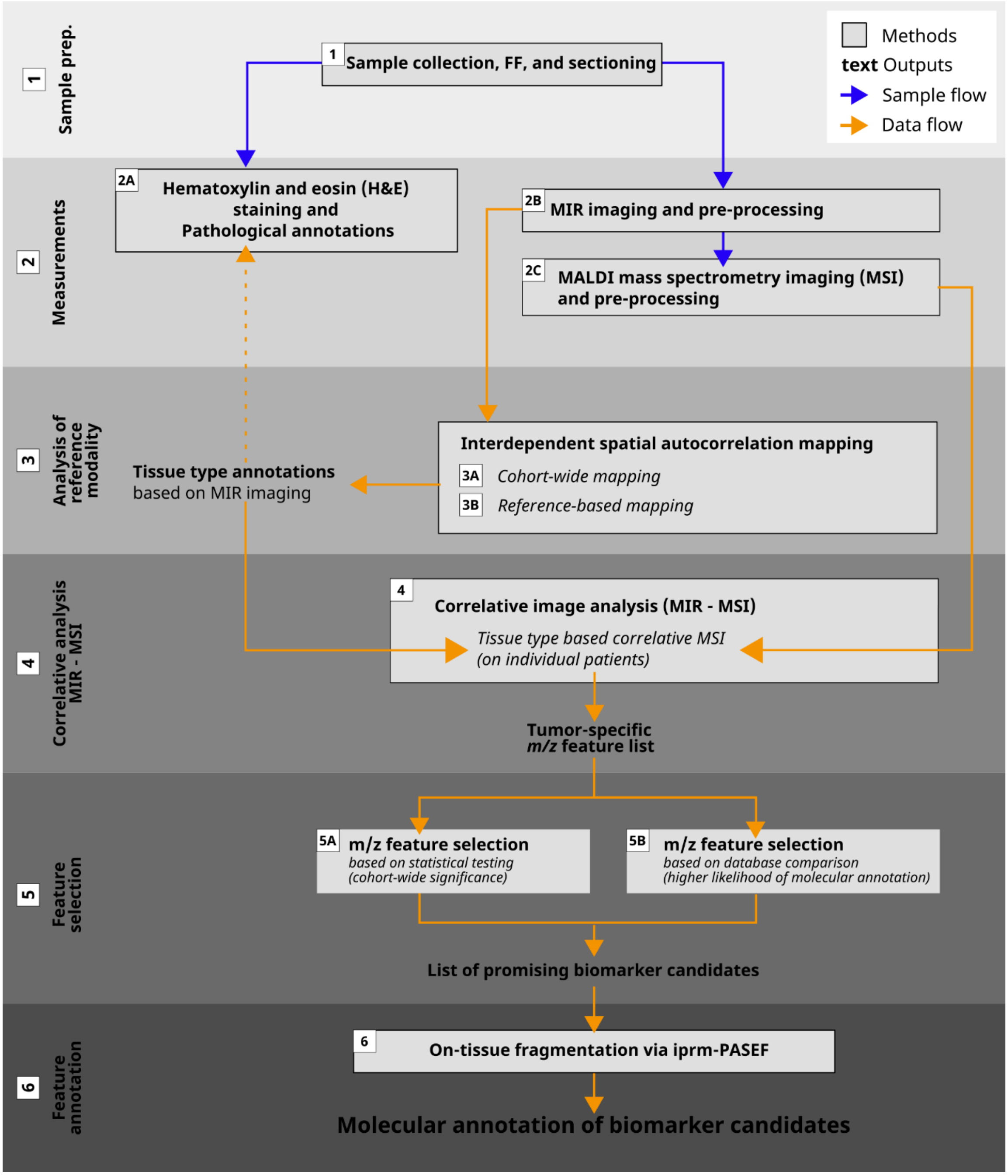

Figure S5. Overview of sample and data flow between different analyses. Process (top to bottom) divided into several workflow phases (1 – 6): Following sample preparation **(1)**, two sections (blue arrows) were subjected to different measurement modalities **(2A – C)**, from which MIR imaging data (orange arrow) was subjected to both variants of interdependent spatial autocorrelation analysis to define regions of interest delineating different tissue types within a tumor sample **(3)**. These were evaluated based on pathological annotations (dotted arrow), and utilized for subsequent correlative mass spectrometry imaging (MSI) **(4)**. This analysis determined *m/z* features specific for tumor cells tested for each patient individually. Subsequently, this feature list was reduced to *m/z* values with high likelihood of representing biological variance (testing for cohort-wide significance), and molecular annotation (molecular database comparison) **(5)**. The remaining *m/z* features were subjected to on-tissue fragmentation analysis for molecular annotation to determine potential molecular biomarker candidates **(6)**.

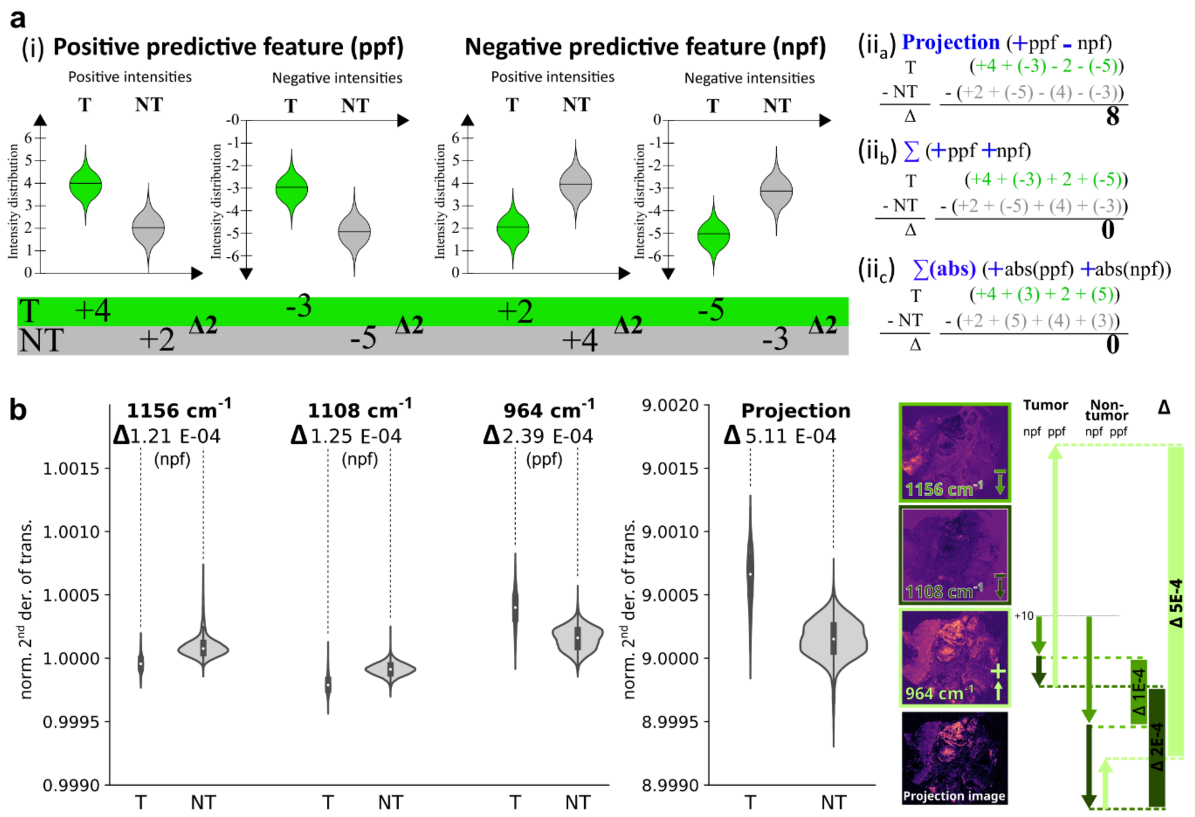

Figure S6. Multiple wavenumber features projected into a single plane exhibit increased discriminant power of distinguishing tumor (T) and non-tumor (NT) as compared to individual features. **a)** Schematic representation of positive (ppf) and negative predictive wavenumber features (npf) with (i) individual discriminant powers of (Δ2) between two imagined pixels of the corresponding tissue types. For both predictive feature directions (ppf and npf), positive and negative intensity values are considered (observed when using a derivative in MIR pre-processing). Exemplary calculation (ii) suggests that projecting wavenumber features into one plane by pixel-wise summation of ppf and subtraction of npf the discriminant power increases (**ii$_a$,** Δ8) between tumor (T) and non-tumor (NT) no matter if the intensity values are above or below zero. In contrast, summation of both (ppf and npf) results in decreased discriminant power when including npf where the intensity values are above zero (**ii$_b$,** Δ0), and summation of the absolute intensity values of both (ppf and npf) would result in decreased discriminant power when including ppf, where the values are below zero (**ii$_c$,** Δ0). **b)** Experimental data example of the projection of three predictive wavenumber features for tumor tissue regions (npfs: 1156 cm^{-1}, 1108 cm^{-1}, ppf: 964 cm^{-1}). Individual discriminative powers ((1.21, 1.25, 2.39) E-04, respectively) increase to 5.11 E-04 when combined via summation of ppfs and subtraction of npfs (*i.e.,* projection).

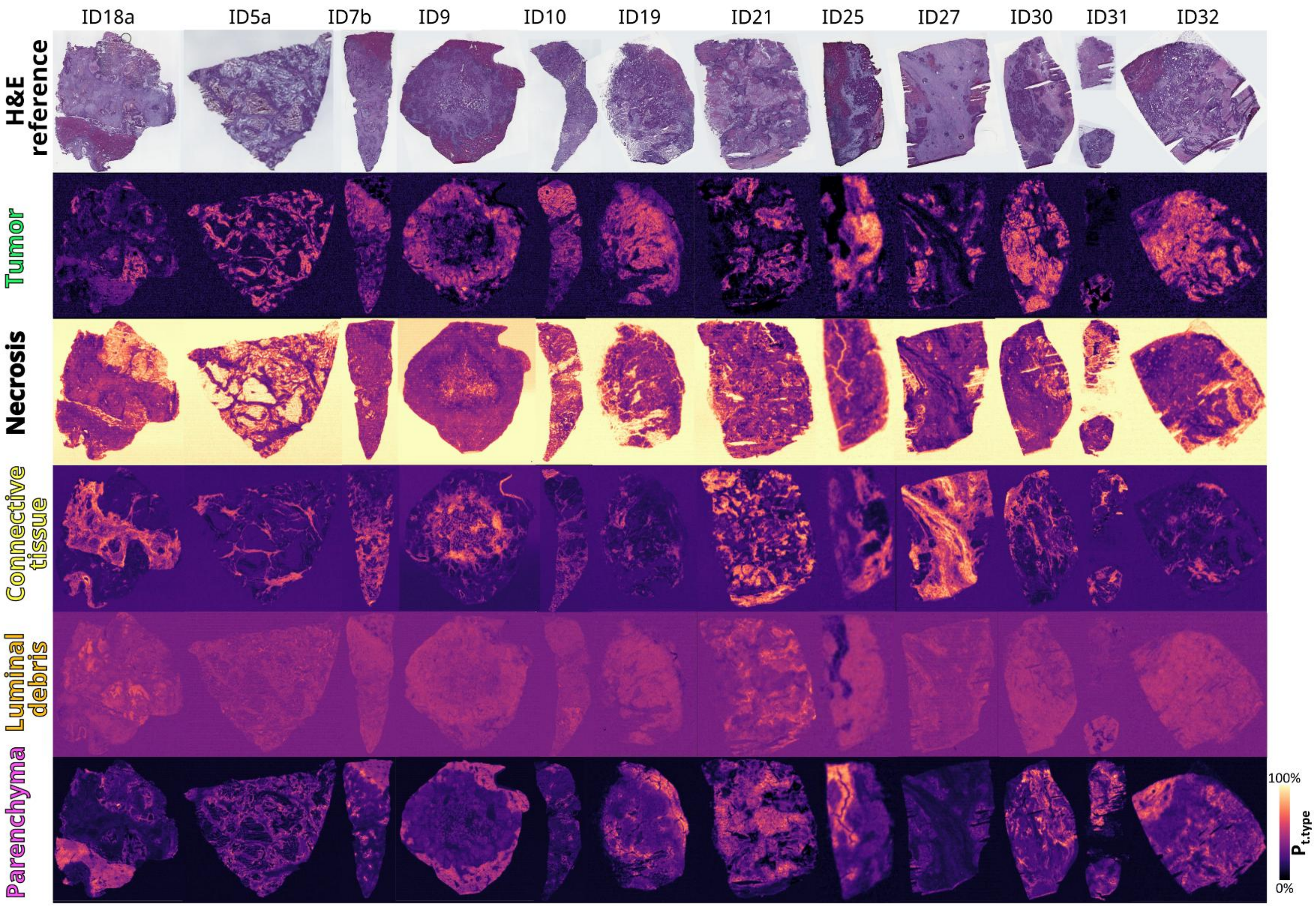

Figure S7. Projection images of discriminant wavenumbers for all tissue types and patients in the clinical cohort of CRLM. H&E-stained adjacent section provided as a reference (top row) for the projection images of all twelve patients (columns) calculated for all differentiable tissue types (rows) with the wave-numbers selected via discriminant analysis.

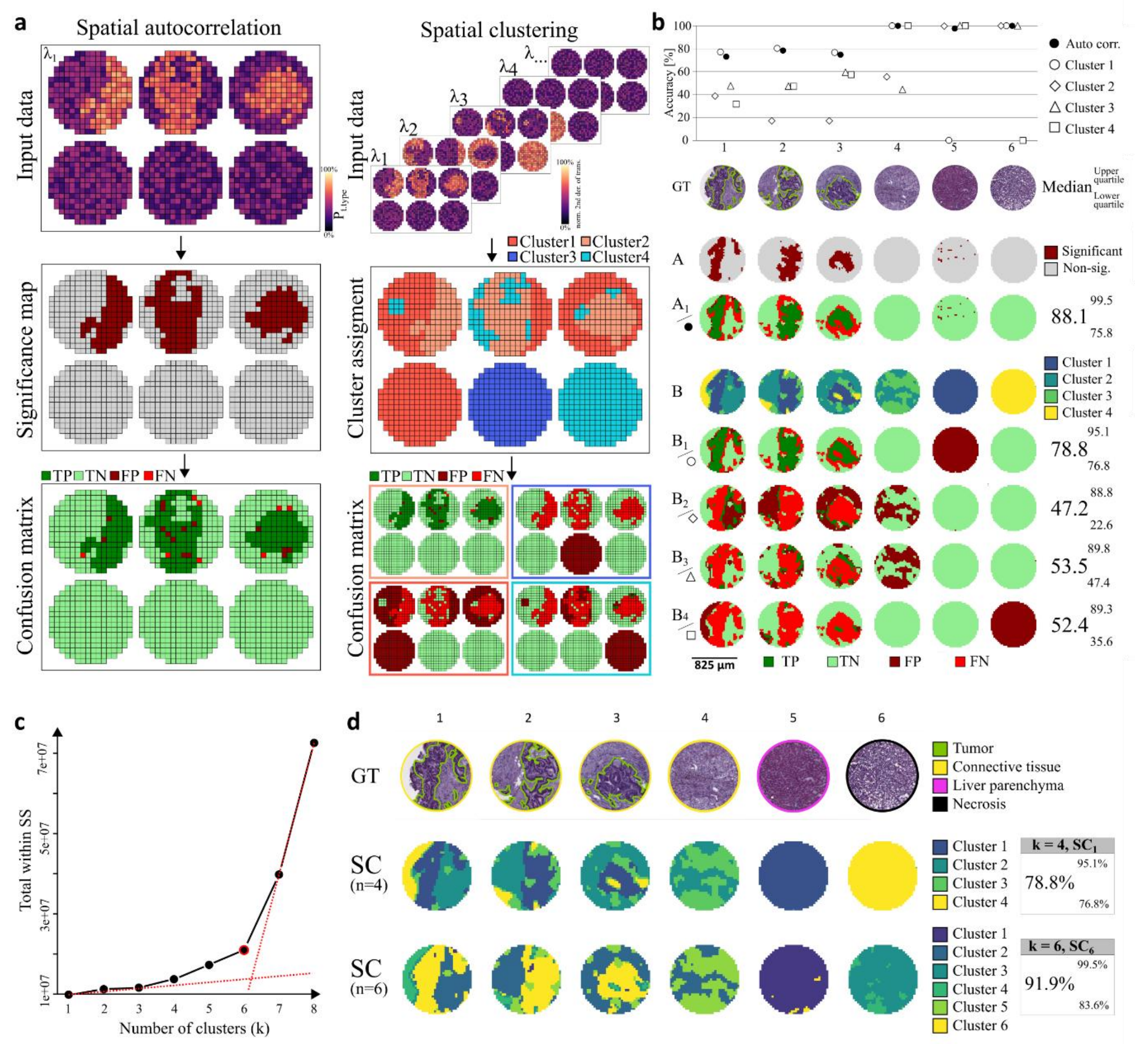

Figure S8. Spatial autocorrelation predicts tissue type annotations in less biased fashion than spatially aware clustering. **a)** Schematic representation of spatially aware clustering (SC, right) and spatial autocorrelation (SA, left), both implemented for cohort-wide assessment. While SA is usually implemented as a univariate technique (for either one feature or the projection of multiple features into one plane), no feature selection is required for SC. Whereas for SA the region of interest (ROI) assignment is accomplished by the method itself, in SC the number of clusters as well as the assignment of the cluster of interest (COI) needs to be known. Confusion matrix for pixel annotations as TP = true positive, TN = true negative, FP = false positive, FN = false negative, as compared to a pathologist-annotated ground truth (GT). **b)** Prior knowledge about the number of classes and the position of the COI in human samples (here, six tissue micro array (TMA) cores digitally punched from resected tissues of CRLM) is often limited. In comparison with histopathological annotation (GT provided in **(d)** top row), SA showed a median accuracy of 88.1% (SA1), while for SC (n = 4 clusters estimated by pathological annotation prior to computation) the median accuracy values ranged from 47.2% to 78.8% between the assigned clusters (B1 – B4). Even the correct cluster (B1) that mainly correlated with the tumor region (core1 – 3), falsely assigned core5 (liver parenchyma) to the tumor class. This may be a consequence of smaller variance between tumor and parenchyma than variances within the connective tissue (core4 split in two classes). This emphasizes the fact that for SC, the user-defined number of clusters as well as the nature of the COI investigated have a large impact on the results, whereas SA only requires the initial correct assignment of a suitable wavenumber feature, whereupon the results can be automatically assigned and are robust even for complex heterogeneous human samples. **c)** Computational estimation of the optimal number of classes used for clustering suggests 6 classes. **d)** Compared to pathologically estimated 4 classes, tissue type prediction with the computationally determined 6 classes performed better for the COI in this scenario. Nevertheless, manual assignment of the correct COI was required, which required the additional histopathological GT.

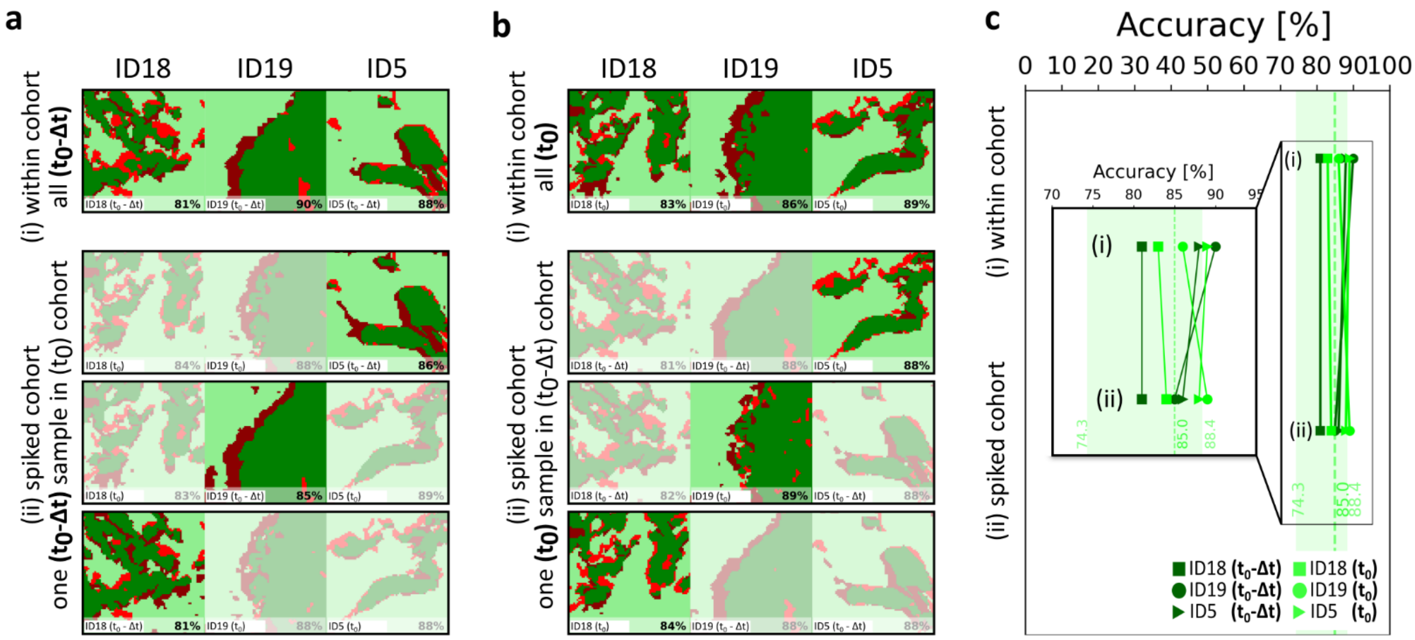

Figure S9. Spatial autocorrelation analysis performed on projection images compensated for variance between datasets measured at different time points. The same samples (IDs 18, 19, 5) were measured initially for method development (t_0 – Δt), as well as with the entire clinical cohort for the main study 2.5 years later (t_0). In comparison to (i) the calculation performed within each batch measured at the same time point (within 10 days), every possible combination of **a)** two samples of the (t_0) dataset spiked with one sample of the (t_0 – Δt) dataset, as well as **b)** two samples of the (t_0 – Δt) dataset spiked with one sample of the (t_0) dataset, calculated via cohort-wide SA analysis as a set of three, did not significantly change the accuracy of annotation. Additionally, no significant change in annotation accuracy was observed for the two samples from the same data batch, even when "spiked" with a sample measured at a different time point (transparent results). **(c)** Summary plotted for all samples between (i) within cohort and (ii) any possible combination of a "spiked cohort" (one sample outside of its own data batch), connected via a straight line to guide visual assessment. Accuracies were based on morphological annotations performed for tumor on an adjacent, H&E-stained section. Median and inter-quartile range transferred from accuracies reported in main study for entire cohort (n = 12) measured at the same time (dotted line and shaded rectangle in light green).

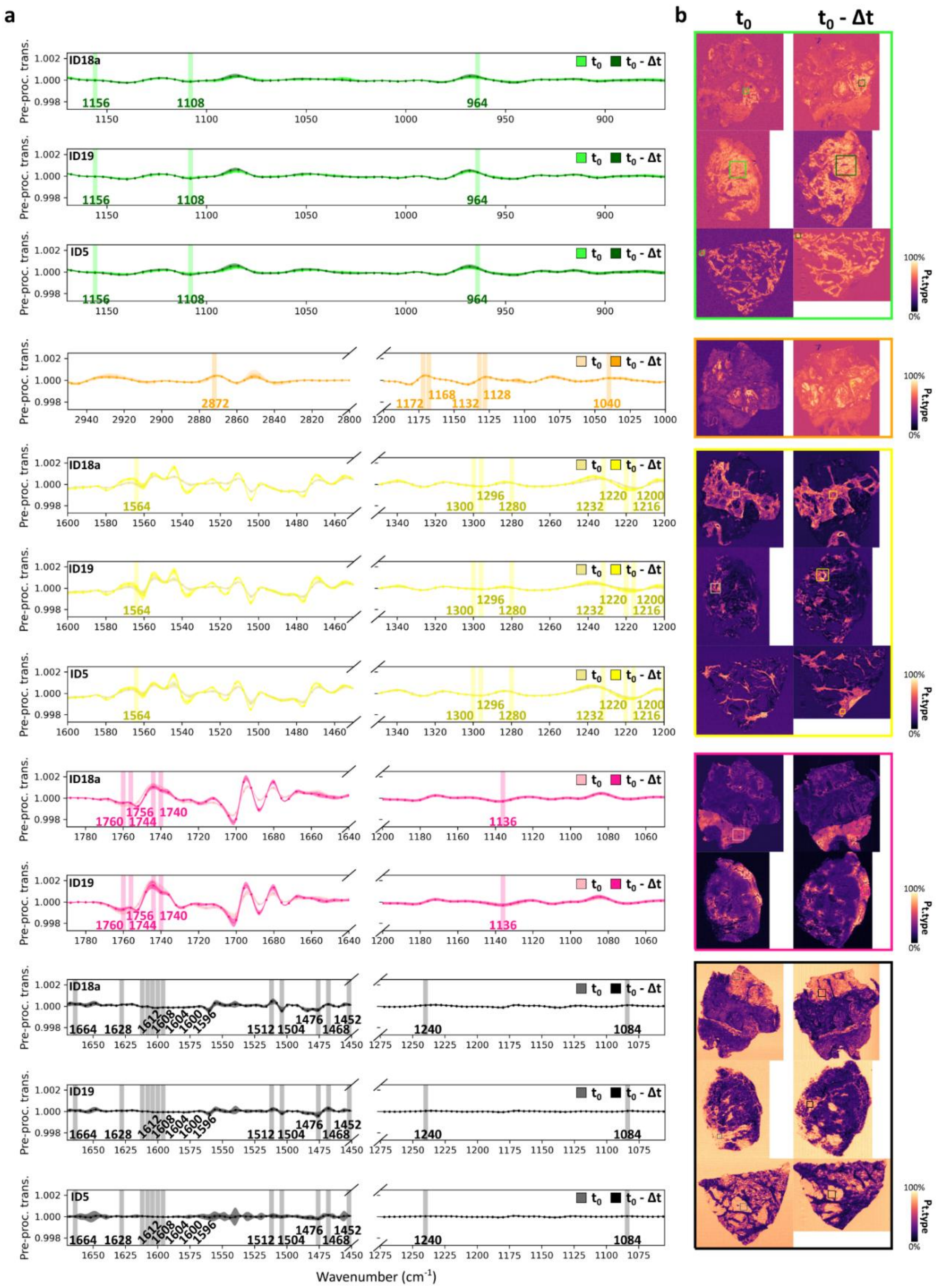

Figure S10. Pre-processed MIR imaging data measured at different time points. Three samples (IDs 18, 19, 5) were measured initially for method development ($t_0 - \Delta t$), as well as with the entire clinical cohort for the main study 2.5 years later (t_0). **a)** While slight changes in the pre-processed MIR spectra (mean per square as referenced in **(b)** with variance band spanning plus minus the standard deviation) can be observed, particularly wavenumbers selected via data-driven discriminant analysis (vertical lines) encountered barely visible variance. **b)** Projection images of all discriminant wavenumbers combined also showed no significant changes for most tissue types. Only the intensities on the slide background and necrotic regions of projection images for tumor and lumen showed a notable difference. Images were min-max normalized within each tissue type.

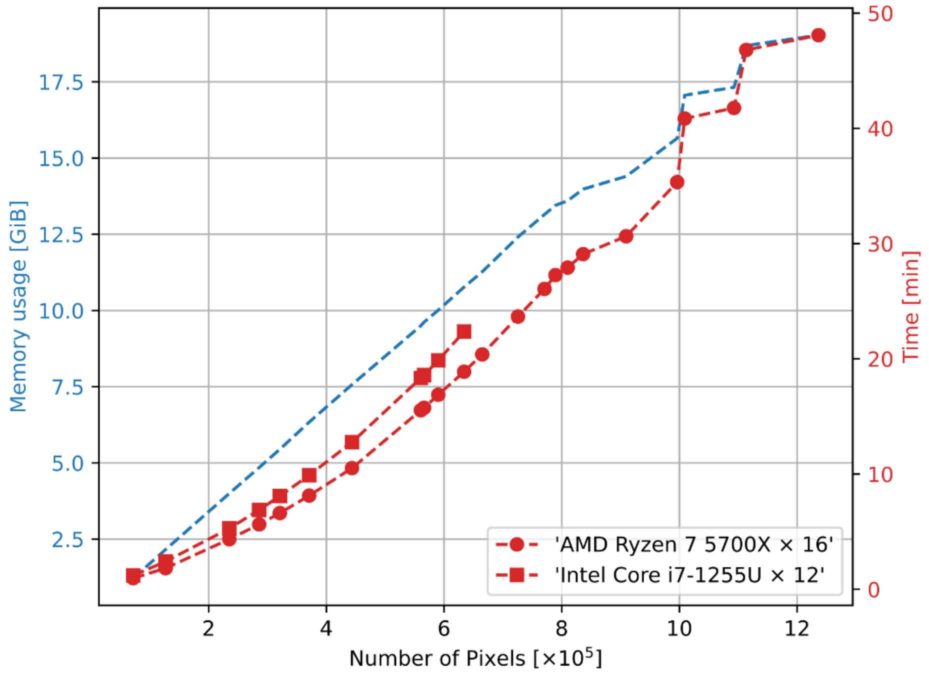

Figure S11. Cohort-wide processing of SA is limited by memory usage and computation time. Increasing sample numbers require a lot of memory (blue line, primary y-axis) and time (red lines, secondary y-axis) for calculation of cohort-wide processed SA analysis of clinical patient tissue cohorts, which limits the usability for normal desktop computers. When computed on a high-performance cluster (AMD, 3.4 GHz, 8 cores/ 16 threads, red circle), cohort-wide processing of SA analysis of tissue 23 samples required about 45 min computation time, while a normal desktop computer (Intel Core, CPU, 1.2 GHz, 10 cores/ 12 threads, red square) was able to compute only 11 of the samples due to limited computing power.

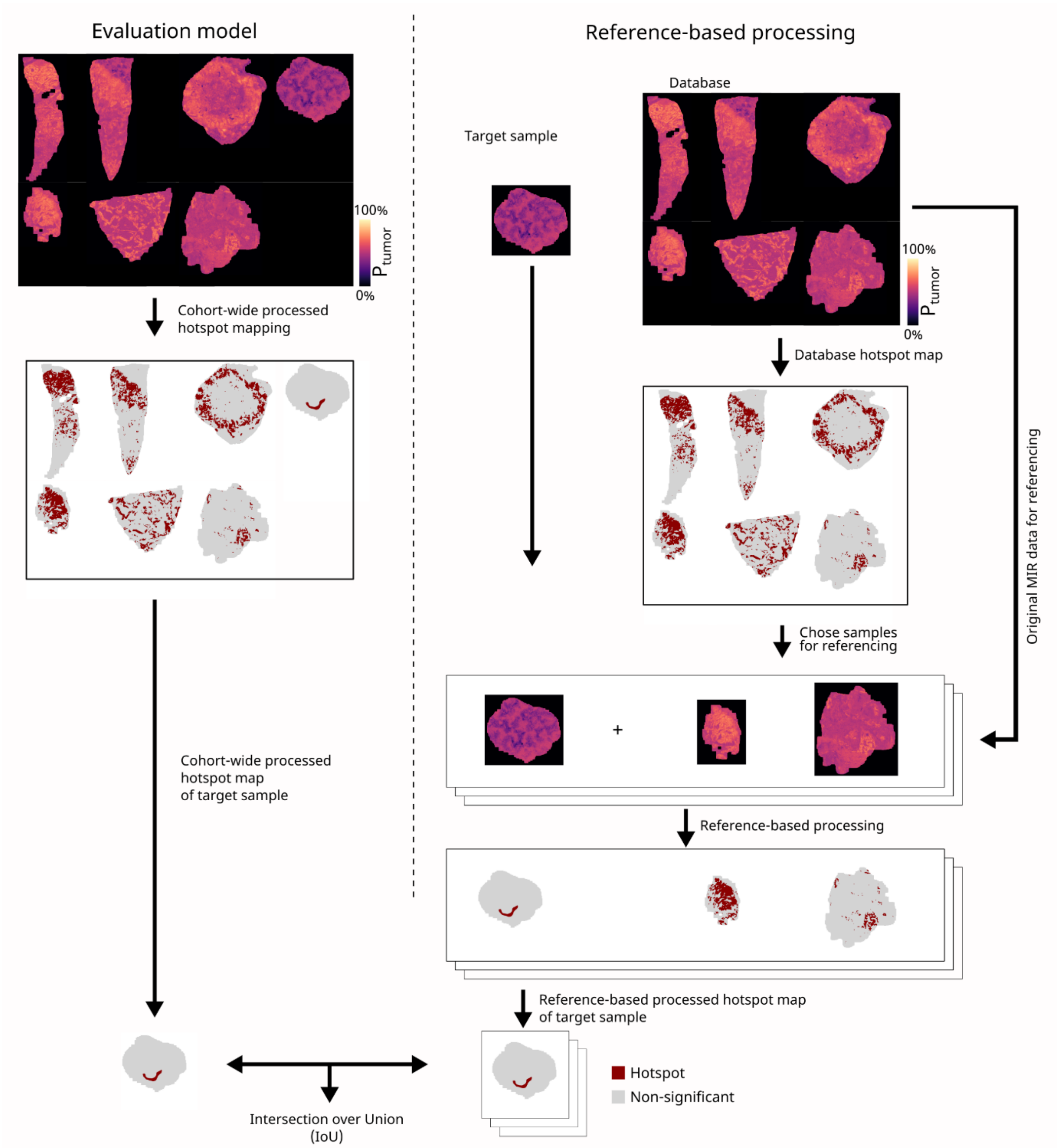

Figure S12. Setup of evaluation process for reference-based processing of SA. Left**:** Evaluation model provided via cohort-wide processing for unknown, newly added samples with database samples. Right: Reference-based processing performed on a new sample was permutated with different numbers of reference samples as well as any possible combination thereof. Results of both processes were compared via intersection over union (IoU) of the hotspot region.

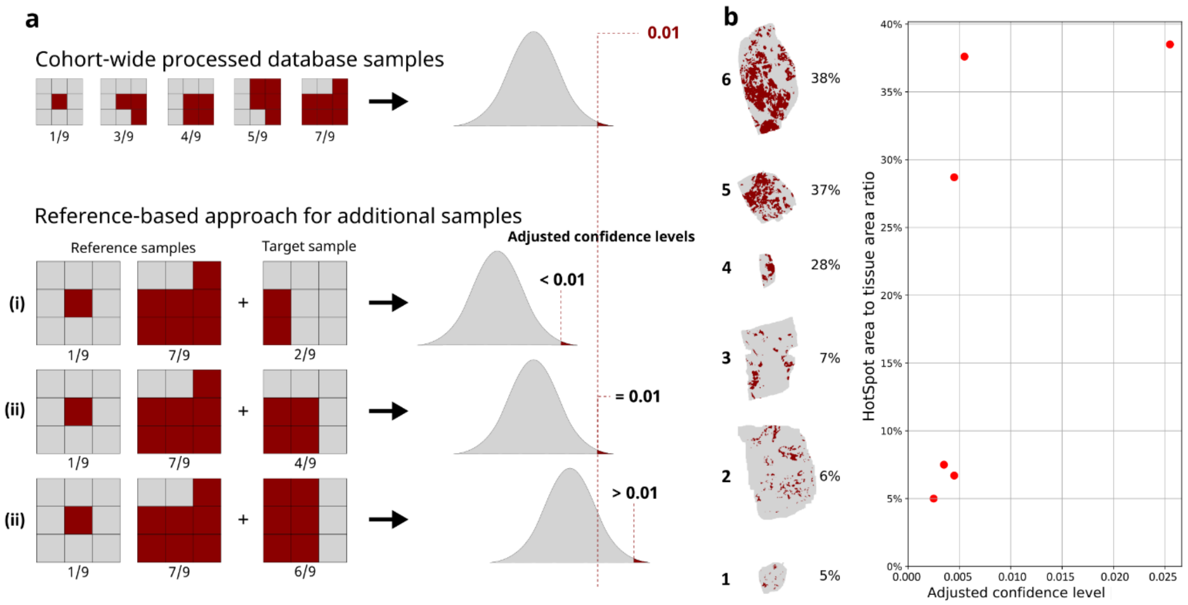

Figure S13. Adjusted confidence levels in reference-based processing versus initially set confidence level for calculation of the database. **a)** Schematic representation of rule of thumb connecting the initial confidence level used for database calculation (top 0.01, each tissue sample represented as 9 pixel grid with different numbers of hotspot pixels (red, 1 – 7 out of 9) with the adjusted confidence level used in the reference-based processing approach (bottom, i – iii). In cases where the newly added target sample has a lower (2/9) Moran's I-defined "hotspot-to-total tissue area" ratio calculated alongside the two reference samples from the database (lowest and highest hotspot-to-total tissue area ratio) compared to the cohort's average (4/9), the adjusted confidence level should tend towards lower values **(i)**, while remaining comparable if it is close (4/9) to the original cohort **(ii)**, and be adjusted to higher values if it represented a higher ratio (6/9) **(iii)**. However, this is a simplified estimation since SA analysis depends on the intensity distribution, as well as the size and the shape of the hotspot. **b)** This dependency between the "hotspot-to-total tissue area" ratio of the newly added target samples (y-axis) and the adjusted confidence levels (x-axis) was observed for reference-based processed SA of newly added samples of the CRLM cohort. However, since intensity values and hotspot shape were not considered in the estimation, outliers exist to this general rule of linear correlation (sample6). This emphasizes that confidence level adjustment cannot easily be estimated for the reference-based processing approach and needs to be determined in the iterative process alongside the reference samples judged against their original database composition.

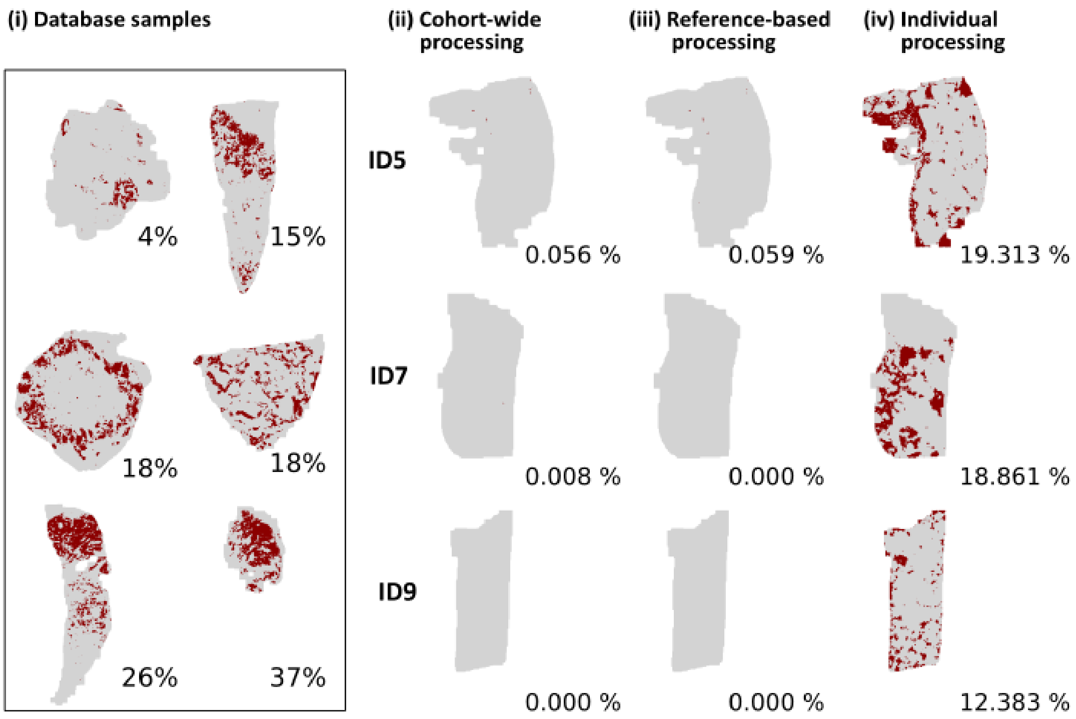

Figure S14. Performance of reference-based processing for negative control samples matches the cohort-wide processing and both outperform individual processing. Three negative control samples of different patients (IDs 5, 7, 9, containing only liver parenchyma) were annotated based on cohort-wide processing (**ii,** six tumor samples from the original database of the CRLM cohort (i) plus individually added control samples), reference-based processing (**iii,** calculated using (i) as database), and individual SA analysis (**iv,** each control sample computed individually). No false-positive tumor ROIs were assigned for negative controls in cohort-wide processing and reference-based processing, while individual SA calculation falsely assigned tumorous regions in all negative control samples.

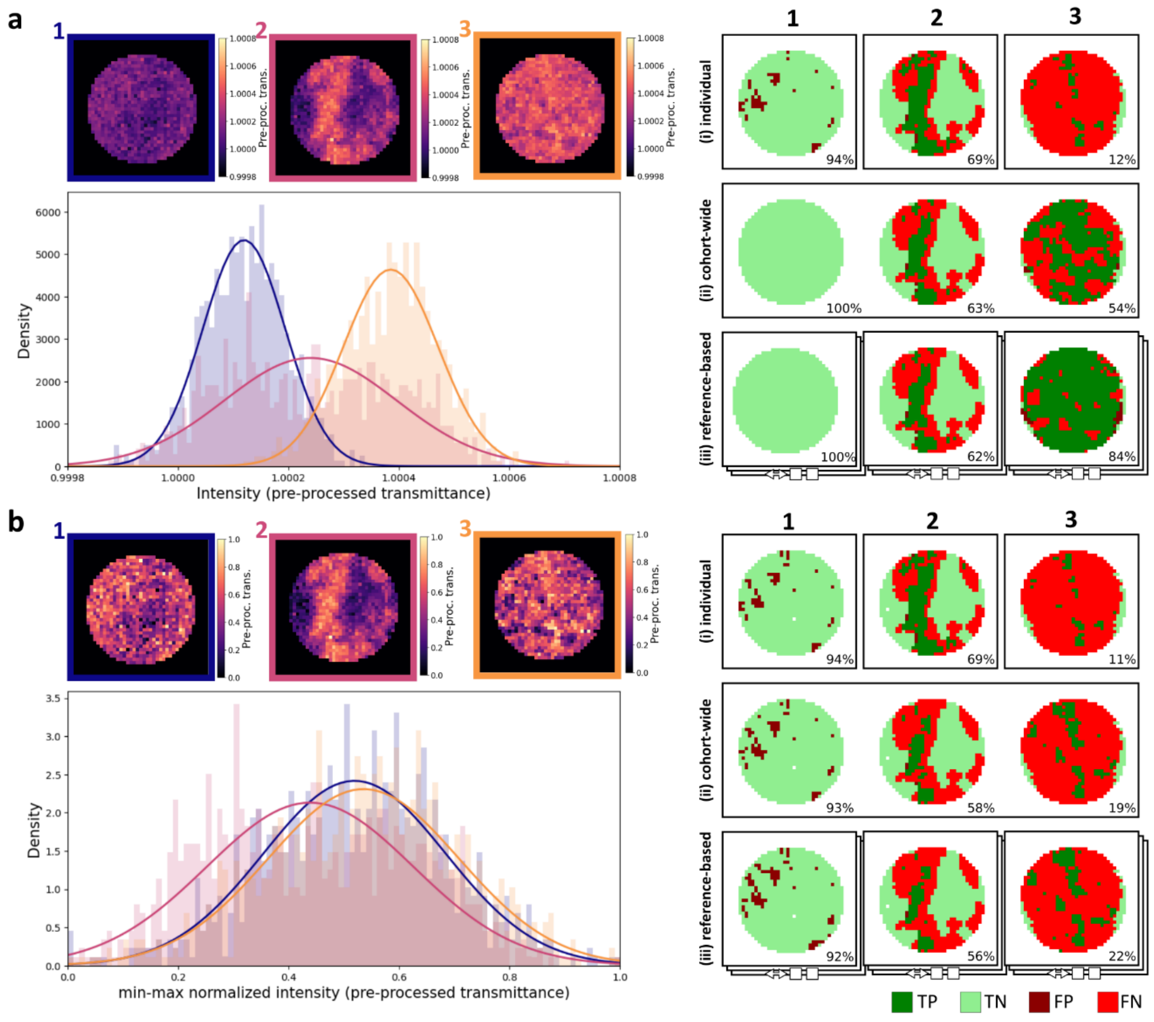

Figure S15. Only interdependent SA processing can relate multiple samples to each other and increase annotation accuracy. Hotspot mapping using local spatial autocorrelation (SA) analysis is based on the likelihood of high intensity values co-localizing with each other. Thus, normalization of intensity values cannot make samples more comparable for the analysis and increase annotation accuracy when processed individually. Therefore, interdependent processing, as implemented in cohort-wide and reference-based SA, is required to increase annotation accuracy especially of comparably homogenous samples. This was tested for three digitally punched cores (G18 diameter of 825 µm) obtained from MIR images (pre-processed transmittance at 964 cm^{-1}) of CRLM samples. **a)** Intensity data without additional normalization as compared to **b)** intensity data normalized to zero-to-one scale. While intensity distribution (left) can be equalized between multiple sample (here individual cores) when performing an additional normalization step, annotation accuracy (right) can only increase from (i) individual to (ii) cohort-wide or (iii) reference-based SA analysis, where the original intensity relation between samples is preserved. Thus, especially homogenous samples (core 1 no tumor and core 3 almost entirely tumorous) remain with low accuracy annotation where SA analysis is performed on equalized data (b). In comparison, with preserved intensity relation (a), cohort-wide processing increases the variance of each sample to the variance of the cohort, while the reference-based approach allows estimation (degree and direction) of the required confidence level adjustment for individual samples when based on two database reference samples. Thus, both increase annotation accuracy as compared to individual calculation. TP = true positive, TN = true negative, FP = false positive, FN = false negative.

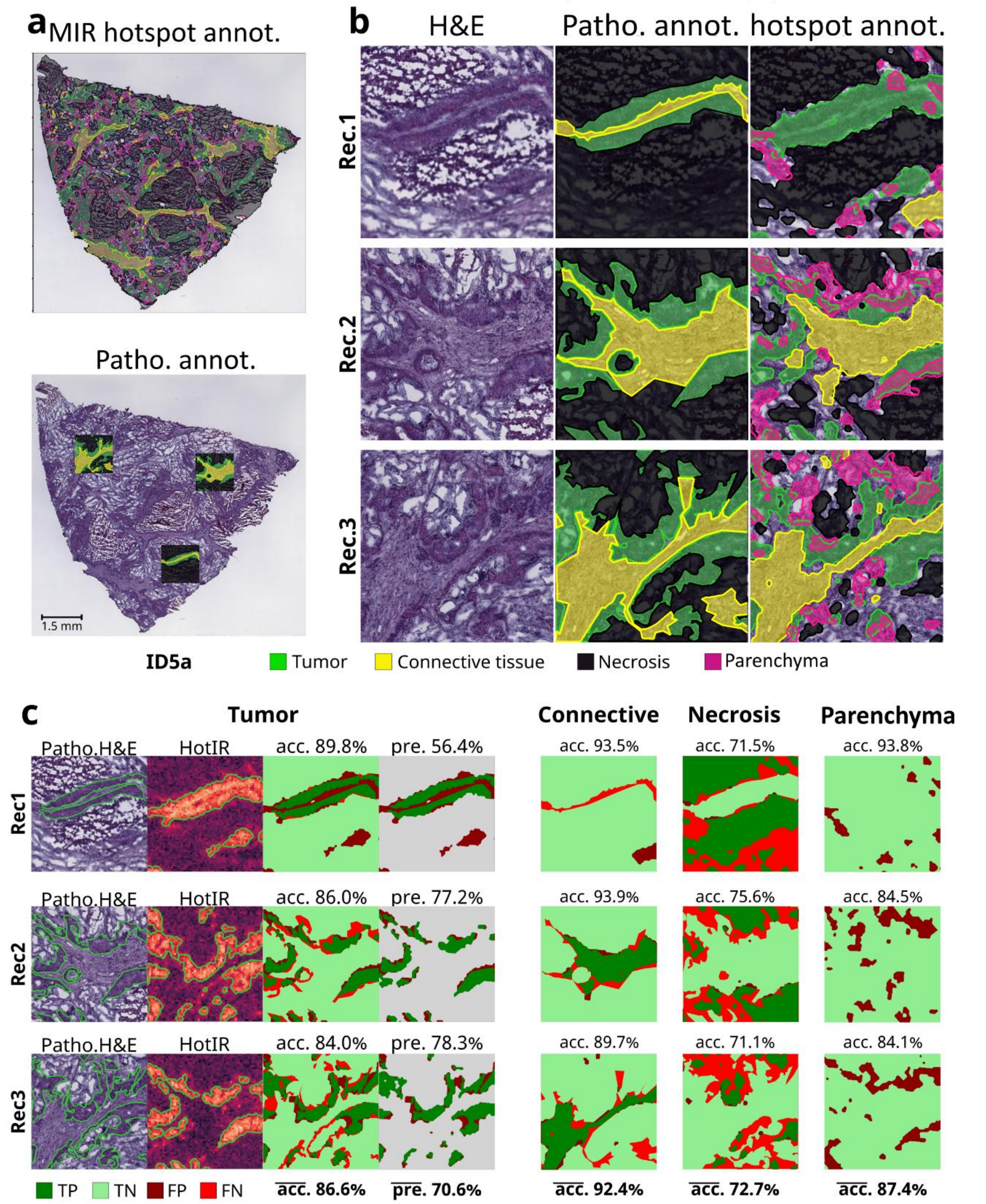

Figure S16. Comparison between mid-infrared imaging-based hotspot annotation and pathological annotations for patient ID5a. **a)** Adjacent sections were either automatically hotspot-annotated by mid-infrared (MIR) imaging and subsequent cohort-wide processed spatial autocorrelation analysis or H&E-stained and annotated by an expert pathologist (only three rectangles per patient). **b)** Zoom-ins to rectangles (rec.) evaluated by a pathologist including H&E-stained image without annotations (first column), pathological annotations (second column), and automated MIR hotspot annotations (third column). **c)** Analysis of accuracy of automated MIR hotspot annotations in comparison to manual histopathology for each rectangle and all tissue types. Here, accuracy (top, thin letters) represents how many pixels were correctly annotated (true positive and true negative) over all pixels of each rectangle. Patient mean accuracies (bottom, bold letters) were used for the summary statistics. Additionally, since tumor was included in all rectangles, the precision (number of pixels correctly annotated as tumor over all pixels annotated as tumor) of the hotspot annotation is provided.

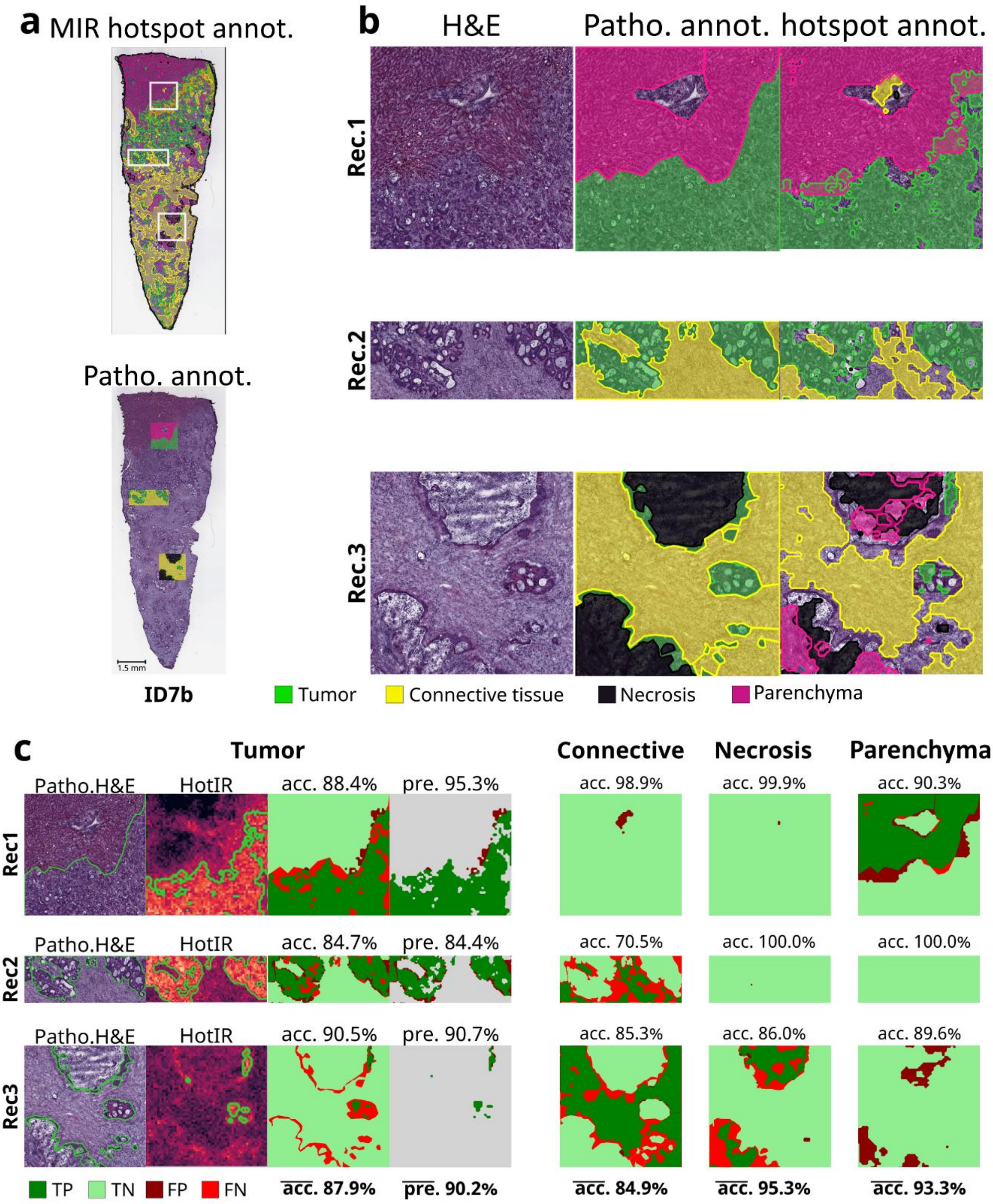

Figure S17. Comparison between mid-infrared imaging-based hotspot annotation and pathological annotations for patient ID7b. **a)** Adjacent sections were either automatically hotspot-annotated by mid-infrared (MIR) imaging and subsequent cohort-wide processed spatial autocorrelation analysis or H&E-stained and annotated by an expert pathologist (only three rectangles per patient). **b)** Zoom-ins to rectangles (rec.) evaluated by a pathologist including H&E-stained image without annotations (first column), pathological annotations (second column), and automated MIR hotspot annotations (third column). **c)** Analysis of accuracy of automated MIR hotspot annotations in comparison to manual histopathology for each rectangle and all tissue types. Here, accuracy (top, thin letters) represents how many pixels were correctly annotated (true positive and true negative) over all pixels of each rectangle. Patient mean accuracies (bottom, bold letters) were used for the summary statistics. Additionally, since tumor was included in all rectangles, the precision (number of pixels correctly annotated as tumor over all pixels annotated as tumor) of the hotspot annotation is provided.

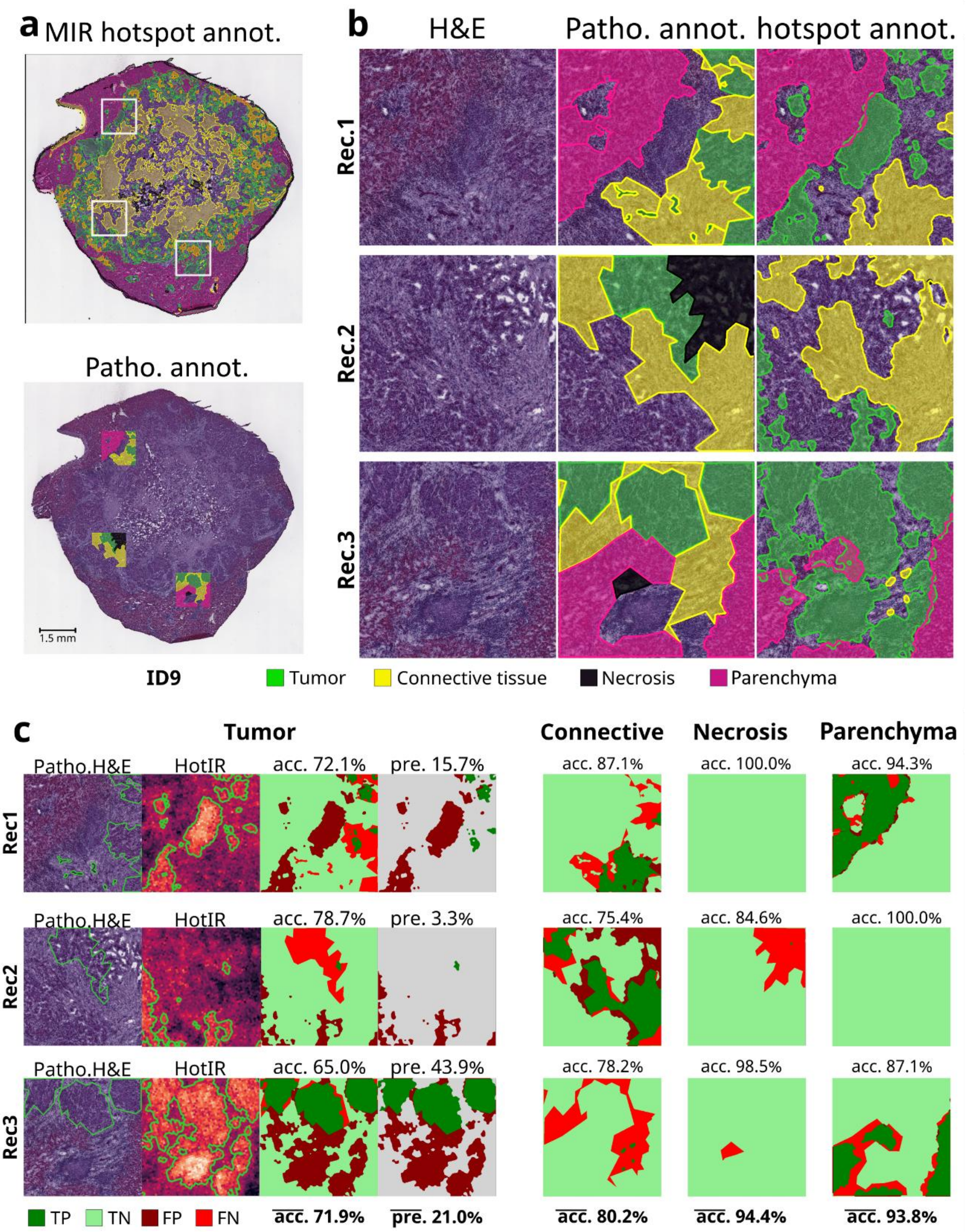

Figure S18. Comparison between mid-infrared imaging-based hotspot annotation and pathological annotations for patient ID9. **a)** Adjacent sections were either automatically hotspot-annotated by mid-infrared (MIR) imaging and subsequent cohort-wide processed spatial autocorrelation analysis or H&E-stained and annotated by an expert pathologist (only three rectangles per patient). **b)** Zoom-ins to rectangles (rec.) evaluated by a pathologist including H&E-stained image without annotations (first column), pathological annotations (second column), and automated MIR hotspot annotations (third column). **c)** Analysis of accuracy of automated MIR hotspot annotations in comparison to manual histopathology for each rectangle and all tissue types. Here, accuracy (top, thin letters) represents how many pixels were correctly annotated (true positive and true negative) over all pixels of each rectangle. Patient mean accuracies (bottom, bold letters) were used for the summary statistics. Additionally, since tumor was included in all rectangles, the precision (number of pixels correctly annotated as tumor over all pixels annotated as tumor) of the hotspot annotation is provided.

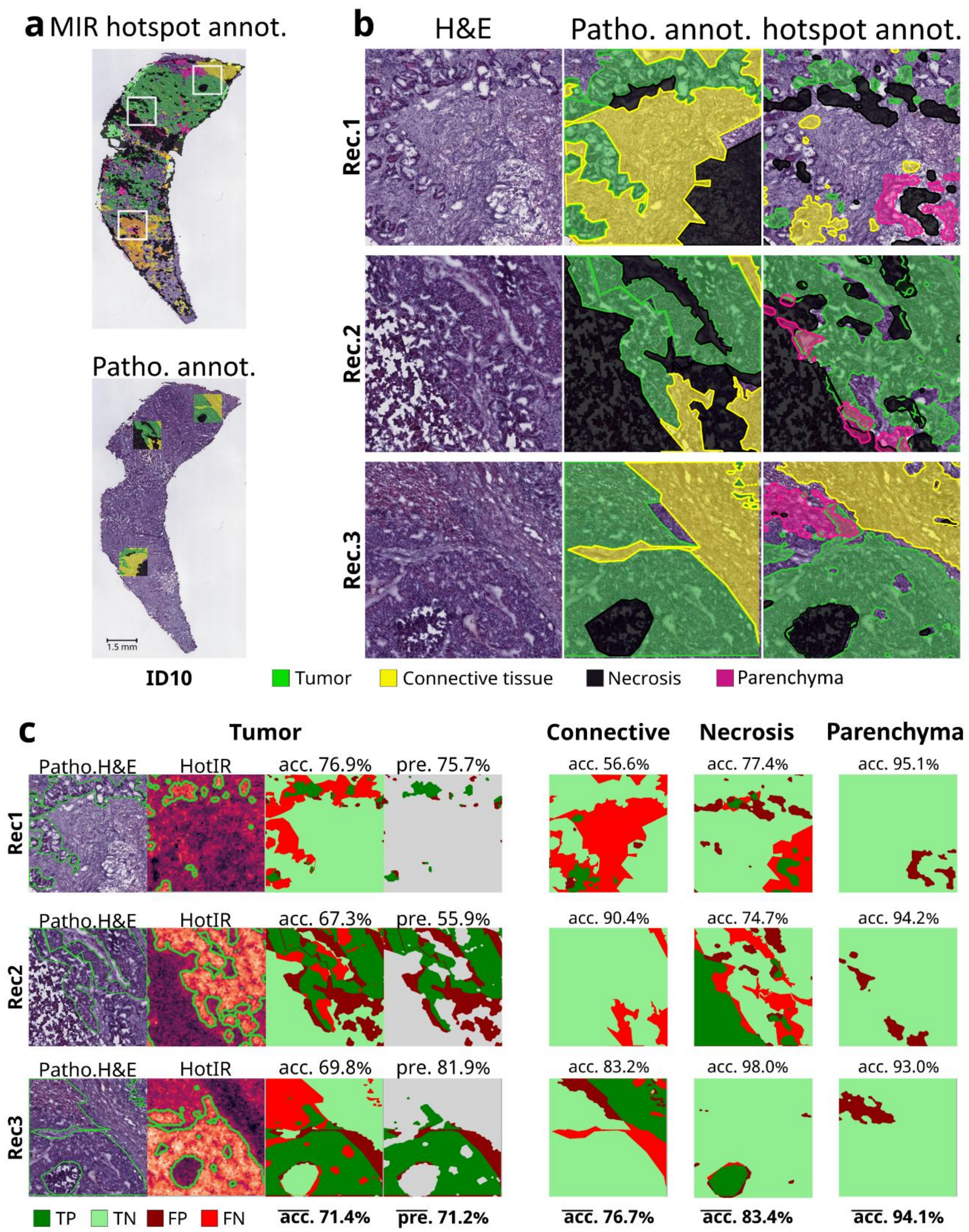

Figure S19. Comparison between mid-infrared imaging-based hotspot annotation and pathological annotations for patient ID10. **a)** Adjacent sections were either automatically hotspot-annotated by mid-infrared (MIR) imaging and subsequent cohort-wide processed spatial autocorrelation analysis or H&E-stained and annotated by an expert pathologist (only three rectangles per patient). **b)** Zoom-ins to rectangles (rec.) evaluated by a pathologist including H&E-stained image without annotations (first column), pathological annotations (second column), and automated MIR hotspot annotations (third column). **c)** Analysis of accuracy of automated MIR hotspot annotations in comparison to manual histopathology for each rectangle and all tissue types. Here, accuracy (top, thin letters) represents how many pixels were correctly annotated (true positive and true negative) over all pixels of each rectangle. Patient mean accuracies (bottom, bold letters) were used for the summary statistics. Additionally, since tumor was included in all rectangles, the precision (number of pixels correctly annotated as tumor over all pixels annotated as tumor) of the hotspot annotation is provided.

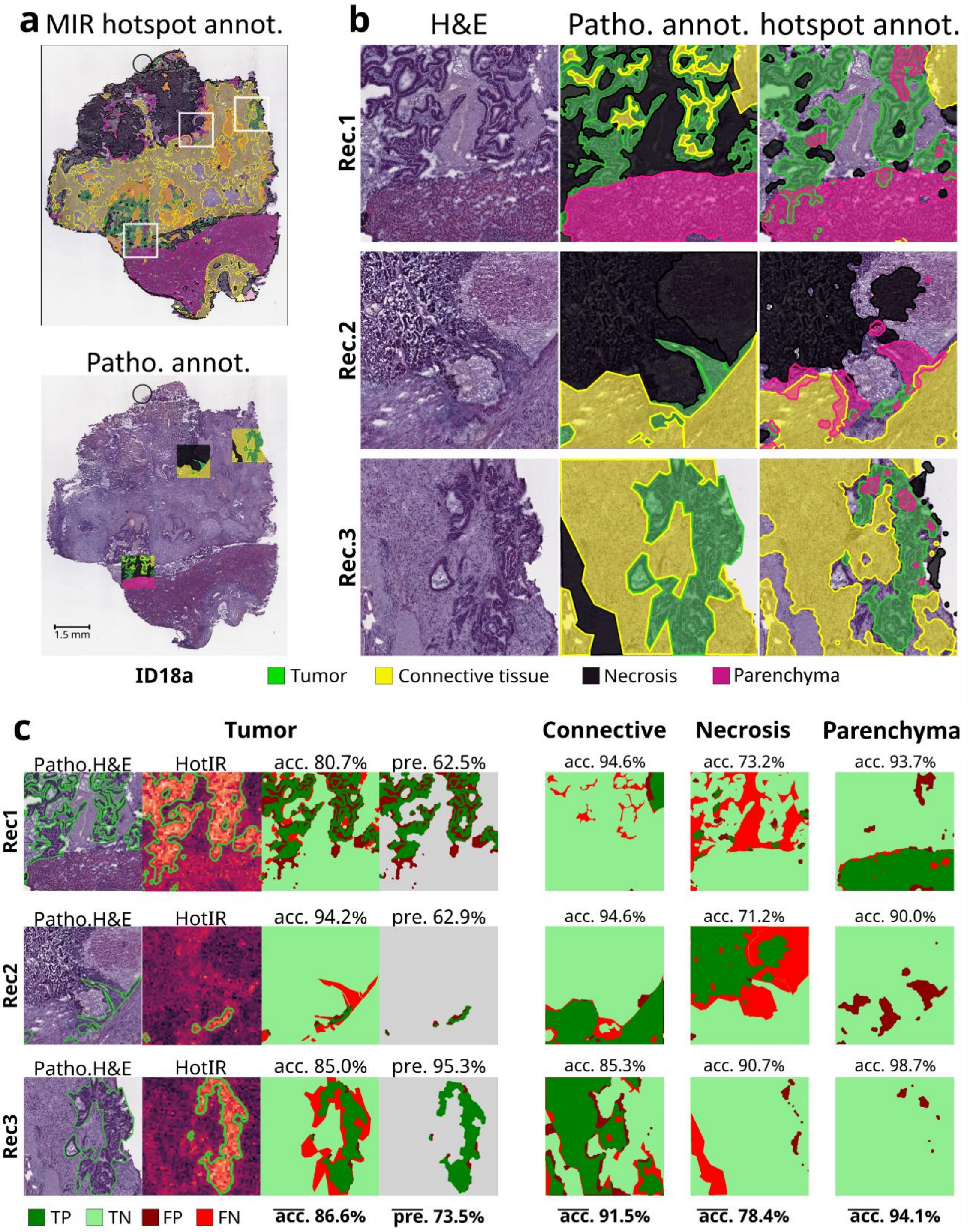

Figure S20. Comparison between mid-infrared imaging-based hotspot annotation and pathological annotations for patient ID18a. **a)** Adjacent sections were either automatically hotspot-annotated by mid-infrared (MIR) imaging and subsequent cohort-wide processed spatial autocorrelation analysis or H&E-stained and annotated by an expert pathologist (only three rectangles per patient). **b)** Zoom-ins to rectangles (rec.) evaluated by a pathologist including H&E-stained image without annotations (first column), pathological annotations (second column), and automated MIR hotspot annotations (third column). **c)** Analysis of accuracy of automated MIR hotspot annotations in comparison to manual histopathology for each rectangle and all tissue types. Here, accuracy (top, thin letters) represents how many pixels were correctly annotated (true positive and true negative) over all pixels of each rectangle. Patient mean accuracies (bottom, bold letters) were used for the summary statistics. Additionally, since tumor was included in all rectangles, the precision (number of pixels correctly annotated as tumor over all pixels annotated as tumor) of the hotspot annotation is provided.

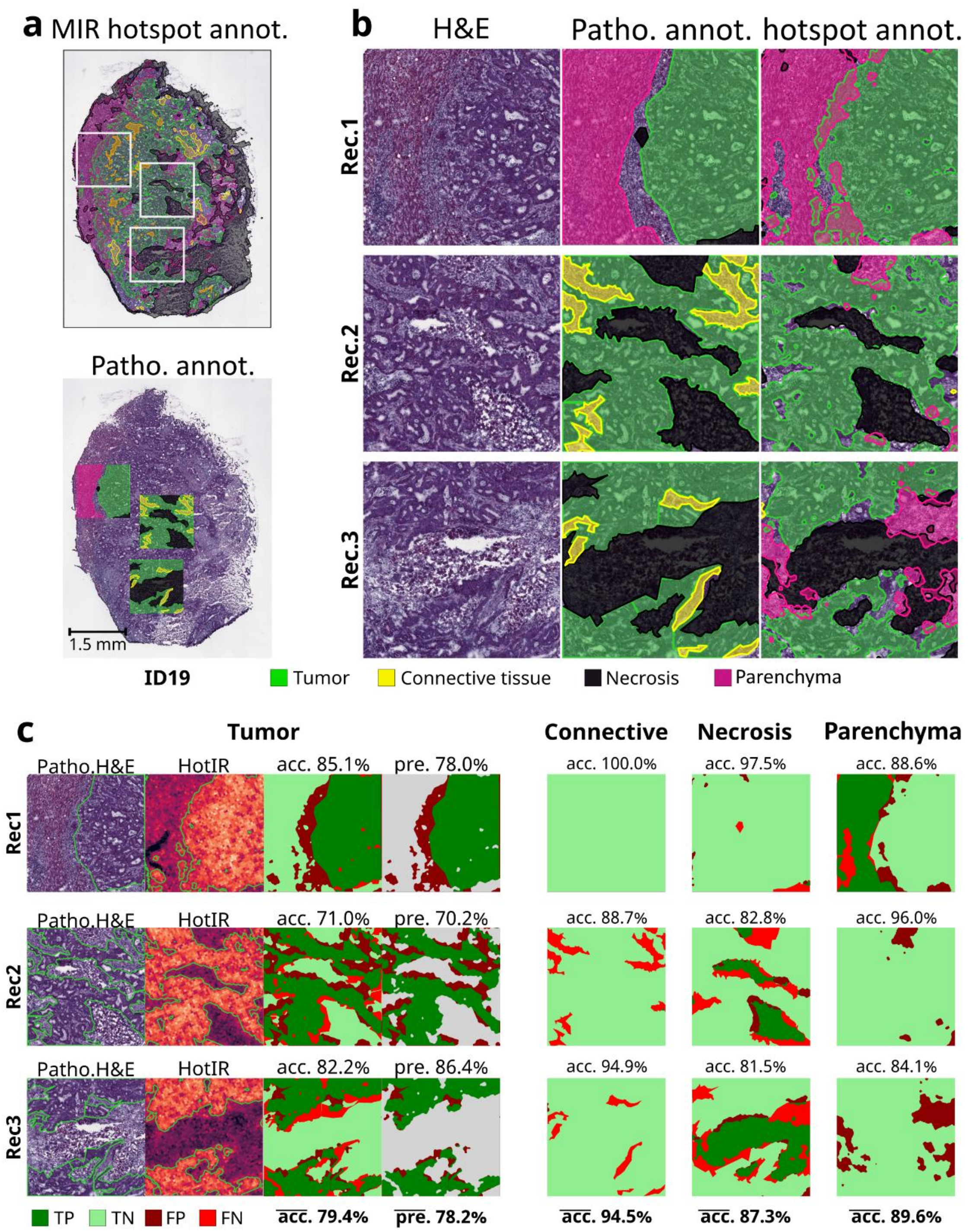

Figure S21. Comparison between mid-infrared imaging-based hotspot annotation and pathological annotations for patient ID19. **a)** Adjacent sections were either automatically hotspot-annotated by mid-infrared (MIR) imaging and subsequent cohort-wide processed spatial autocorrelation analysis or H&E-stained and annotated by an expert pathologist (only three rectangles per patient). **b)** Zoom-ins to rectangles (rec.) evaluated by a pathologist including H&E-stained image without annotations (first column), pathological annotations (second column), and automated MIR hotspot annotations (third column). **c)** Analysis of accuracy of automated MIR hotspot annotations in comparison to manual histopathology for each rectangle and all tissue types. Here, accuracy (top, thin letters) represents how many pixels were correctly annotated (true positive and true negative) over all pixels of each rectangle. Patient mean accuracies (bottom, bold letters) were used for the summary statistics. Additionally, since tumor was included in all rectangles, the precision (number of pixels correctly annotated as tumor over all pixels annotated as tumor) of the hotspot annotation is provided.

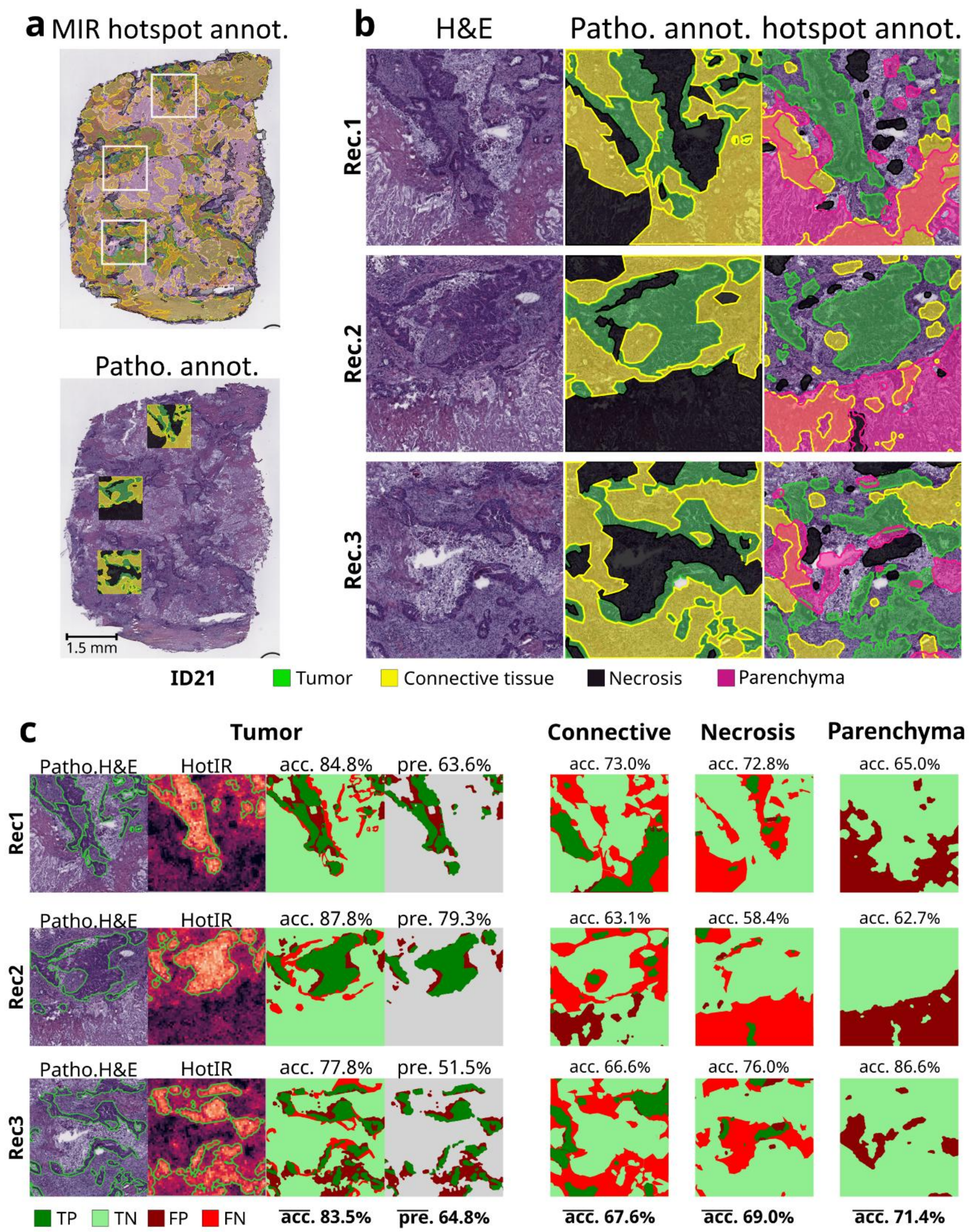

Figure S22. Comparison between mid-infrared imaging-based hotspot annotation and pathological annotations for patient ID21. **a)** Adjacent sections were either automatically hotspot-annotated by mid-infrared (MIR) imaging and subsequent reference-based processed spatial autocorrelation analysis or H&E-stained and annotated by an expert pathologist (only three rectangles per patient). **b)** Zoom-ins to rectangles (rec.) evaluated by a pathologist including H&E-stained image without annotations (first column), pathological annotations (second column), and automated MIR hotspot annotations (third column). **c)** Analysis of accuracy of automated MIR hotspot annotations in comparison to manual histopathology for each rectangle and all tissue types. Here, accuracy (top, thin letters) represents how many pixels were correctly annotated (true positive and true negative) over all pixels of each rectangle. Patient mean accuracies (bottom, bold letters) were used for the summary statistics. Additionally, since tumor was included in all rectangles, the precision (number of pixels correctly annotated as tumor over all pixels annotated as tumor) of the hotspot annotation is provided.

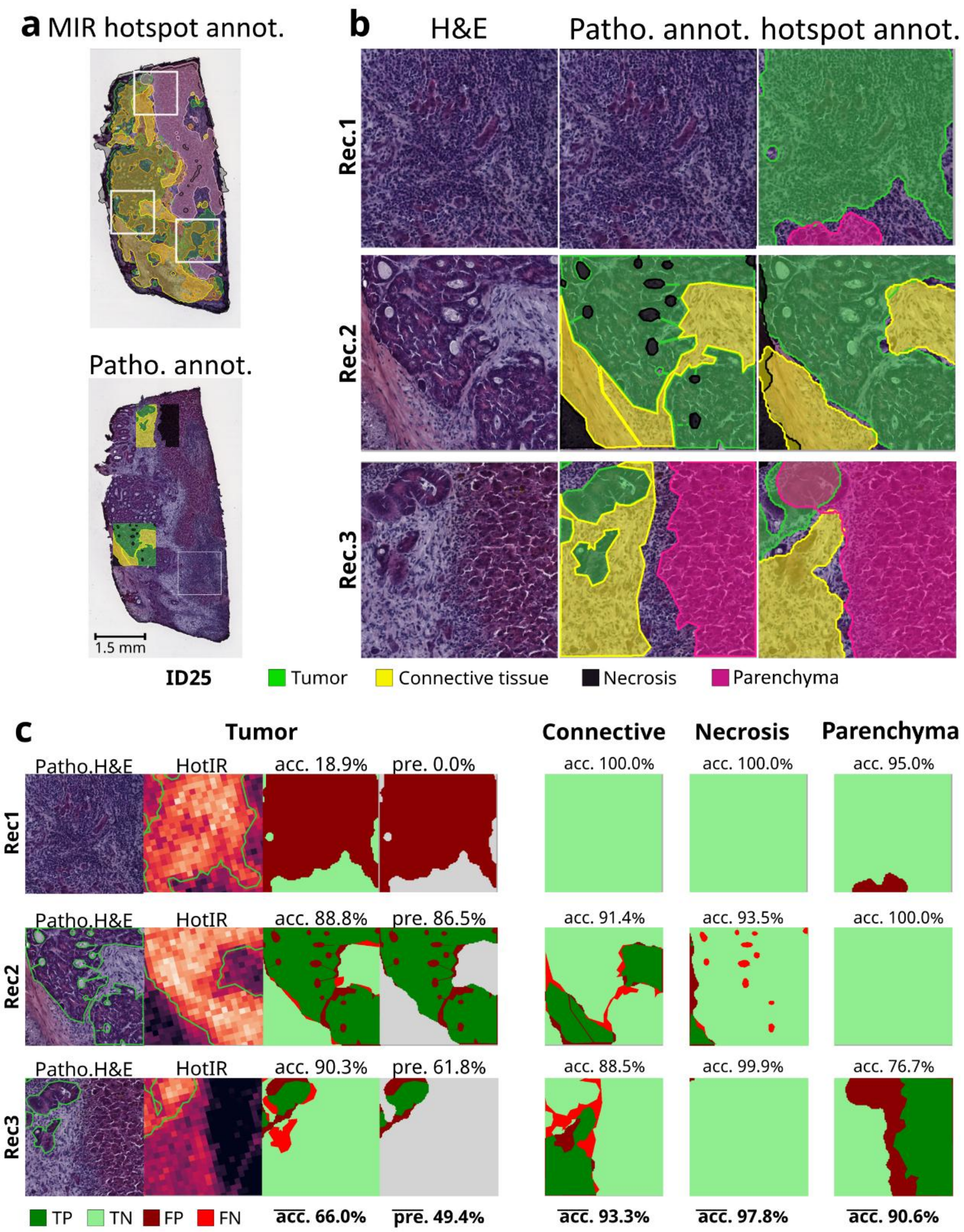

Figure S23. Comparison between mid-infrared imaging-based hotspot annotation and pathological annotations for patient ID25. **a)** Adjacent sections were either automatically hotspot-annotated by mid-infrared (MIR) imaging and subsequent reference-based processed spatial autocorrelation analysis or H&E-stained and annotated by an expert pathologist (only three rectangles per patient). **b)** Zoom-ins to rectangles (rec.) evaluated by a pathologist including H&E-stained image without annotations (first column), pathological annotations (second column), and automated MIR hotspot annotations (third column). **c)** Analysis of accuracy of automated MIR hotspot annotations in comparison to manual histopathology for each rectangle and all tissue types. Here, accuracy (top, thin letters) represents how many pixels were correctly annotated (true positive and true negative) over all pixels of each rectangle. Patient mean accuracies (bottom, bold letters) were used for the summary statistics. Additionally, since tumor was included in all rectangles, the precision (number of pixels correctly annotated as tumor over all pixels annotated as tumor) of the hotspot annotation is provided.

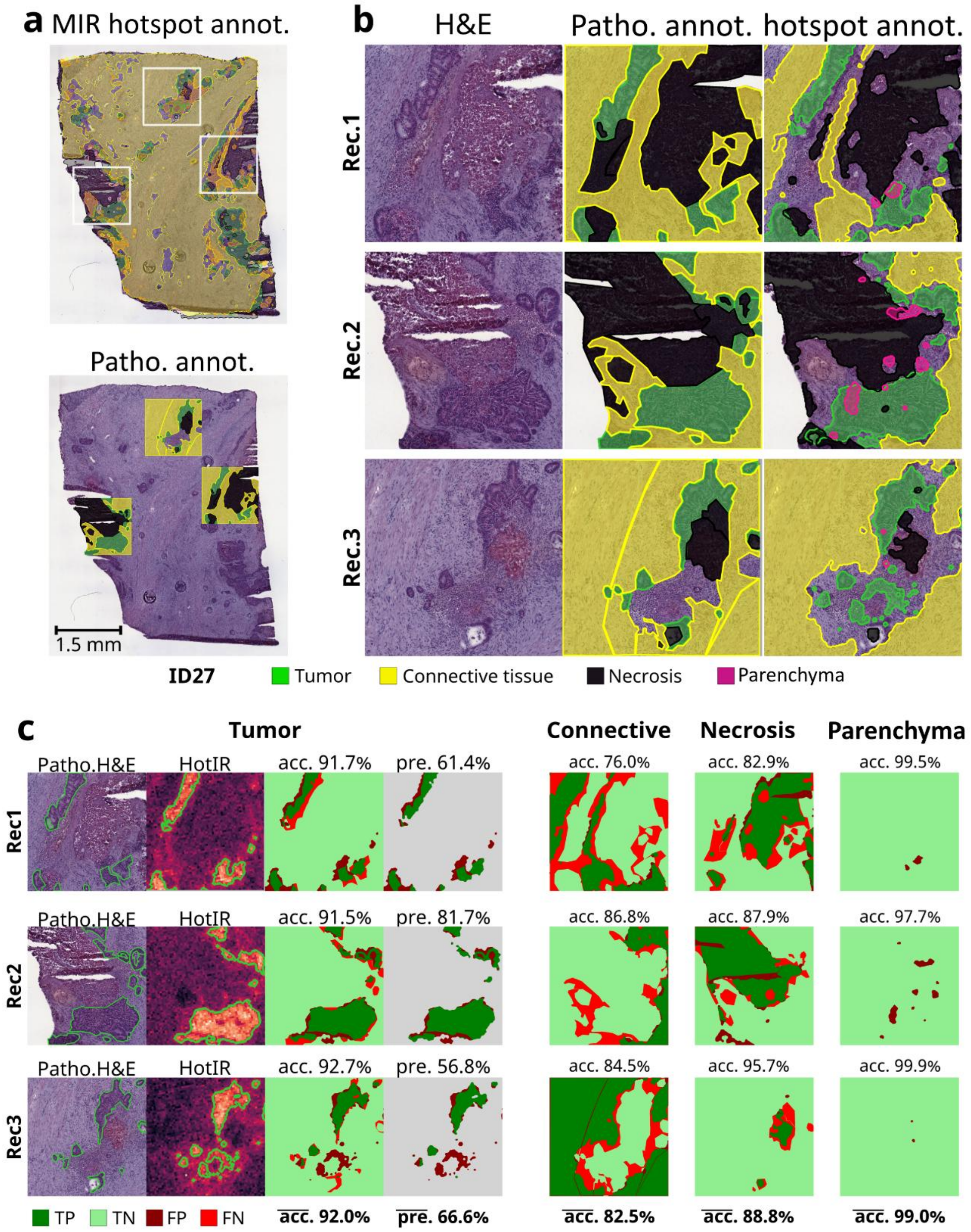

Figure S24. Comparison between mid-infrared imaging-based hotspot annotation and pathological annotations for patient ID27. **a)** Adjacent sections were either automatically hotspot-annotated by mid-infrared (MIR) imaging and subsequent reference-based processed spatial autocorrelation analysis or H&E-stained and annotated by an expert pathologist (only three rectangles per patient). **b)** Zoom-ins to rectangles (rec.) evaluated by a pathologist including H&E-stained image without annotations (first column), pathological annotations (second column), and automated MIR hotspot annotations (third column). **c)** Analysis of accuracy of automated MIR hotspot annotations in comparison to manual histopathology for each rectangle and all tissue types. Here, accuracy (top, thin letters) represents how many pixels were correctly annotated (true positive and true negative) over all pixels of each rectangle. Patient mean accuracies (bottom, bold letters) were used for the summary statistics. Additionally, since tumor was included in all rectangles, the precision (number of pixels correctly annotated as tumor over all pixels annotated as tumor) of the hotspot annotation is provided.

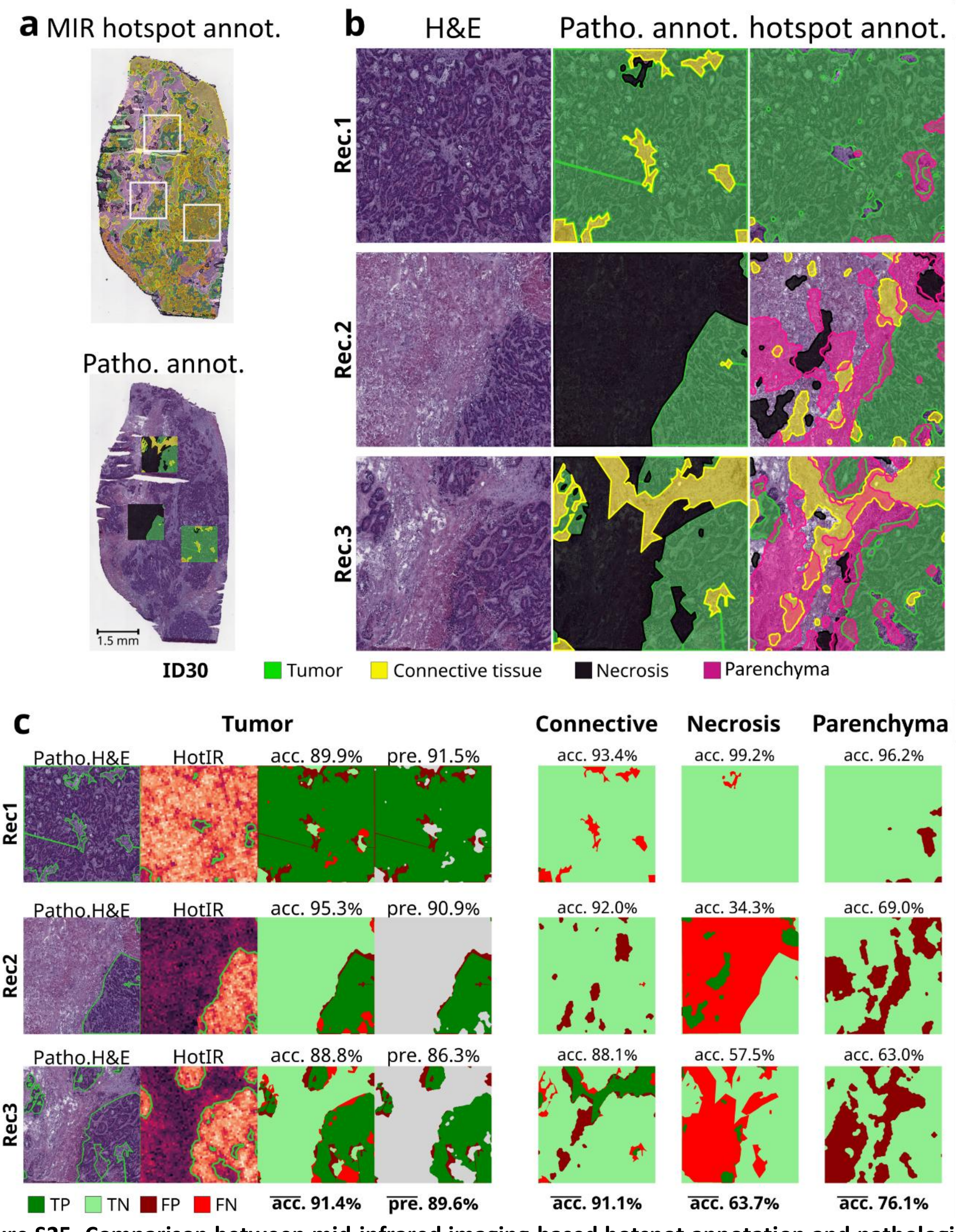

Figure S25. Comparison between mid-infrared imaging-based hotspot annotation and pathological annotations for patient ID30. **a)** Adjacent sections were either automatically hotspot-annotated by mid-infrared (MIR) imaging and subsequent reference-based processed spatial autocorrelation analysis or H&E-stained and annotated by an expert pathologist (only three rectangles per patient). **b)** Zoom-ins to rectangles (rec.) evaluated by a pathologist including H&E-stained image without annotations (first column), pathological annotations (second column), and automated MIR hotspot annotations (third column). **c)** Analysis of accuracy of automated MIR hotspot annotations in comparison to manual histopathology for each rectangle and all tissue types. Here, accuracy (top, thin letters) represents how many pixels were correctly annotated (true positive and true negative) over all pixels of each rectangle. Patient mean accuracies (bottom, bold letters) were used for the summary statistics. Additionally, since tumor was included in all rectangles, the precision (number of pixels correctly annotated as tumor over all pixels annotated as tumor) of the hotspot annotation is provided.

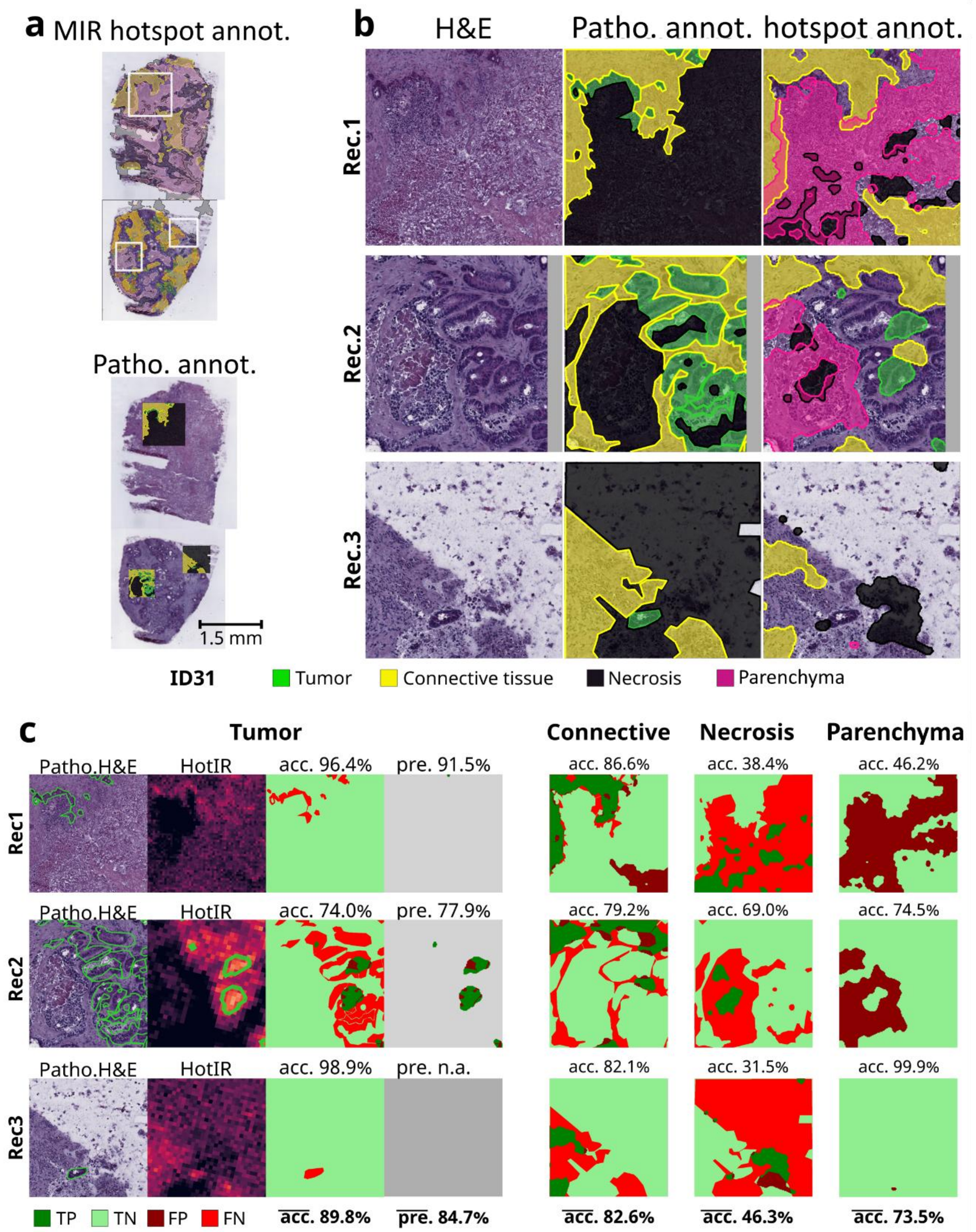

Figure S26. Comparison between mid-infrared imaging-based hotspot annotation and pathological annotations for patient ID31. **a)** Adjacent sections were either automatically hotspot-annotated by mid-infrared (MIR) imaging and subsequent reference-based processed spatial autocorrelation analysis or H&E-stained and annotated by an expert pathologist (only three rectangles per patient). **b)** Zoom-ins to rectangles (rec.) evaluated by a pathologist including H&E-stained image without annotations (first column), pathological annotations (second column), and automated MIR hotspot annotations (third column). **c)** Analysis of accuracy of automated MIR hotspot annotations in comparison to manual histopathology for each rectangle and all tissue types. Here, accuracy (top, thin letters) represents how many pixels were correctly annotated (true positive and true negative) over all pixels of each rectangle. Patient mean accuracies (bottom, bold letters) were used for the summary statistics. Additionally, since tumor was included in all rectangles, the precision (number of pixels correctly annotated as tumor over all pixels annotated as tumor) of the hotspot annotation is provided.

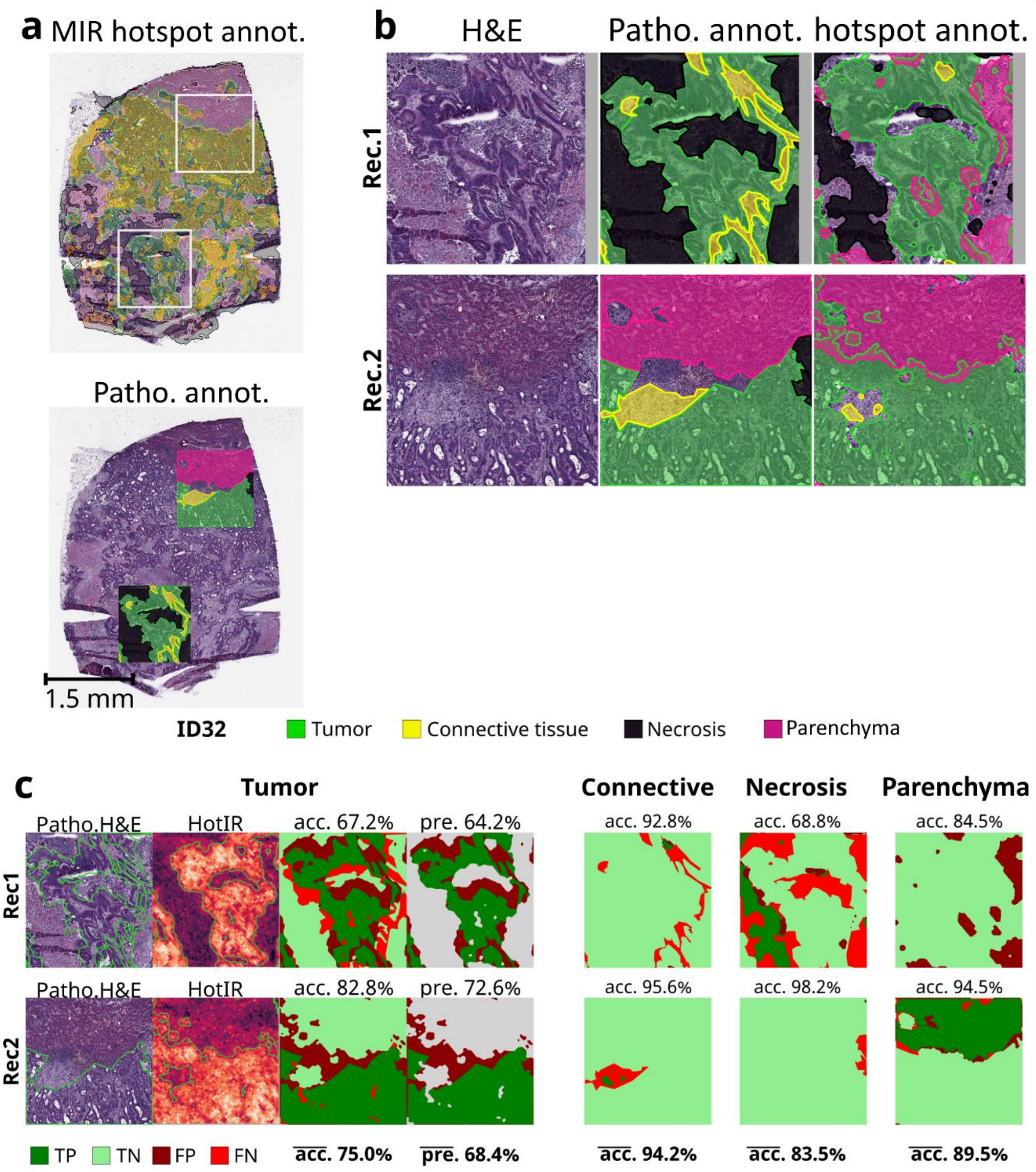

Figure S27. Comparison between mid-infrared imaging-based hotspot annotation and pathological annotations for patient ID32. **a)** Adjacent sections were either automatically hotspot-annotated by mid-infrared (MIR) imaging and subsequent reference-based processed spatial autocorrelation analysis or H&E-stained and annotated by an expert pathologist (only three rectangles per patient). **b)** Zoom-ins to rectangles (rec.) evaluated by a pathologist including H&E-stained image without annotations (first column), pathological annotations (second column), and automated MIR hotspot annotations (third column). **c)** Analysis of accuracy of automated MIR hotspot annotations in comparison to manual histopathology for each rectangle and all tissue types. Here, accuracy (top, thin letters) represents how many pixels were correctly annotated (true positive and true negative) over all pixels of each rectangle. Patient mean accuracies (bottom, bold letters) were used for the summary statistics. Additionally, since tumor was included in all rectangles, the precision (number of pixels correctly annotated as tumor over all pixels annotated as tumor) of the hotspot annotation is provided.

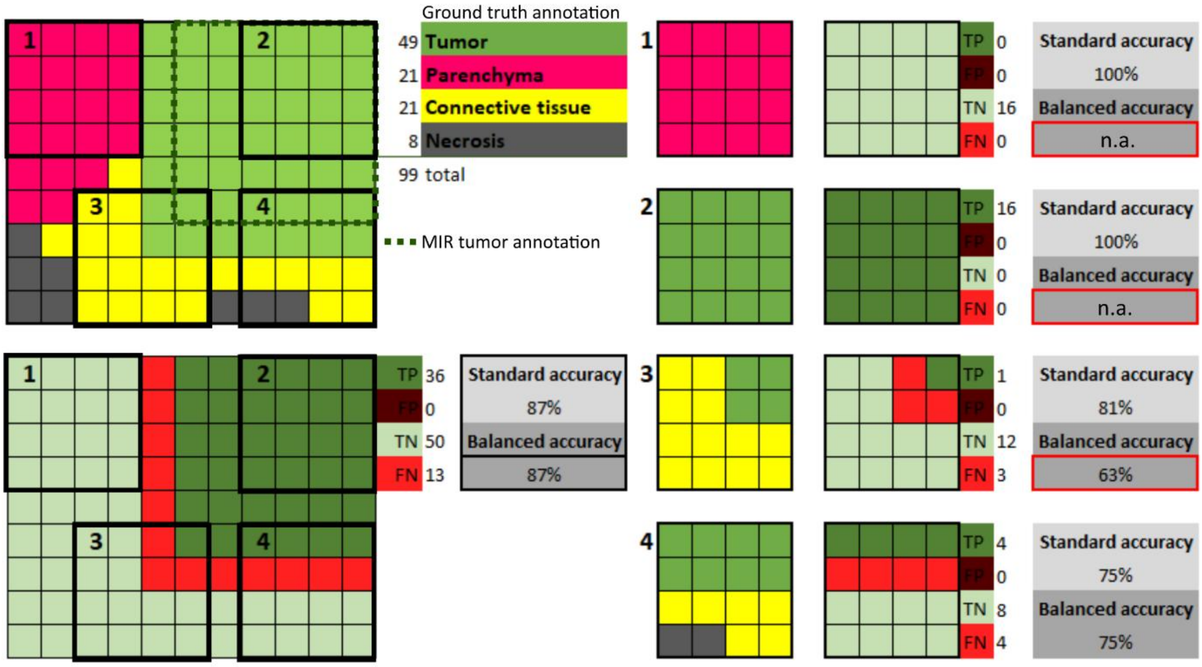

Figure S28. Choice of suitable performance metrics. Simplified simulated data for selection of suitable evaluation metric judging the performance of SA as tissue type annotation tool. Accuracy in general takes into account both, the correct assignment of TTOI (sensitivity) as well as the non-TTOI (specificity) regions. The standard accuracy is independent from class i.e. tissue type prevalence, while the balanced accuracy is taking imbalances into account. This makes the balanced accuracy dependent on representative sampling which could only be assumed via random sampling of individual pixels from a tissue. Since assignment of hundreds of individual pixels is not reasonable for pathological annotation, three rectangles per patient were selected in a way that they reflected every tissue type present in a sample (exemplified for four rectangles of different composition, top left). Due to natural localization of tissue types, rectangles did not necessarily reflect the prevalence of any tissue type in the entire sample. Thus, the standard accuracy (judging how many pixels are correctly assigned independent from tissue type prevalence) represents a more robust measure better comparable between heterogeneous samples and their subsets. As was observed for CRLM in this study, the prevalence of tissue types strongly varies between samples (estimated from MIR imaging-based tumor annotation between 4 – 38%, and other tissue types are entirely missing in some samples such as liver parenchyma). Thus, the standard accuracy was used to investigate the distribution of annotation accuracy between specimen to determine individual sample outliers (balanced accuracy cannot be calculated in cases where a tissue type is not present in a sample (rectangle 1, (division by zero not applicable, n.a.), and is strongly dependent on the prevalence of the TTOI in the rectangle, which is not necessarily representative of the entire specimen; 63% vs. 87% in rectangle 3 vs. entire sample). To judge the overall performance of SA used as a classifier for tissue type annotation combining all samples and taking tissue type imbalance of the entire cohort into account the balanced accuracy was provided for all tissue types of interest.

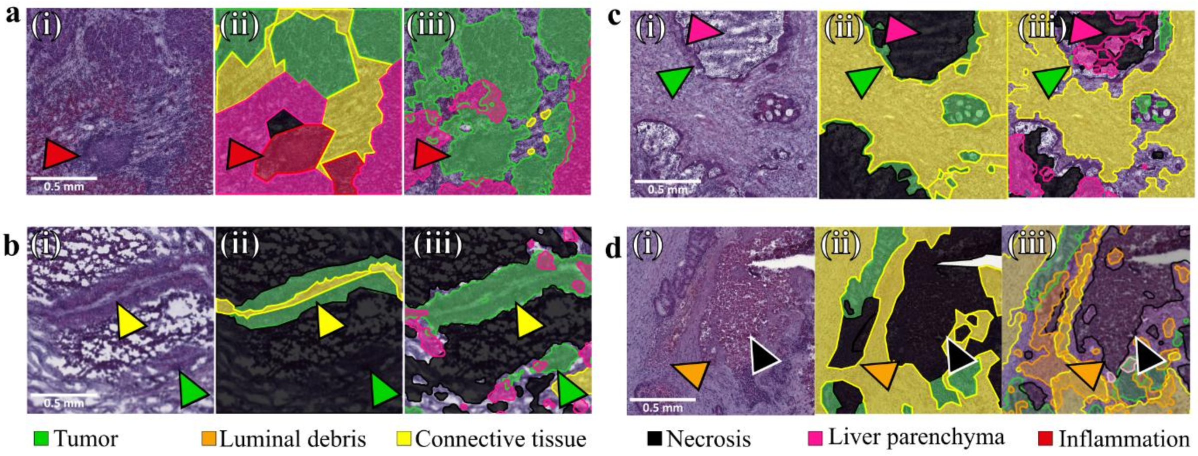

Figure S29. Common deviations of MIR based hotspot annotations from expert pathologist's annotations. Provided for each type of deviation are (i) a H&E-stained reference section, (ii) pathological annotations, and (iii) MIR hotspot annotations based on projection images of MIR imaging data. **a)** Tissue types with seemingly similar biomolecular composition, e.g., high nucleic acid content due to higher cell and thus nuclei density, as tumorous and inflammatory regions (red arrow) were insufficiently differentiated by the selected wavenumbers. **b)** The lateral step-size of MIR imaging (25 µm) used in this study prohibited detection of fine structures such as connective tissue lining tumorous cells (yellow arrow in b) or thin monolayers of tumor cells (green arrow in c). Nevertheless, molecular composition revealed small tumorous regions at the perimeter of necrotic regions which could be easily overlooked in morphological assessment (green arrow in b). **c)** Necrotic regions were often partially assigned to an intact tissue type, e.g., liver parenchyma (pink arrow), which could be a result of the remaining presence of molecular, absorbing features picked up by MIR imaging. **d)** MIR imaging differentiated luminal debris (orange) and necrosis (black), which was not differentiated via the pathological assessment in this study.

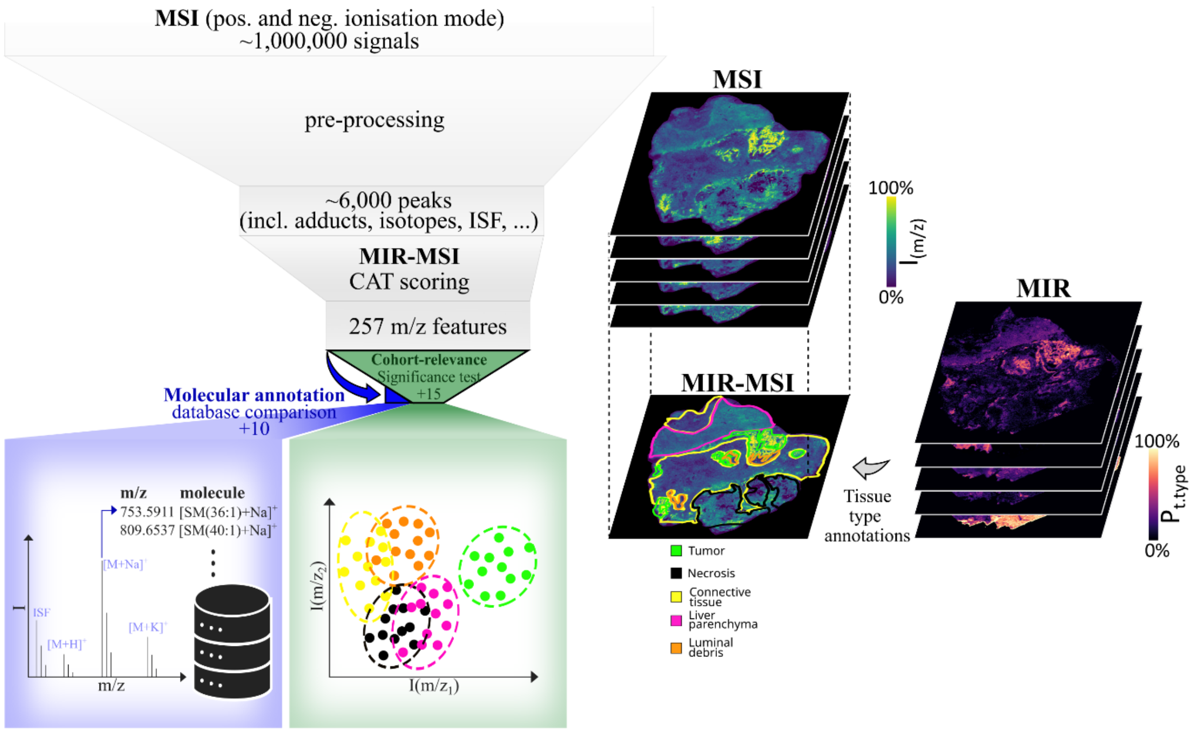

Figure S30. Schematic overview of the *m/z* feature selection process. Data pre-processing including binning reduced about 1,000,000 signals (in both ionization modes) to about 6,000 peaks. Discriminant feature analysis (correlation-adjusted t (CAT)-scoring) was performed on the MSI data in a correlative approach using the automated tissue type annotations provided by MIR imaging analysis as labels. This procedure reduced the list further to 257 candidates, which showed differential distribution between tumor and non-tumor tissues in at least one patient (grey steps). Subsequently, *m/z* feature selection from the candidate list to the most promising features for molecular annotation included the cohort-relevance (e.g., significant *m/z* features throughout ALL samples) that was investigated via Wilcoxon-rank-sum significance test (n = 12 patient means per tissue type; green, 15 features). Prior to MS/MS analysis, this list was manually expanded by features with a higher likelihood of molecular annotation by possible database assignment on MS1 level found in at least half of the patients lo-localizing with the tumorous regions (MetaSpace analysis using SwissLipids; blue, additional 10 features).

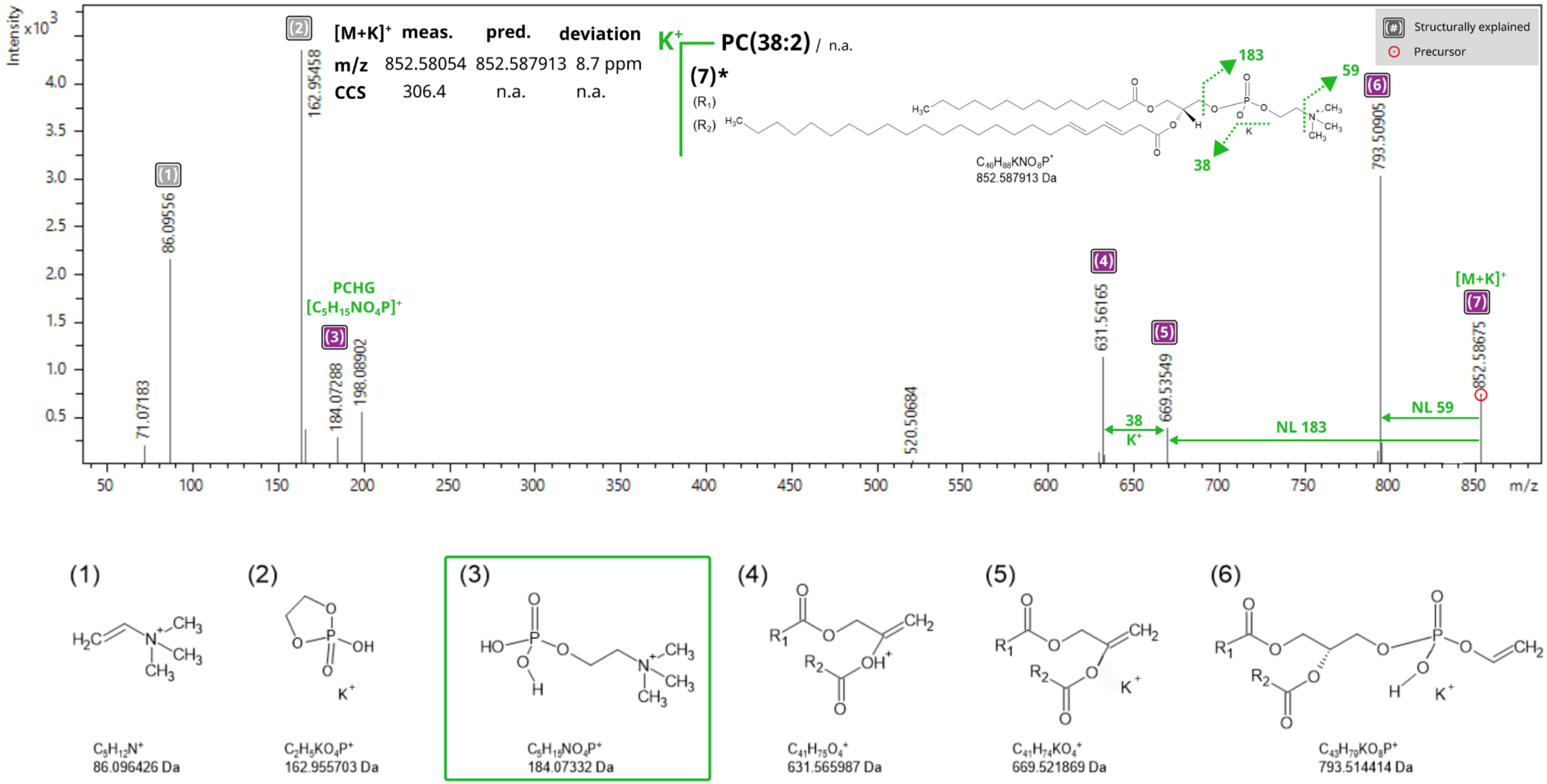

Figure S31. Fragmentation spectrum obtained via iprm-PASEF of *m/z* 852.58, annotated as PC(38:2). Neutral loss (green arrows), and estimated ion mobility values, as well as molecular structure assignment provided (corresponding numbers). Annotated head group and if available fatty acid side chain fragments (highlighted via green rectangle). *Chemical structures are only representative of the corresponding lipid; position of the double bond and chain orientation are unknown. Imaging prm-PASEF experiments were performed on a timsTOF flex mass spectrometer.

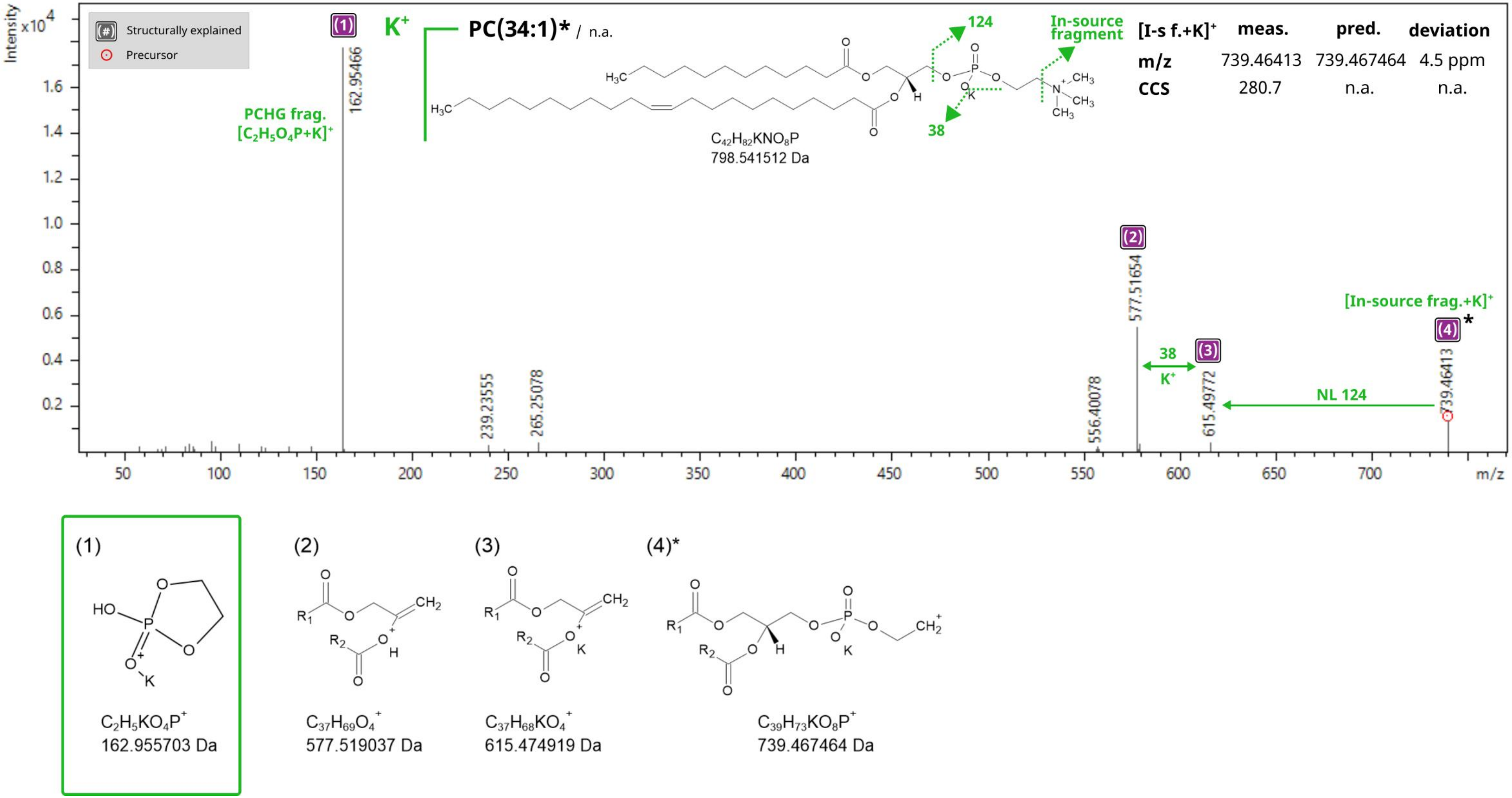

Figure S32. Fragmentation spectrum obtained via iprm-PASEF of *m/z* 739.47, annotated as PC(34:1). Neutral loss (green arrows), and estimated ion mobility values, as well as molecular structure assignment provided (corresponding numbers). Annotated head group and if available fatty acid side chain fragments (highlighted via green rectangle). *Chemical structures are only representative of the corresponding lipid; position of the double bond and chain orientation are unknown.

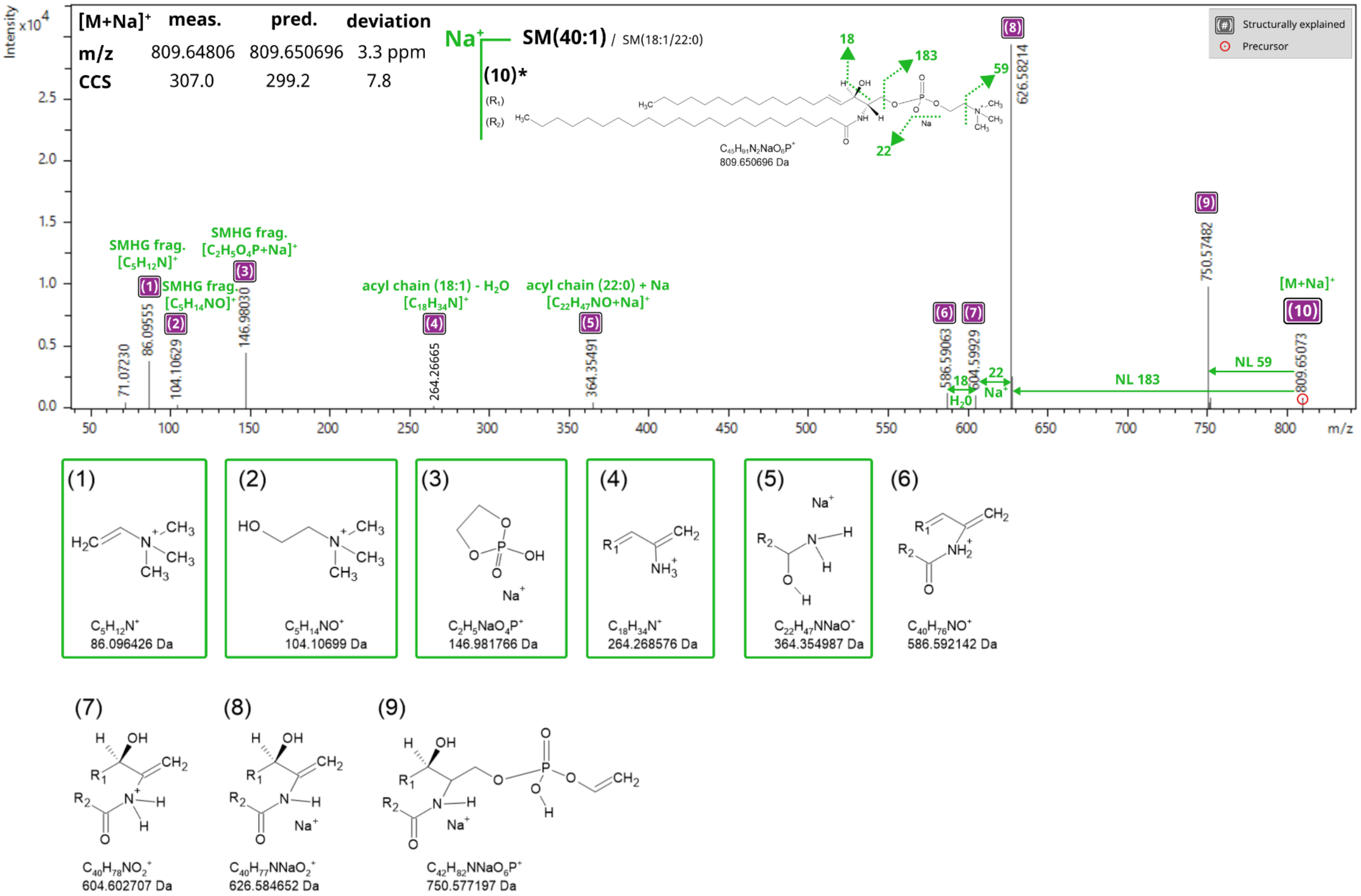

Figure S33. Fragmentation spectrum obtained via iprm-PASEF of *m/z* 809.64, annotated as SM(40:1). Neutral loss (green arrows), and estimated ion mobility values, as well as molecular structure assignment provided (corresponding numbers). Annotated head group and if available fatty acid side chain fragments (highlighted via green rectangle). *Chemical structures are only representative of the corresponding lipid; position of the double bond is unknown.

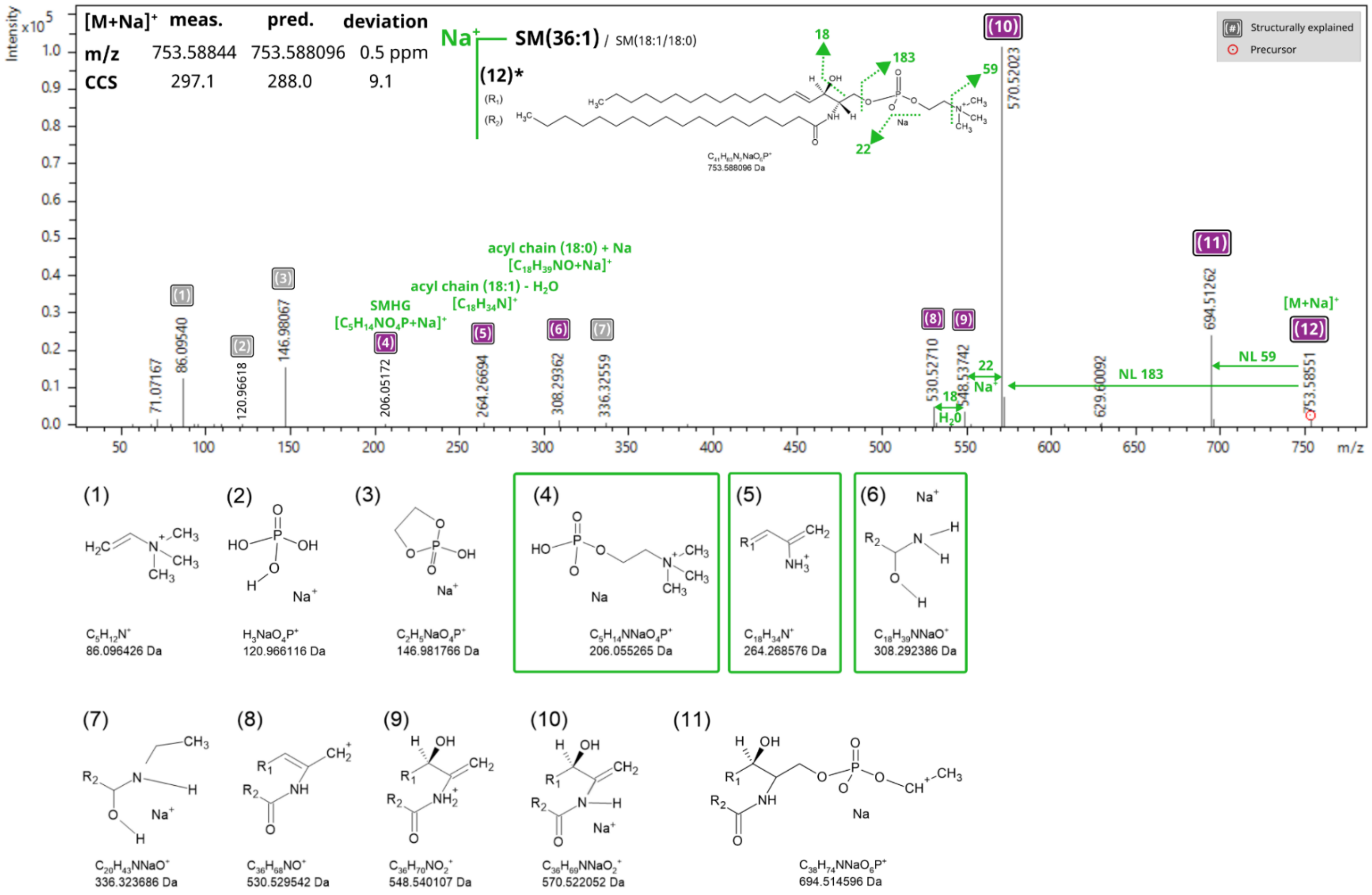

Figure S34. Fragmentation spectrum obtained via iprm-PASEF of *m/z* 753.59, annotated as SM(36:1).
Neutral loss (green arrows), and estimated ion mobility values, as well as molecular structure assignment provided (corresponding numbers). Annotated head group and if available fatty acid side chain fragments (highlighted via green rectangle). *Chemical structures are only representative of the corresponding lipid; position of the double bond is unknown.

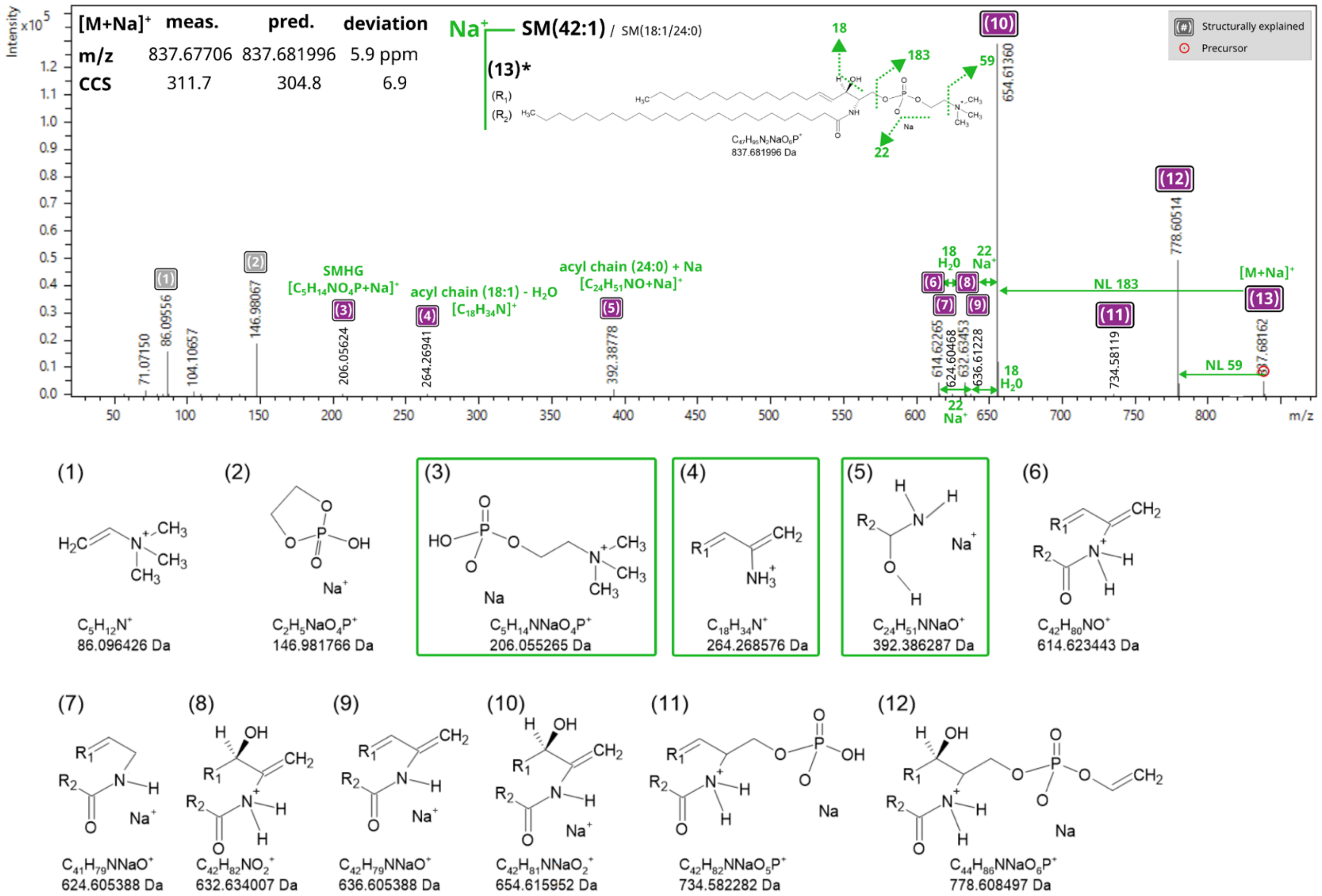

Figure S35. Fragmentation spectrum obtained via iprm-PASEF of *m/z* 837.68, annotated as SM(42:1). Neutral loss (green arrows), and estimated ion mobility values, as well as molecular structure assignment provided (corresponding numbers). Annotated head group and if available fatty acid side chain fragments (highlighted via green rectangle). *Chemical structures are only representative of the corresponding lipid; position of the double bond is unknown.

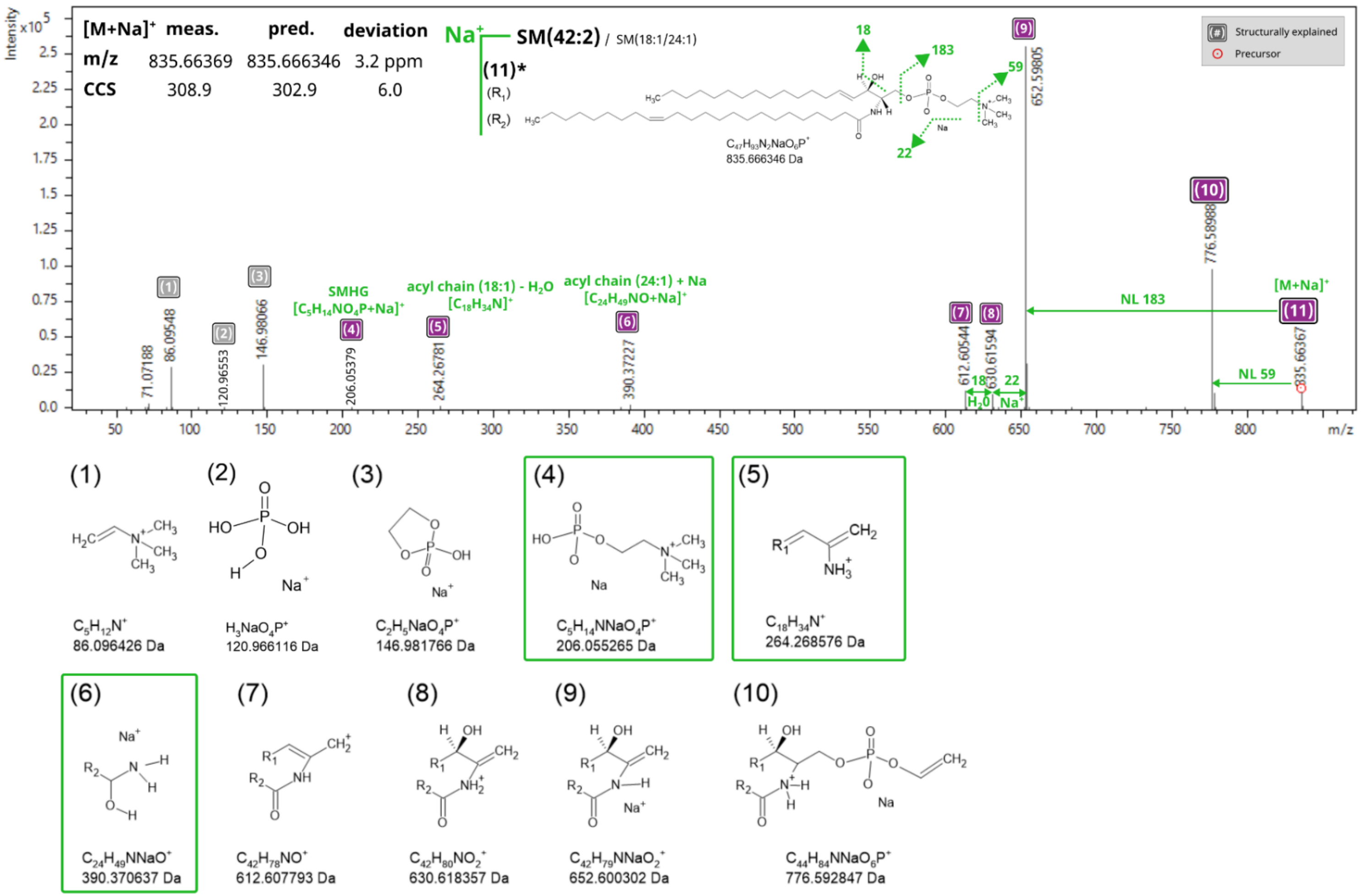

Figure S36. Fragmentation spectrum obtained via iprm-PASEF of *m/z* 835.66, annotated as SM(42:2). Neutral loss (green arrows), and estimated ion mobility values, as well as molecular structure assignment provided (corresponding numbers). Annotated head group and if available fatty acid side chain fragments (highlighted via green rectangle). *Chemical structures are only representative of the corresponding lipid; position of the double bond is unknown.

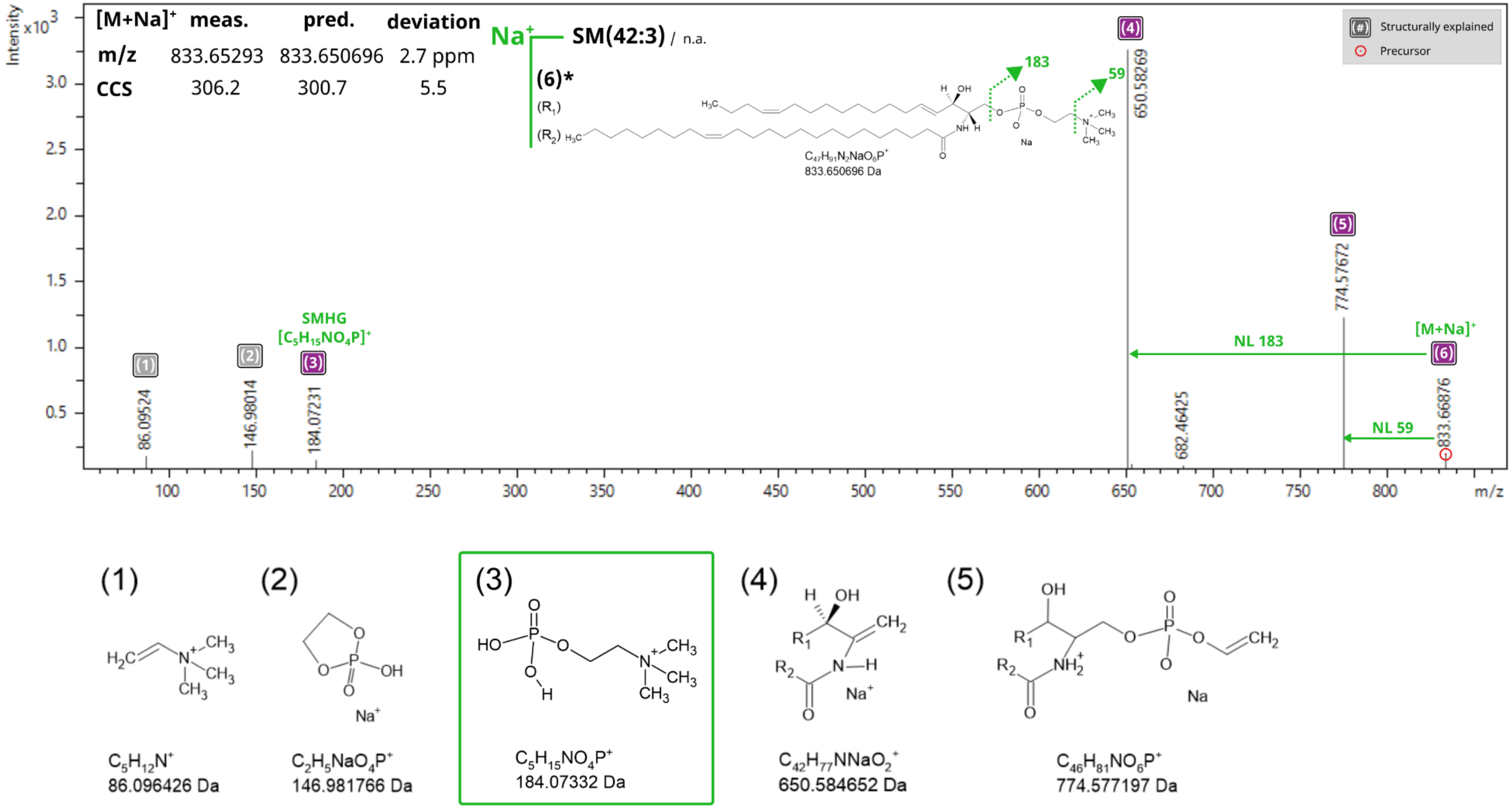

Figure S37. Fragmentation spectrum obtained via iprm-PASEF of *m/z* 833.65, annotated as SM(42:3). Neutral loss (green arrows), and estimated ion mobility values, as well as molecular structure assignment provided (corresponding numbers). Annotated head group and if available fatty acid side chain fragments (highlighted via green rectangle). *Chemical structures are only representative of the corresponding lipid; position of the double bond is unknown.

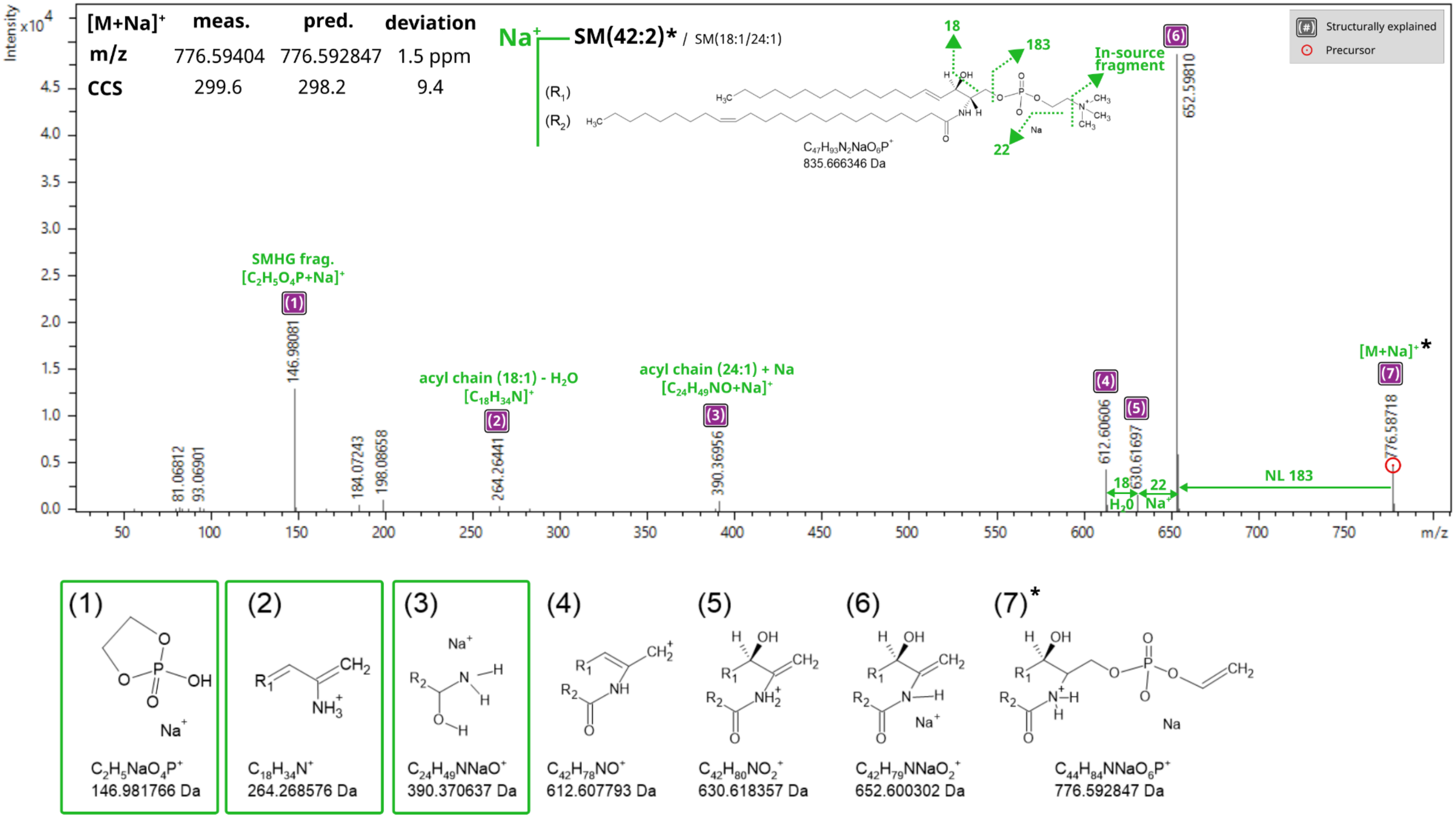

Figure S38. Fragmentation spectrum obtained via iprm-PASEF of *m/z* 776.59, annotated as SM(42:2)*. Neutral loss (green arrows), and estimated ion mobility values, as well as molecular structure assignment provided (corresponding numbers). Annotated head group and if available fatty acid side chain fragments (highlighted via green rectangle). *observed as in-source fragment. *Chemical structures are only representative of the corresponding lipid; position of the double bond is unknown.

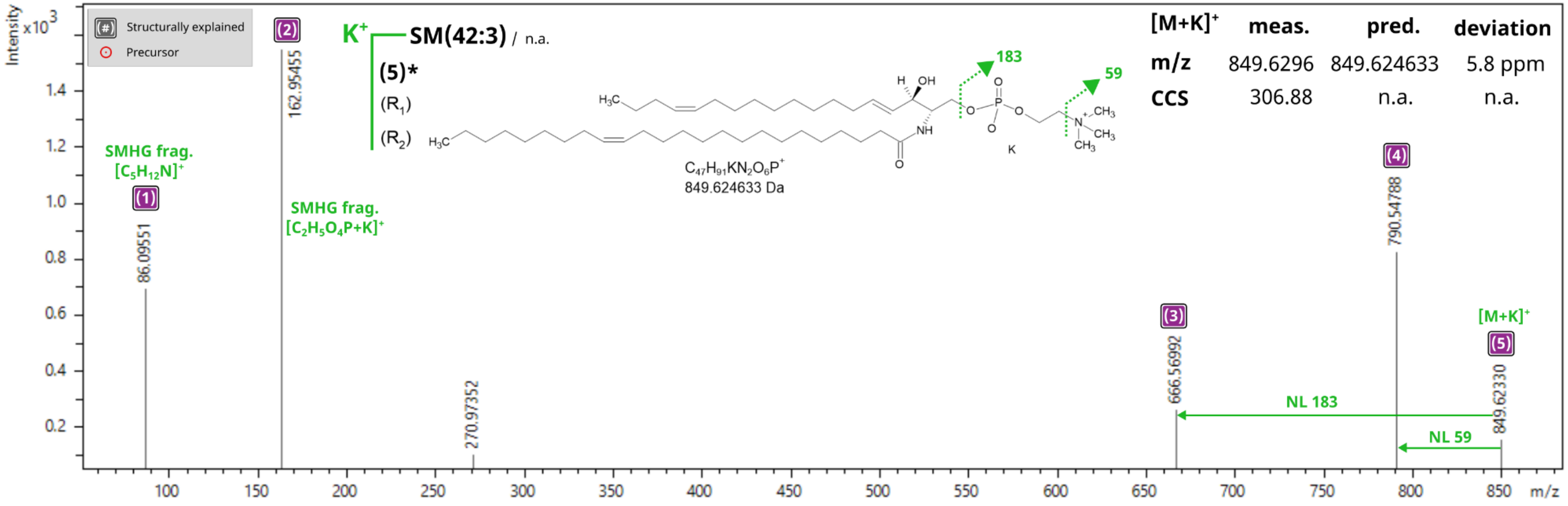

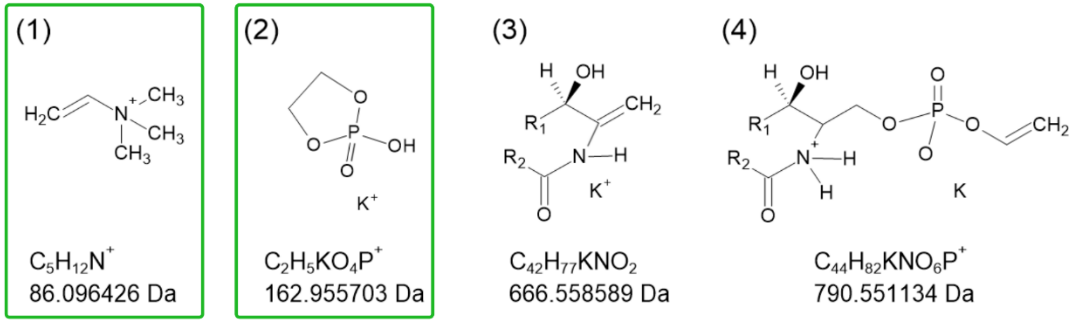

Figure S39. Fragmentation spectrum obtained via iprm-PASEF of *m/z* 849.62, annotated as SM(42:3). Neutral loss (green arrows), and estimated ion mobility values, as well as molecular structure assignment provided (corresponding numbers). Annotated head group and if available fatty acid side chain fragments (highlighted via green rectangle). *Chemical structures are only representative of the corresponding lipid; position of the double bond is unknown.

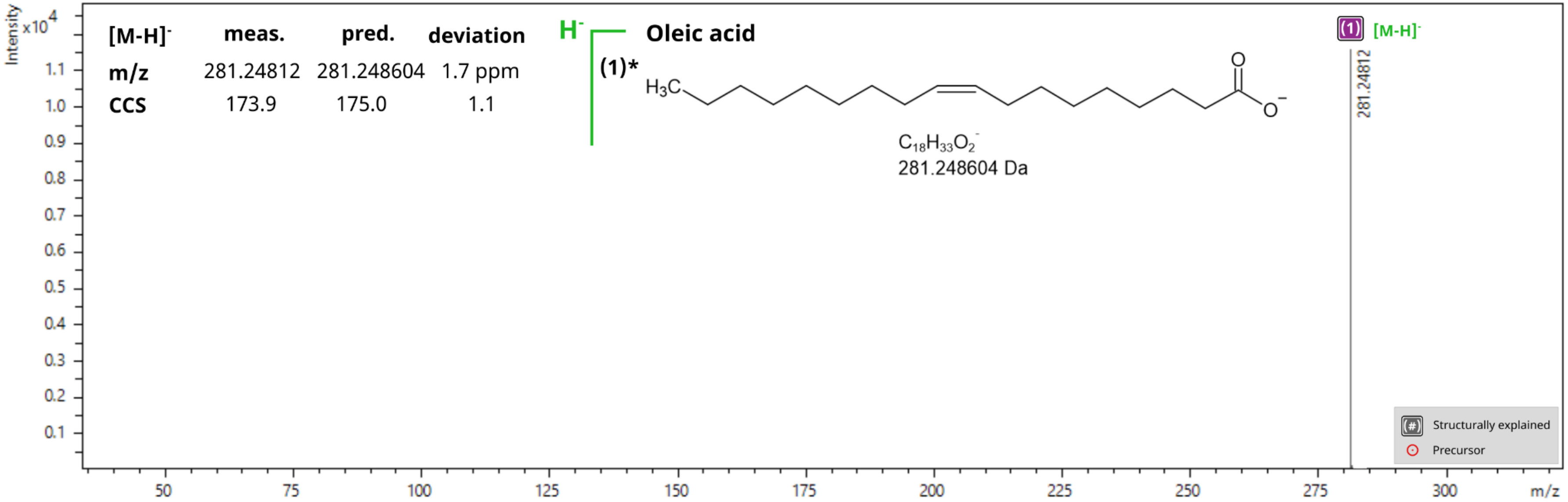

Figure S40. Fragmentation spectrum obtained via iprm-PASEF of *m/z* 281.25 annotated as oleic acid. *Chemical structures are only representative; position of the double bond is unknown.

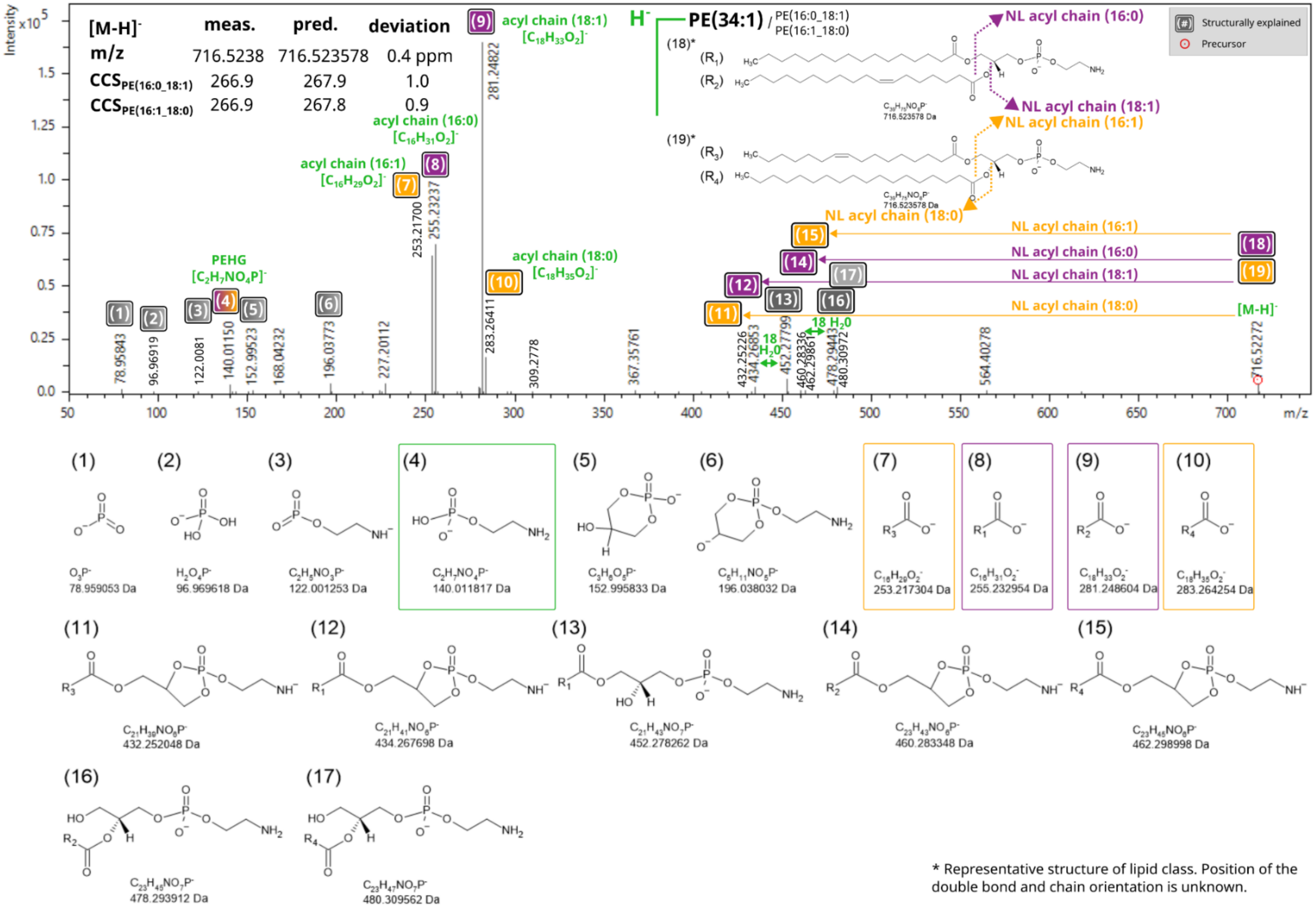

Figure S41. Fragmentation spectrum obtained via iprm-PASEF of *m/z* 716.52, annotated as PE(34:1). Neutral loss (purple, PE(16:0_18:1)) and yellow, (PE(16:1_18:0)) arrows), and estimated ion mobility values, as well as molecular structure assignment provided (corresponding numbers). Annotated common head group and fatty acid side chain fragments (highlighted via rectangles of corresponding colors). *Chemical structures are only representative of the corresponding lipid; position of the double bond and orientation of the side chains are unknown.

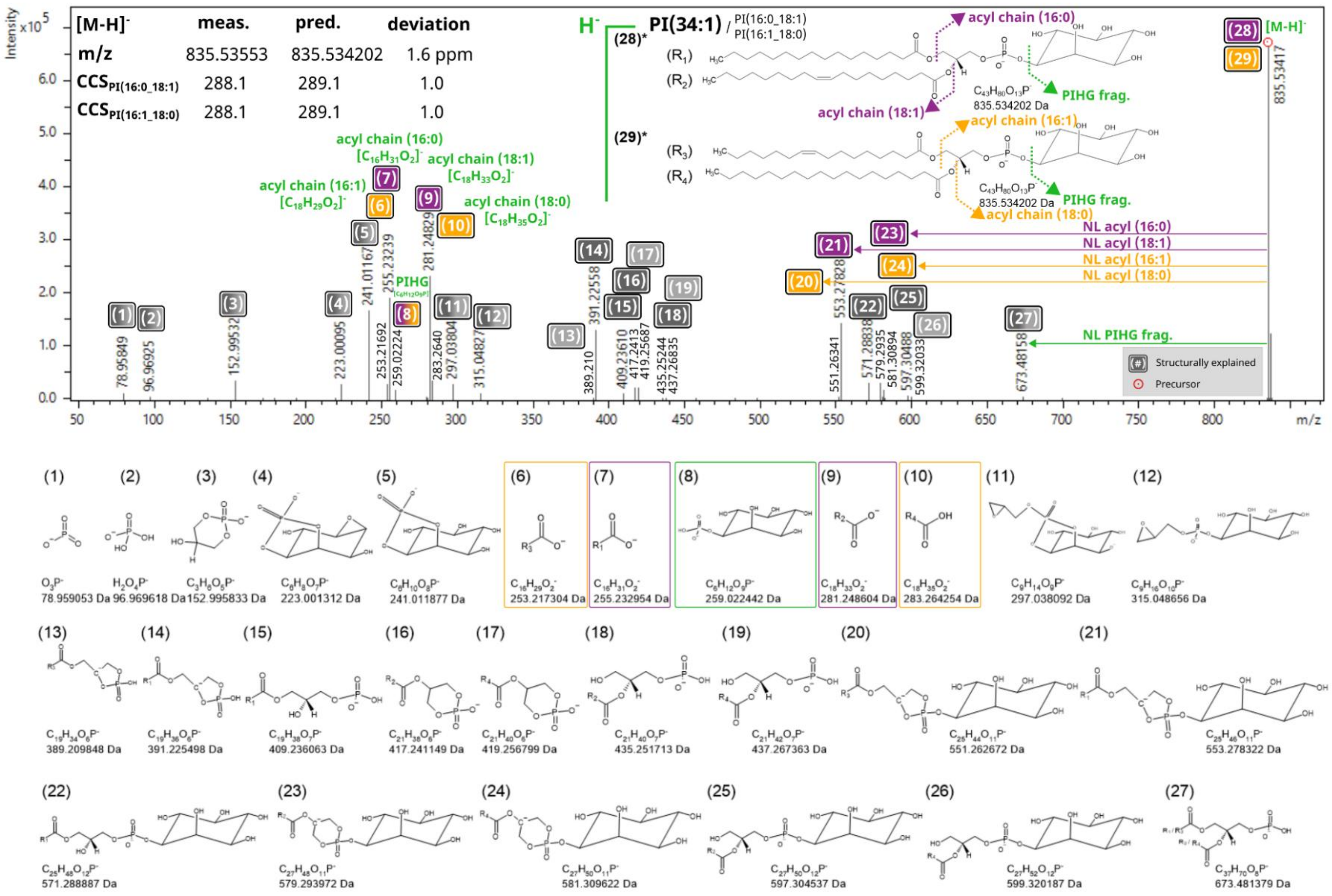

Figure S42. Fragmentation spectrum obtained via iprm-PASEF of *m/z* 835.54, annotated as PI(34:1). Neutral loss (green, head group, purple, PI(16:0_18:1)) and yellow, (PI(16:1_18:0)) arrows), and estimated ion mobility values, as well as molecular structure assignment provided (corresponding numbers). Annotated common head group and fatty acid side chain fragments (highlighted via rectangles of corresponding colors). *Chemical structures are only representative of the corresponding lipid; position of the double bond and orientation of the side chains are unknown.

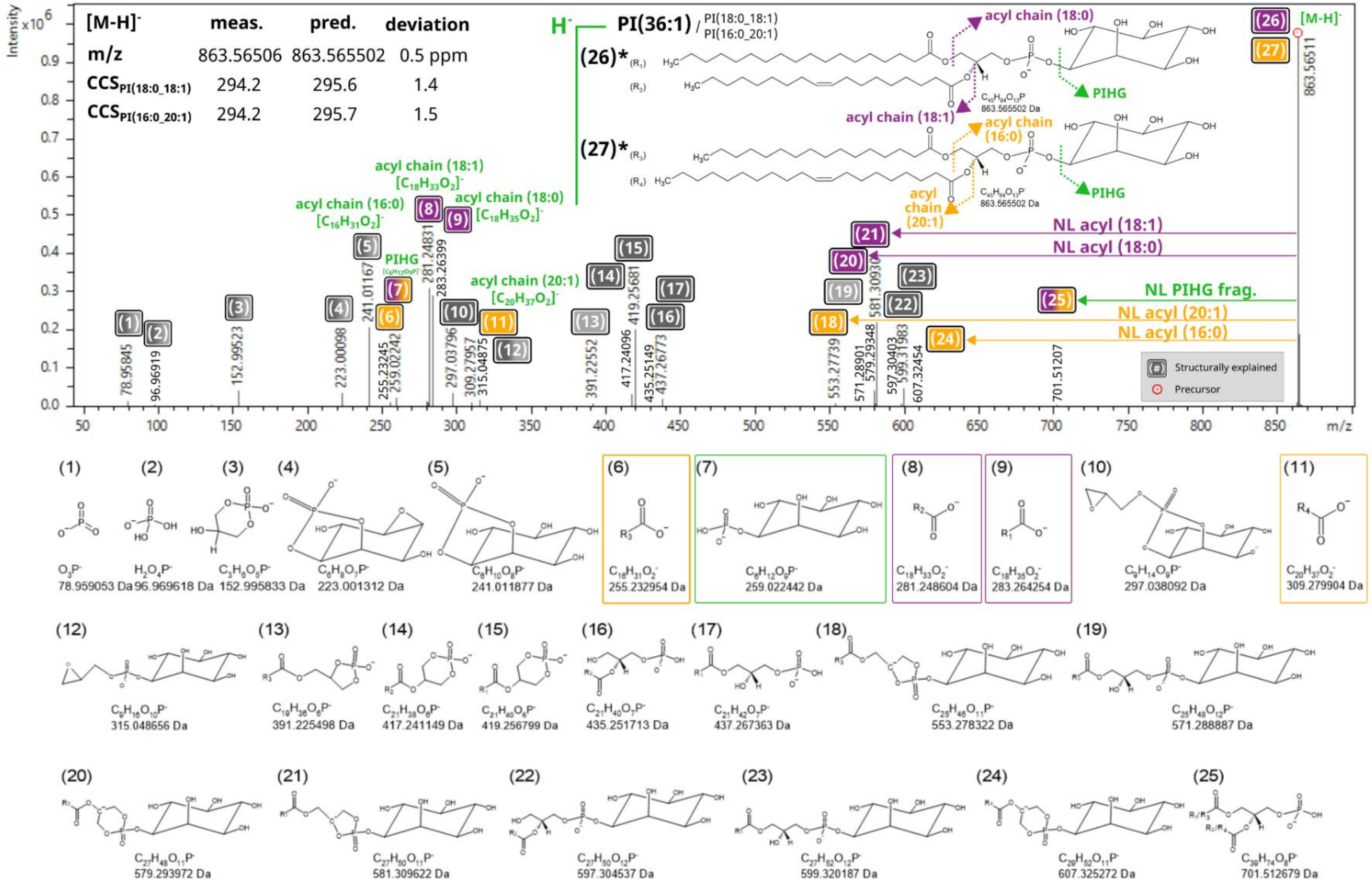

Figure S43. Fragmentation spectrum obtained via iprm-PASEF of *m/z* 863.57, annotated as PI(36:1). Neutral loss (green, head group, purple, PI(18:0_18:1)) and yellow, (PI(16:0_20:1)) arrows), and estimated ion mobility values, as well as molecular structure assignment provided (corresponding numbers). Annotated common head group and fatty acid side chain fragments (highlighted via rectangles of corresponding colors). *Chemical structures are only representative of the corresponding lipid; position of the double bond and orientation of the side chains are unknown.

Supplementary Tables

Table S1: Comparison of different algorithms used for tissue morphology annotation revealing best compromise between performance and computational implementation requirements for spatial autocorrelation analysis. Algorithm categories (A: clustering algorithms, B: machine learning algorithms, C: spatial autocorrelation) compared regarding requirement of prior knowledge, automatic class assignment, consideration of spatial context, consideration of unknown or mixed tissue type regions (gray shaded rows). Examples for each category showcasing computational requirements for implementation and according performance metrics (white rows, sorted by category).

	Algorithm / examples	Required prior knowledge	Assignment of annotation results	consideration of spatial context	consideration of unknown tissue type regions	consideration of mixed tissue type regions
**A**	**clustering algorithms**	**number of clusters**	**required from operator**	**no**	**no, assignment of most likely tissue type**	**no, assignment of most likely**
		used algorithm:	implementation:		annotation performance:	Note:
	Neumann et al. [2]	PCA (unsupervised)	number of classes: estimated via **Davies-Bouldin optimization**, cluster assignment: **visually estimated** via histological comparison		not evaluated (**judged by eye** as compared to histological image)	rat brain
	Gruber et al. [3]	k-means (unsupervised)	number of classes: **operator defined**, cluster assignment: **visually estimated** via histological comparison		not evaluated (**judged by eye** as compared to histological image or in **comparison to MSI**)	mouse different organs
	Kümmel et al. [4]	k-means (unsupervised)	number of classes: **operator defined**, cluster assignment: **visually estimated** via histological comparison		not evaluated (**judged by eye** as compared to histological image)	mouse brain and liver with HCC model
	Oinas et al. [5]	k-means (unsupervised)	number of classes: **operator defined**, cluster assignment: **visually estimated** via histological comparison		not evaluated (**judged by eye** as compared to histological image)	Osteoarthritic Human Articular Cartilage
**B**	**machine learning algorithms**	**comprehensive ground truth training data for implementation**	**automatic once trained**	**no**	**no, assignment of most likely tissue type**	**yes, assignment of different likelihoods of annotation**
		used algorithm:	implementation via training data with pathological ground truth annotations:		annotation performance:	Note:
	Nallala et al. [6]	LDA model	9 samples, 6 patients, **86,802 spectra**		average sensitivity of **89.49%**	colon adenocarcinoma
	Ferguson et al. [7]	Random forest classifier	260 tissue cores, 65 patients, **20,000 – 30,000 spectra**		model accuracy for FT-IR data **80%**, for QCL data **91%**	prostate cancer
	Liberda et al. [8]	Random forest classifier	519 tissue cores, 201 patients (spectra n.a.)		average AUC **0.93**	pancreatic cancer
**C**	**spatial auto-correlation**	**features of interest**	**automatic based on specific features**	**yes**	**yes, possible to not assign any tissue type**	**yes, assignment of multiple tissue types**
		used algorithm:	implementation (selection of discriminant wavenumbers):		annotation performance:	Note:
	Rittel et al.	Moran's I (spatial autocorrelation)	1 sample, 1 patient, **3,000 spectra**		average accuracy **87%**	colorectal cancer liver metastasis

Table S2. Random forest ranking and error testing for selection of wavenumbers that differentiate between tissue types. MIR wavenumbers were investigated for all tissue types of interest (tumor, connective tissue, necrosis, parenchyma, luminal debris, and inflammation). Wavenumbers that were top 10 in at least one repetition of the random forest ranking, were flagged for potential discriminative power. These were tested by a 25% error rate (detailed in Methods). Wavenumbers that passed error-testing were considered to have discriminant power specific to one tissue type for each tissue. Each tissue type was tested individually. For "inflammation" no specific wavenumbers were found, and this tissue type was omitted. Features in the "final wavenumber" list (green, bottom row) were mostly contained in the top5 ranking features (in at least one repetition), while only in a view cases analysis up to the top10 ranking features was successful in adding further discriminant wavenumbers (green rectangles, upper right). Thus, no further even lower ranking features were analyzed.

Tumor

Tissue type		Rank	#1	#2	#3	#4	#5	#6	#7	#8	#9	#10	-	-	-	-	-
1 & 2)Top10 RF	Run	1	960	964	1108	1712	1156	968	1076	1092	984	1028					
		2	1108	964	960	1712	1156	1028	1088	1076	2904	968					
		3	964	960	1712	1108	1076	968	1156	1028	984	1092					
		4	1108	964	1712	960	1156	1092	1032	1028	1056	1088					
		5	1108	964	960	1156	1076	1092	1712	2904	968	1088					
		6	1108	964	1712	960	1092	1156	968	984	1028	1076					
		7	964	960	1156	1108	1712	1056	968	984	1076	1152					
		8	1108	964	1156	960	1092	1076	984	1712	968	1028					
		9	1108	964	960	1712	968	1092	1156	2904	1088	1028					
		10	1108	964	960	1156	1712	1028	1092	984	2904	968	-	-	-	-	-
3)Top10 repeatition	Lambda		964	960	1108	1712	1156	968	1028	1092	1076	984	1088	2904	1056	1032	1152
	Frequency		10	10	10	10	10	9	8	8	7	6	4	4	2	1	1
4)Error of tissue types	Total error		10.2	11.1	8.3	11.3	12.1	32.7	34.1	22.1	23.2	20.6	23.6	34.0	36.4	20.1	11.3
> 25%	Others combined error		9.6	10.5	7.4	10.9	10.5	34.1	35.8	22.2	23.0	20.2	23.8	34.8	37.7	19.3	10.6
	Tumor error		17.3	18.0	19.5	16.9	18.1	16.4	14.1	21.3	25.0	25.9	21.2	24.8	20.4	29.4	20.0
	Necrosis error		3.6	50.5	0.9	0.0	9.0	0.3	49.4	10.6	43.3	2.2	0.0	0.5	5.6	42.0	0.6
	Connective tissue error		0.1	3.5	12.5	26.7	1.1	0.6	0.3	0.4	1.0	9.8	2.9	6.4	0.6	0.1	20.4
	Inflammation error		3.2	19.4	16.8	31.8	6.1	2.4	3.2	3.8	7.6	17.4	8.6	8.0	4.2	0.6	36.6
	Parenchyma error		16.7	7.0	4.5	4.3	15.2	61.7	55.9	38.3	33.2	30.6	41.5	59.4	67.5	27.3	2.5
	Luminal debris error		0.5	2.3	17.7	6.0	15.5	2.6	3.5	0.2	1.0	1.6	0.3	5.6	0.2	1.9	50.2
5) Final wavenumbers			964	-	1108	-	1156	-	-	-	-	-	-	-	-	-	-

Selected features:
Top5: 3 / 3
Top10: 3 / 3

Connective tissue

Rank	#1	#2	#3	#4	#5	#6	#7	#8	#9	#10	-	-	-	-	-
1	1032	1296	1200	1052	1092	1280	1216	1564	1232	1212	-				-
2	1296	1032	1092	1216	1220	1212	1200	1232	1036	1280	-				-
3	1032	1092	1028	1216	1220	1564	1296	1200	1280	1212	-				-
4	1296	1032	1092	1232	1220	1280	1564	1212	1200	1052	-				-
5	1032	1200	1212	1296	1216	1092	1280	1220	1232	1028	-				-
6	1200	1296	1232	1032	1564	1280	1220	1092	1212	1216	-				-
7	1032	1200	1092	1564	1220	1296	1280	1212	1300	1028	-				-
8	1092	1028	1032	1296	1280	1216	1564	1200	1220	1036	-				-
9	1092	1296	1200	1032	1280	1216	1220	1232	1212	1564	-				-
10	1296	1032	1564	1200	1280	1220	1232	1216	1092	1076	-	-	-	-	-
Lambda	1032	1296	1200	1092	1280	1220	1216	1564	1212	1232	1028	1052	1036	1300	1076
Frequency	10	10	10	10	10	9	8	8	8	7	4	2	2	1	1
Total	6.9	6.9	5.3	11.8	7.1	7.7	7.2	8.0	8.2	7.3	11.4	11.3	11.0	6.8	17.1
Others combined	6.6	5.8	4.5	11.3	4.5	6.0	5.7	6.0	8.1	5.7	12.4	11.4	10.6	5.5	19.0
Tumor	1.7	2.4	0.5	2.8	0.6	3.5	1.9	3.4	2.2	2.1	1.7	8.3	9.4	3.4	10.2
Necrosis	0.0	0.1	0.3	3.5	0.7	0.3	0.1	12.3	0.0	0.1	0.0	18.3	0.1	0.3	3.7
Connective tissue	7.7	10.2	7.6	13.4	14.7	12.6	11.6	13.6	8.5	11.7	8.3	11.0	12.3	10.7	11.3
Inflammation	76.7	89.7	93.9	48.3	87.4	95.9	95.0	86.2	91.8	96.8	73.1	49.2	58.1	92.1	57.8
Parenchyma	0.8	1.8	1.5	7.0	1.9	2.4	2.2	1.5	3.8	2.7	9.5	1.4	3.4	1.5	14.3
Luminal debris	52.4	20.0	3.3	66.7	1.7	12.7	13.0	3.6	33.9	6.0	53.7	90.7	84.7	14.7	94.5
IGNORE Inflam	-	1296	1200	-	1280	1220	1216	1564	-	1232	-	-	-	1300	-

Selected features:
Top5: 7 / 8
Top10: 8 / 8

Necrosis

Rank	#1	#2	#3	#4	#5	#6	#7	#8	#9	#10				
1	1604	1468	1628	1240	1452	1512	1664	1596	1476	1612				
2	1604	1468	1628	1452	1476	1512	1612	1664	1504	1240				
3	1604	1628	1468	1504	1452	1612	1600	1240	1664	1512				
4	1604	1452	1468	1504	1512	1476	1628	1612	1600	1240				
5	1604	1452	1468	1476	1628	1512	1664	1612	1240	1600				
6	1604	1628	1612	1468	1476	1512	1240	1504	1664	1608				
7	1604	1468	1452	1628	1476	1512	1084	1240	1664	1612				
8	1604	1468	1628	1452	1612	1512	1476	1664	1240	1596				
9	1604	1468	1628	1452	1476	1504	1512	1612	1600	1664				
10	1604	1468	1628	1452	1240	1512	1476	1664	1612	1608				
Lambda	1604	1468	1628	1512	1612	1240	1452	1664	1476	1504	1600	1596	1608	1084
Frequency	10	10	10	10	10	9	9	9	9	5	4	2	2	1
Total	11	4	3.7	4	9	3.6	10.6	11.5	2.8	5.4	8.5	10.1	11.2	6.1
Others combined	12	4.3	3.9	4	9.8	3.5	11.3	12.5	3	5.8	9.1	10.7	11.8	6.4
Tumor	6.7	3.8	2.6	7.5	2.4	7	12	3.4	4	4.9	8.2	9.6	2.4	4.1
Necrosis	1.3	1	1.2	4.5	1.7	4.6	2.8	2.3	1.3	1.1	3	4.1	5.2	3.4
Connective tissue	1.6	5.3	0.4	2	0.8	1.3	0.5	1.3	3.3	1.9	1.6	1.9	0.5	13.4
Inflammation	1.1	3.6	0.3	2.3	0	0	0.2	0.8	2.6	1.1	1.1	0.6	0	1.7
Parenchyma	18.8	3.6	5.4	4.4	15.7	2.6	17.3	20.3	2.4	8	13.4	15.4	19.9	1.8
Luminal debris	10.2	6.9	11.7	5.5	11.9	21.3	10.4	10.6	6.5	8.5	9.9	14.8	7.2	24.2
	1604	1468	1628	1512	1612	1240	1452	1664	1476	1504	1600	1596	1608	1084

Selected features:
Top5: 9 / 14
Top10: 14 / 14

Parenchyma

Rank	#1	#2	#3	#4	#5	#6	#7	#8	#9	#10	-	-	-	-	-
1	1740	1744	1136	1756	1132	1104	1760	2856	1148	1128	-				-
2	1740	1744	1136	1756	1132	1148	1760	1764	2856	1104	-				-
3	1740	1744	1136	1760	1132	2928	1756	1752	2856	2864	-				-
4	1740	1744	1136	1132	1760	1756	1148	2856	1152	1752	-				-
5	1740	1744	1136	1132	1760	1756	1752	1148	2856	1104	-				-
6	1740	1744	1136	1132	1104	1760	1752	2928	1148	1756	-				-
7	1740	1744	1148	1132	1136	1760	1756	2856	1104	2928	-				-
8	1740	1744	1132	1136	1756	1104	1760	2856	2928	1752	-				-
9	1740	1744	1132	1136	1756	1760	1104	1148	1752	2864	-				-
10	1740	1744	1136	1132	1148	1756	1752	2928	1760	2856	-	-	-	-	-
Lambda	1740	1744	1136	1756	1132	1760	2856	1148	1124	1752	2928	2864	1128	1764	1152
Frequency	10	10	10	10	10	10	8	8	7	7	5	2	1	1	1
Total	4.5	5.3	12.0	6.5	12.2	8.5	9.0	20.9	21.4	10.3	13.1	9.5	20.2	12.3	28.6
Others combined	2.7	4.3	11.0	3.8	10.3	6.6	13.4	34.4	33.1	12.1	15.8	15.3	29.7	8.3	31.6
Tumor	8.8	20.0	23.2	20.1	6.5	24.9	77.7	10.0	14.8	70.6	76.6	81.7	15.2	26.3	2.7
Necrosis	5.0	4.8	2.7	2.0	30.3	11.2	0.7	99.2	97.7	0.0	0.1	0.7	93.3	14.7	76.7
Connective tissue	0.2	0.4	9.6	0.4	5.9	0.8	1.6	24.8	21.5	0.9	3.8	3.6	17.2	2.2	29.9
Inflammation	0.0	0.0	18.6	0.0	11.3	0.0	0.2	16.0	17.1	3.0	4.8	3.9	21.2	1.8	13.0
Parenchyma	6.3	6.2	13.0	9.1	14.1	10.4	4.7	7.5	9.8	8.4	10.3	3.8	10.9	16.2	25.7
Luminal debris	2.9	0.0	9.5	0.2	0.2	1.2	1.1	9.9	8.3	2.5	14.9	0.9	1.0	1.7	10.6
	1740	1744	1136	1756	-	1760	-	-	-	-	-	-	-	-	-

Selected features:
Top5: 5 / 5
Top10: 5 / 5

Luminal debris

Rank	#1	#2	#3	#4	#5	#6	#7	#8	#9	#10	-	-	-	-
1	1168	1132	1172	1164	1308	2872	1260	2868	1076	1040				
2	1168	1132	1164	1172	1308	2872	1060	2868	1076	1128				
3	1168	1132	1172	1164	1260	2872	1308	1060	1128	1180				
4	1168	1132	1164	2868	2872	1040	1308	1076	1172	1060				
5	1168	1132	1172	1164	2872	1308	1076	1040	2868	1260				
6	1168	1132	2872	1164	1308	2868	1076	1172	1040	1072				
7	1168	1132	2872	1164	2868	1172	1076	1260	1308	1040				
8	1168	1132	2872		1164	2868	1308	1076	1260	1072				
9	1168	1132	2872	1164	1172	1308	1260	1076	1040	2868				
10	1168	1164	1132	1172	1076	2868	1308	2872	1260	1040	-	-	-	-
Lambda	1168	1132	2872	1308	1164	1172	1076	2868	1260	1040	1060	1128	1072	1180
Frequency	10	10	10	10	10	9	9	9	7	7	3	2	2	1
Total	3.6	5.8	7.0	21.9	12.3	15.9	17.3	8.0	11.2	12.4	11.5	6.7	12.0	10.1
Others combined	3.4	5.5	6.2	22.2	12.4	15.9	17.4	8.0	10.9	12.3	11.4	6.3	12.1	9.9
Tumor	8.8	13.4	6.6	34.2	4.3	25.0	5.6	4.3	14.5	10.1	5.1	18.2	8.2	5.8
Necrosis	0.5	1.1	0.0	10.9	3.1	0.2	0.0	0.5	50.6	0.3	12.8	0.0	25.1	0.2
Connective tissue	4.7	15.5	16.0	7.8	35.8	2.4	59.1	27.0	6.9	24.9	26.7	16.4	33.1	33.8
Inflammation	1.1	7.0	8.6	2.4	13.0	1.8	12.1	15.3	0.0	2.6	10.0	13.9	1.4	7.0
Parenchyma	2.6	0.0	2.3	30.7	3.6	24.9	1.8	0.1	5.5	8.9	4.5	0.1	0.2	0.5
Luminal debris	6.8	11.1	23.7	14.9	8.9	15.9	15.5	7.5	18.2	14.1	13.2	15.7	10.8	14.0
	1168	1132	2872	-	-	1172	-	-	-	1040	-	1128	-	-

Selected features:
Top5: 4 / 6
Top10: 6 / 6

Inflammation

Rank	#1	#2	#3	#4	#5	#6	#7	#8	#9	#10	-	-	-	-	-	-	-	-	-
1	1340	1232	1236	1260	1616	1304	1216	1516	1296	1280	-	-	-					-	-
2	1340	1232	1236	1216	1336	1296	1260	1220	1304	1224	-	-	-					-	-
3	1232	1340	1220	1336	1236	1280	1216	1240	1296	1324	-	-	-					-	-
4	1236	1232	1336	1340	1200	1216	1220	1264	1324	1260	-	-	-					-	-
5	1232	1236	1216	1340	1220	1336	1324	1260	1264	1616	-	-	-					-	-
6	1236	1232	1216	1336	1340	1324	1224	1220	1280	1260	-	-	-					-	-
7	1232	1220	1236	1216	1340	1324	1616	1264	1208	1260	-	-	-					-	-
8	1232	1340	1216	1236	1616	1336	1296	1260	1200	1280	-	-	-					-	-
9	1232	1220	1216	1236	1340	1336	1324	1260	1208	1296	-	-	-					-	-
10	1260	1232	1236	1340	1220	1616	1296	1280	1336	1564	-	-	-	-	-	-	-	-	-
Lambda	1340	1232	1236	1260	1216	1336	1220	1296	1324	1616	1280	1264	1304	1224	1208	1200	1516	1240	1564
Frequency	10	10	10	9	9	8	8	6	6	5	5	3	2	2	2	2	1	1	1
Total	21.0	22.6	22.1	24.8	23.9	21.6	24.8	24.8	24.3	24.5	26.5	23.7	22.9	23.5	21.9	23.8	28.9	21.9	25.4
Others combined	21.5	23.1	22.6	25.2	24.4	22.0	25.3	25.2	24.7	24.8	27.0	24.1	23.3	23.8	22.1	24.2	29.6	22.3	25.8
Tumor	3.1	0.9	2.0	10.2	1.5	4.5	3.8	2.2	4.8	34.9	3.1	23.4	4.0	5.1	6.7	0.3	33.0	7.2	4.0
Necrosis	2.3	0.0	0.0	0.3	0.1	2.0	0.3	0.1	0.9	0.0	4.7	0.0	4.3	0.8	13.4	0.0	0.2	0.0	14.7
Connective tissue	72.7	84.3	81.3	67.1	87.2	74.4	88.4	89.3	63.4	70.0	92.0	64.2	82.3	81.6	73.3	90.1	67.1	75.8	88.1
Inflammation	5.6	5.1	4.4	10.6	6.1	7.4	3.2	11.0	8.9	11.5	5.1	10.6	9.2	11.3	14.5	8.5	5.6	8.2	10.6
Parenchyma	3.9	1.7	1.9	13.2	1.9	3.9	2.6	1.6	12.3	6.3	4.2	10.5	2.0	2.5	1.8	1.2	17.8	3.2	1.8
Luminal debris	1.1	4.1	6.1	1.7	11.3	2.0	13.5	18.5	1.5	10.0	5.0	1.0	2.1	17.0	5.8	2.0	3.4	6.2	4.6
	-	-	-	-	-	-	-	-	-	-	-	-	-	-	-	-	-	-	-

Selected features:
Top5: 0 / 0
Top10: 0 / 0

Table S3. Overview of molecular annotations assigned via on-tissue fragmentation analysis using iprm-PASEF. Listed are ionization mode, prevalence in tumor (increased/decreased intensities in tumor vs. all other tissue types were assigned a positive/negative CAT score), measured *m/z* value, added by significance testing (S) or possible molecular annotation for at least half of the patients on MS1 level (A), analysis level (1 = suitable intensity for fragmentation, 2 = no interfering peaks in isolation window, 3 = head group fragment observed, 4 = fatty acid side chains observed), molecular annotation and adduct form based on fragmentation (MS2 level).

#	Ion. mode	CAT score	m/z value _mobility value	Added by		Analysis levels				Annotation via MS2	
				S	A	1	2	3	4	Lipid	Ion
1	POSITIVE	pos / TUMOR↑	852.5868	x	x	x	x	x		PC(38:2)	[M+K]+
2			853.5929	x							n.a.
3			740.4735		x						n.a.
4			739.4698		x	x	x			PC(34:1)	[ISF+K]+
5			799.5469		x						n.a.
6		neg / TUMOR↓	809.6537	x	x	x	x	x	x	SM(40:1)	[M+Na]+
7			750.5802	x							n.a.
8			753.5911	x		x	x	x	x	SM(36:1)	[M+Na]+
9			837.6828	x		x	x	x	x	SM(42:1)	[M+Na]+
10			825.6275	x							n.a.
11			836.6725	x							n.a.
12			835.669	x		x	x	x	x	SM(42:2)	[M+Na]+
13			833.6542	x		x	x	x		SM(42:3)	[M+Na]+
14			770.5681	x							n.a.
15			723.5439	x							n.a.
16			776.5962	x		x	x	x	x	SM(42:2)	[ISF+Na]+
17			849.6279	x		x	x	x		SM(42:3)	[M+K]+
18			769.5625	x							n.a.
19			711.5436		x						n.a.
20			839.6959		x						n.a.
21 (1)	NEGATIVE	pos / TUMOR↑	281.2486_0.8		x	x	x	x	x	Oleic acid	[M-H]-
21 (2)			281.2486_1.0								n.a.
22			716.5237		x	x	x	x	x	PE(34:1)	[M-H]-
23			835.5335		x	x	x	x	x	PI(34:1)	[M-H]-
24			415.2253		x						n.a.
25			863.5641		x	x	x	x	x	PI(36:1)	[M-H]-

Table S4. Human samples and metadata. Each sample was labelled with an internal pseudonymized ID that was used throughout this study. Tissue specimen were obtained from patients diagnosed with colorectal cancer liver metastasis (CRLM) and only retrieved from the metastases, not from the primary tumor. Wherever possible, a control sample suspected to be liver parenchyma was additionally obtained from close proximity of the tumor nodule. Histopathological diagnosis was provided by the Department of Pathology of the University Clinic Mannheim.

Internal SampleID	Used for evaluation of	Histopathological diagnosis	Disease stage	Gender	Age at surgery	Freezing technique	Additional information
ID5a	cohort-wide SA	Lebermet. AdenoCa CRCa	Stage4: liver metastasis	f	80	(f)CO2	Hep.C-
ID7b	cohort-wide SA	Lebermetastase CRCa	Stage4: liver metastasis	m	76	(f)CO2	Hep.C-
ID9	cohort-wide SA	Lebermetastase CRCa	Stage4: liver metastasis	m	33	(f)CO2	Hep.C-
ID10	cohort-wide SA	Lebermetastase CRCa	Stage4: liver metastasis	m	74	(l)N2	Hep.C-
ID18a	cohort-wide SA	Lebermet. AdenoCa CRCa	Stage4: liver metastasis	m	73	(l)N2	Hep.C-
ID19	cohort-wide SA	Lebermetastase CRCa	Stage4: liver metastasis	f	79	(l)N2	Hep.C-
ID21	reference-based SA	Lebermet. AdenoCa CRCa	Stage4: liver metastasis	m	62	(l)N2	Hep.C-
ID25	reference-based SA	AdenoCa cribriform, Nebenbefund Sigmadivertikulose	Stage4: liver metastasis	m	71	n.a.	Hep.C-
ID27	reference-based SA	Lebermet. AdenoCa CRCa partiell nekrotisch	Stage4: liver metastasis	m	71	(l)N2	Hep.C-
ID30	reference-based SA	Lebermet. AdenoCa CRCa	Stage4: liver metastasis	f	78	(l)N2	Hep.C-
ID31	reference-based SA	Lebermet. AdenoCa CRCa	Stage4: liver metastasis	m	77	(l)N2	Hep.C-
ID32	reference-based SA	Lebermetastase CRCa	Stage4: liver metastasis	m	60	(l)N2	Hep.C-

Supplementary References

(1) Lovergne, L.; Ghosh, D.; Schuck, R.; Polyzos, A. A.; Chen, A. D.; Martin, M. C.; Barnard, E. S.; Brown, J. B.; McMurray, C. T. An infrared spectral biomarker accurately predicts neurodegenerative disease class in the absence of overt symptoms. *Sci Rep* **2021**, *11* (1), 15598. DOI: 10.1038/s41598-021-93686-8.

[2] [Neumann EK, Comi TJ, Spegazzini N, Mitchell JW, Rubakhin SS, Gillette MU, Bhargava R, Sweedler JV: Multimodal Chemical Analysis of the Brain by High Mass Resolution Mass Spectrometry and Infrared Spectroscopic Imaging. Analytical chemistry 90: 11572–11580, 2018. https://doi.org/10.1021/acs.analchem.8b02913]

[3] [Gruber L, Schmidt S, Enzlein T, Vo HG, Bausbacher T, Cairns JL, Ucal Y, Keller F, Kerndl M, Sammour DA, Sharif O, Schabbauer G, Rudolf R, Eckhardt M, Iakab SA, Bindila L, Hopf C: Deep MALDI-MS spatial omics guided by quantum cascade laser mid-infrared imaging microscopy. Nature communications 16: 4759, 2025. https://doi.org/10.1038/s41467-025-59839-3]

[4] [Kümmel T, van Marwick B, Rittel M, Ramallo Guevara C, Wühler F, Teumer T, Wängler B, Hopf C, Rädle M: Rapid brain structure and tumour margin detection on whole frozen tissue sections by fast multiphotometric mid-infrared scanning. Scientific reports 11: 11307, 2021. https://doi.org/10.1038/s41598-021-90777-4]

[5] [Oinas J, Rieppo L, Finnilä MAJ, Valkealahti M, Lehenkari P, Saarakkala S: Imaging of Osteoarthritic Human Articular Cartilage using Fourier Transform Infrared Microspectroscopy Combined with Multivariate and Univariate Analysis. Scientific reports 6: 30008, 2016. https://doi.org/10.1038/srep30008]

[6] [Nallala J, Diebold MD, Gobinet C, et al. Infrared spectral histopathology for cancer diagnosis: a novel approach for automated pattern recognition of colon adenocarcinoma. Analyst. 2014;139(16):4005-4015. doi:10.1039/c3an01022h]

[7] [Ferguson D, Kroeger-Lui N, Dreisbach D, Hart CA, Sanchez DF, Oliveira P, Brown M, Clarke N, Sachdeva A, Gardner P: Full fingerprint hyperspectral imaging of prostate cancer tissue microarrays within clinical timeframes using quantum cascade laser microscopy. The Analyst 150: 1741–1753, 2025. https://doi.org/10.1039/d5an00046g]

[8] [Liberda D, Koziol P, Wrobel TP: Comprehensive Histopathology Imaging in Pancreatic Biopsies: High Definition Infrared Imaging with Machine Learning Approach. International journal of biological sciences 19: 3200–3208, 2023. https://doi.org/10.7150/ijbs.83068]
